# Pollen morphology and variability of native and alien, including invasive, species of the genus *Spiraea* L. (Rosaceae) in Poland

**DOI:** 10.1371/journal.pone.0273743

**Published:** 2022-08-29

**Authors:** Dorota Wrońska-Pilarek, Mateusz Sowelo, Wojciech Antkowiak, Jan Bocianowski, Kacper Lechowicz

**Affiliations:** 1 Department of Botany and Forest Habitats, Poznań University of Life Sciences, Poznań, Poland; 2 Department of Botany, Poznań University of Life Sciences, Poznań, Poland; 3 Department of Mathematical and Statistical Methods, Poznań University of Life Sciences, Poznań, Poland; Institute for Biological Research, University of Belgrade, SERBIA

## Abstract

The pollen morphology was studied in 25 taxa of the genus *Spiraea* L. The aims of this study were to describe the pollen morphology and variability and to determine whether the pollen features of alien, expansive or invasive *Spiraea* species differ from those of other taxa. The species of *Spiraea* were analysed for nine quantitative pollen traits as well as the following qualitative traits: the outline, shape and exine ornamentation. In total, 750 pollen grains were measured. Based on the pollen key exine ornamentation features, then individual *Spiraea* species were distinguished, while the other species formed groups of usually 2–3, up to 8 species. The most important pollen features included length, width and course of grooves and striae, presence or absence of perforations, as well as their number and diameter. The most variable taxa for all the nine biometric traits jointly were *S*. *×billardii*, *S*. *veitchii*, *S*. *nipponica* and *S*. *cana*. The pollen of the invasive *S*. *tomentosa* differed from the other taxa studied, unlike the other invasive species (*S*. *douglasii* and *S*. *japonica*).

## Introduction

The genus *Spiraea* L. belongs to the tribe Spiraeeae, the family Rosaceae Juss., to the order *Rosales* and to the rosid clade [1–4]. The taxonomy introduced by Rehder [5] divides the genus *Spiraea* into three sections: *Chamaedryon* Ser., *Calospira* K. Koch and *Spiraria* Ser. However, this was later developed by Yu and Kuan [6] into four sections and 10 series.

There are 80 to 100 species of this genus throughout the world, although the exact number of *Spiraea* species has not been specified [3, 7]. *Spiraea* is by far the largest and most widespread genus in the tribe Spiraeeae [3], which are native to the temperate Northern Hemisphere, with the greatest diversity in eastern Asia, mainly China, where approx 70 *Spiraea* species (47 endemic) are found [1, 7–9]. In Europe there are seven native species of *Spiraea*–*S*. *cana*, *S*. *chamaedryfolia*, *S*. *crenata*, *S*. *decumbens*, *S*. *hypericifolia*, *S*. *media* and *S*. *salicifolia* [10, 11].

Representatives of the genus *Spiraea* are very popular decorative plants in North America, Asia and Europe, including Poland. In the latter, many species of meadowsweet from Asia and North America are cultivated, while some of them, e.g. *S*. *tomentosa*, *S*. *douglasii*, and *S*. *japonica* L., have become naturalized and are invasive in some European countries [12–14], posing a threat to biodiversity in these areas [15].

According to Lambdon et al. [16] and the DAISIE [17], 21 alien *Spiraea* taxa (13 species and 8 hybrids) grow in Europe, of which 18 taxa were ‘aliens in Europe’ naturalized within the continent and 12 species were naturalized neophytes within the continent. A further five species were ‘aliens of European origin’ (five naturalized and three—naturalized neophytes). The third group–‘aliens to Europe’ consisted of 16 species (13 naturalized and nine—naturalized neophytes). Mirek et al. [18] reported the occurrence of two native species (*S*. *salicifolia* L., and *S*. *media* Schmidt) in Poland and the following eight alien species and hybrids: *S*. *albiflora* Zabel, *S*. *chamaedryfolia* L. em. Jacq., *S*. *hypericifolia* L., *S*. *japonica* L. f., *S*. *ulmifolia* Scop., *S*. *×vanhouttei* (Briot) Zabel, S. *×arguata* Zabel and *S*. *×bumalda* Burv. In turn, Tokarska-Guzik et al. [15] listed six domestic, alien meadowsweet species and cultivars in Poland. These were locally established kenophytes—*S*. *alba* Du Roi (North America), *S*. *chamaedryfolia* L. em. Jacq. (southeastern Europe and southeastern Asia) and *S*. *douglasii* Hook. (western North America), a domestic, invasive species—*S*. *tomentosa* L. (from eastern North America), and two locally established cultivars—*S*. *×pseudosalicifolia* Silverside (= *S*. *salicifolia* L. × *S*. *douglasii* Hook) and *S*. *×vanhouttei* (Briot) Zabel. *S*. ×*pseudosalicifolia* Silverside and *S*. ×*billardii* Hérincq, analysed by our team, are often treated synonymously and traded as the same taxon. This is quite unfortunate because *S*. ×*pseudosalicifolia* Silverside is a hybrid of *S*. *salicifolia* L. × *S*. *douglasii* Hook, while *S*. ×*billardii* Hérincq is a hybrid of *S*. *alba* Du Roi × *S*. *douglasii* Hook [19].

Many palynologists believe that palynomorphological characteristics tend to be more useful to distinguish higher ranking taxa of the family Rosaceae (subfamily, tribe or genus) rather than those of lower rank (section, series or species) [20–41]. Especially exine sculpture was found to be an important feature to distinguish species of many genera from the family Rosaceae. The most important traits of exine sculpture were the number and width of striae and grooves, as well as the number, area and size of perforations [20–34, 41]. Many scientists have emphasized that for species delimitation within the family Rosaceae the length of polar axis (P), pollen shape (P/E ratio), operculum structure as well as the presence or lack of costae colpi are very important [20–26, 28, 29, 36, 37, 41, 42].

So far, the few studies on the pollen morphology of the genus *Spiraea* have been regional in character and covered at most a dozen species. Polyakova and Gataulina [38] described the pollen morphology of 12 *Spiraea* species from different regions of Siberia and the Far East. Hebda and Chinnappa [23] described some *Spiraea* species growing in Canada. In turn, Liu et al. [39] analyzed 18 Chinese *Spiraea* species, while Song et al. [40] described 17 Korean species. Wrońska-Pilarek et al. [32] examined pollen morphology and intraspecific variability of invasive *S*. *tomentosa* in Poland. In the PalDat database, Heigl [43] and Auer [44] briefly described *S*. *chamaedryfolia* and *S*. *oblongifolia*. All authors agree that the most important pollen features of the *Spiraea* species were connected with exine ornamentation, although some researchers also mentioned other features, such as the endoaperture diameter, equatorial and polar views, and length of the polar axis (P).

The presented study aimed to describe and analyze pollen morphology and intrageneric and interspecific variability of 25 *Spiraea* taxa—two species native to Poland and Europe and 23 alien species and hybrids—which were naturally distributed in Asia, Europe and North America, whence they came to Poland. This is the first palynological review of research on this genus in Europe. This study was also aimed at determining whether the pollen features of expansive and invasive *Spiraea* species differ from those of other taxa, and if so, whether and to what extent it may increase the effectiveness of the expansion of these species. It is believed that, due to the invasive nature of some of the studied species in Poland and Europe, any new data on its reproduction may be useful in the fight against the expansion of this species.

## Material and methods

### Palynological analysis

Inflorescences of the 25 *Spiraea* taxa under analysis (22 species, one variety and two hybrids) were collected from the herbarium belonging to the Botanical Garden of the Adam Mickiewicz University in Poznań (OB UAM) (Table 1). The plants grown there come from other botanical gardens or natural sites of individual *Spiraea* species (Table 1). All samples were taken from herbarium sheets prepared in 2019. A total of 24 samples came from shrubs not older than 40 years. The youngest was 12 years old, while the oldest was 39. Additionally, four samples came from shrubs that are 48, 55, 82 and 86 years old. These are plants (except for *S*. *tomentosa* L.), which are growing scattered over an area of about 22 ha. Their flowering takes place over a period of three months (from April to June/July).

**Table 1:** List of *Spiraea* taxa under analysis collected from the herbarium of the Botanical Garden of the Adam Mickiewicz University in Poznań and natural sites.

No.	Taxon name	The area of natural occurrence	Source and year of acquisition	BG AMU registration number	Herbarium sheet number
1	*S*. *alba* Du Roi	Canada, United States	Lack of data, 1995	7312	XI
2	*S*. *alba* var. *latifolia* (Aiton) Dippel	Canada, United States	Lack of data	0995	XXVIII
3	*S*. *betulifolia* Pall.	Japan, Russia	Experimentelle Botanische Garten, Georg-August-Universität Göttingen, 2004	7244	VI
4	*S*. *cana* Waldst. & Kit	Italy, Croatia, Bosna and Herzegovina, Serbia	Hesse Nursery, Weener on Ems, 1939	1206	XV
5	*S*. *chamaedryfolia* L.	Italy, Austria, Czech Republic, Slovakia, Bulgaria, Bosna and Herzegovina, Serbia, Croatia, Romania, Russia, Mongolia, China, Korea, Japan	The Kórnik Arboretum, 1983	2561	I
6	*S*. *chinensis* Maxim.	China	Lack of data, 2000	5014	XVI
7	*S*. *dasyantha* Bunge	China	The Botanical Garden of the University of Tartu, 1989	3333	XXVII
8	*S*. *douglasii* Hook.	Canada, United States	Lack of data, 2009	8586	X
9	*S*. *elegans* Pojark.	Russia, Mongolia, China	The Akureyri Botanical Garden, 1991	5083	XXIV
10	*S*. *henryi* Hemsl.	China	The Kórnik Arboretum, lack of data	0694	XX
11	*S*. *hypericifolia* L.	Bulgaria, Ukraine, Russia, Kazakhstan, Uzbekistan, Turkmenistan, Kyrgyzstan, Tajikistan, Mongolia, China	Lack of data, 1963	0907	XIII
12	*S*. *japonica* L. f.	Japan, Korea, China	Lack of data	6394	V
13	*S*. *media* Schmidt	Austria, Czech Republic, Slovakia, Hungary, Bulgaria, Romania, Croatia, Bosna and Herzegovina, Montenegro, Poland, Ukraine, Russia, Mongolia, China, Korea, Japan	Lack of data	0880	XVII
14	*S*. *nipponica* Maxim.	Japan	Lack of data, 1991	4175	XIX
15	*S*. *pubescens* Turcz.	Mongolia, Russia, China, Korea	Lack of data	6448	XXVI
16	*S*. *salicifolia* L.	Austria, Hungary, Romania, Bulgaria, Czech Republic, Poland, Russia, Mongolia, China, Korea, Japan	Arboretum Waasland Nieuwkerken-Waas, 1994	5088	IX
17	*S*. *splendens* É. N. Baumann ex K. Koch	Canada, United States	Lack of data	2547	XXIX
18	*S*. *thunbergii* Siebold ex Blume	China, Japan	Mlyňany Arboretum of the Slovak Academy of Sciences, 1984	2609	XII
19	*S*. *trichocarpa* Nakai	China, Korea	Royal Botanic Garden Edinburgh, 1987	3768	IV
20	*S*. *uratensis* Franch.	China	The Kórnik Arboretum,1956	0708	III
21	*S*. *veitchii* Hemsl.	China	Lack of data	0688	VIII
22	*S*. *wilsonii* Duthie	China	Hortus Botanicus Fominianus Universitatis Kioviensis, 2009	8408	XXII
23	*S*. *×billardii* Hérincq.		The Kórnik Arboretum, 1985	2848	XXIII
24	*S*. *×cinerea* Zabel ‘Grefsheim’		Lack of data, 1987	3010	II
25	*S*. *tomentosa* L.	United States, Canada	Poland, Bory Niemodlińskie, Tułowice, 2017, leg. B. Wiatrowska	PZNF	PZNF

PZNF–Herbarium of the Department of Forest Botany.

In this paper the taxonomic classification of the species from the genus *Spiraea* under analysis was adopted from Yü and Kuan [6], since the latest studies by Potter et al. [2, 3] and APG IV [4] do not provide systematic divisions within the studied genus. The taxa under examination represented all the four sections and eight (out of 10) series of the genus *Spiraea* [6]. Additionally, *S*. *×billardii* Hérincq was included—a hybrid of two species (*S*. *alba* Du Roi × *S*. *douglasii* Hook) from the *Spiraea* section, and *S*. *×cinerea* Zabel ’Grefsheim’, a hybrid of the species from the section *Chamaedryon* with a species from the section *Glomerati* Nakai.

The nomenclature of taxa was adopted from botanical studies characterizing flora of the corresponding regions of the world. For Europe it was Flora Europaea [10] and Illustrierte Flora von Mitteleuropa [11], for Central Asia and the Russian Far East—Flora of the U.S.S.R. [45], Deriewa i kustraniki SSSR [46] and Flora Sibiriae [47], while for the Far East—Flora of Japan [48] and Flora of China [49]. The names of hybrids were adopted from The European Garden Flora [50]. All plant names were compared and validated against the online databases [51, 52]. A list of the taxa analyzed together with their affiliation to particular sections and series is shown in Table 2.

**Table 2:** List of taxa analyzed with their affiliation to particular sections and series.

Section	Series	Taxa
*Spiraea*	-	*S*. *salicifolia* L., *S*. *douglasii* Hook., *S*. *alba* Du Roi,.S. *alba* var. *latifolia* (Aiton) Dippel; *S*. ×*billardii* Hérincq
*Calospira* K. Koch	**-**	*S*. *betulifolia* Pall., *S*. *splendens* É. N. Baumann ex K. Koch*
	*Canescentes* Yu	*S*. *uratensis* Franch., *S*. *trichocarpa* Nakai, *S*. *canescens* D. Don
	*Henryanae* Yu	*S*. *veitchii* Hemsl., *S*. *henryi* Hemsl., *S*. *wilsonii* Duthie
	*Japonicae* Yu	*S*. *japonica* L. f.
*Chamaedryon* Ser.	**-**	*S*. *cana* Waldst. & Kit, *S*. *nipponica* Maxim.
	*Chamaedryfoliae* Pojark.	*S*. *chamaedryfolia* L.
	*Mediae* Pojark.	*S*. *media* Schmidt
	*Trilobatae* Pojark.	*S*. *chinensis* Maxim., *S*. *elegans* Pojark., *S*. *pubescens* Turcz., *S*. *dasyantha* Bunge
*Glomerati* Nakai	*Hypericifoliae* Pojark.	*S*. *hypericifolia* L.
	*Prunifoliae* Yu	*S*. *thunbergii* Siebold ex Blume, *S*. ×*cinerea* Zabel ‘Grefsheim’

* species allocated to sections only**—**the section is not divided into series.

In accordance with the study by Wrońska-Pilarek et al. [53], each sample consisted of 30 randomly selected, mature and correctly formed pollen grains derived from a single individual (shrub). In total, 750 pollen grains were studied.

The pollen grains were prepared for light (LM) and scanning electron microscopy (SEM) using the standard acetolysis method described by Erdtman [54, 55]. An acetolysis mixture was prepared to consist of 9 parts acetic acid anhydride and one part concentrated sulfuric acid, while the acetolysis process lasted 2.5 minutes. The prepared material was divided into two parts: one part was immersed in an alcohol solution of glycerine (for LM) and the other in 96% ethyl alcohol (for SEM). Morphological observations were carried out using a light microscope (Biolar 2308, Nikon HFX-DX) and a scanning electron microscope (Jeol 7001TTLS). The pollen grains were measured in the equatorial view at a magnification of 640x.

Nine quantitative features of the pollen grains were analyzed, i.e., the length of the polar axis (P) and equatorial diameter (E), the length of the ectoaperture (Le), the thickness of the exine along the polar axis and equatorial diameter (Exp, Exe), as well as the P/E, Le/P, Exp/P and Exe/E ratios. Moreover, the following qualitative features were examined: the pollen outline and shape, and exine ornamentation (type, width and direction of grooves and striae, number and diameter of perforations).

The palynological terminology follows Punt et al. [56] and Halbritter et al. [57].

### Statistical analysis

The normality of the distributions for the studied traits (length of polar axis—P, equatorial diameter—E, length of ectoaperture—Le, exine thickness along the polar axis—Exp, exine thickness along the equatorial diameter—Exe, as well as the P/E, Le/P, Exp/P and Exe/E ratios) was tested using Shapiro-Wilk’s normality test [58]. A multivariate analysis of variance (MANOVA) was performed based on the following model using a MANOVA procedure in GenStat 18: **Y** = **XT**+**E**, where: **Y** is (*n*×*p*)–the dimensional matrix of observations, *n* is the total number of observations, *p* is the number of traits (in this study *p* = 9), **X** is (*n*×*k*)–the dimensional matrix of design, *k* is the number of species (in this study *k* = 25), **T** is (*k*×*p*)–the dimensional matrix of unknown effects, and **E** is (*n*×*p*)–the dimensional matrix of residuals. Afterward, one-way analyses of variance (ANOVA) were performed to verify the null-hypothesis of a lack of a species effect in terms of the values of the nine observed traits, independently for each trait, based on the following model: *y*_*ij*_ = *⎧*+*⎮*_*i*_+*∑*_*ij*_, where: *y*_*ij*_—*j*th observation of *i*th species, *⎧*—the grand mean, *⎮*_*i*_—the effect of *i*th species and *∑*_*ij*_—error observation. The minimal and maximal values of the traits as well as the arithmetic means and coefficients of variation (CV, in %) were calculated. Moreover, Fisher’s least significant differences (LSDs) were estimated at a significance level of *α* = 0.001. The relationships between the observed traits were estimated based on the species’ means using Pearson’s correlation coefficients. The results were also analyzed using multivariate methods. A canonical variate analysis was applied to present a multi-trait assessment of the similarity of the tested species in a lower number of dimensions with the least possible loss of information [59]. This made it possible to illustrate in the graphic form any variation in the species in terms of all the observed traits. The Mahalanobis distance was suggested as a measure of “polytrait” species similarity [60], the significance of which was verified using critical value D_*α*_ called “the least significant distance” [61]. Mahalanobis distances were calculated for the species. The differences between the analyzed species were verified by cluster analysis using the nearest neighbor method and Euclidean distances [62]. All the analyses were conducted using the GenStat 18 statistical software package.

## Results

### Morphological description of pollen

**Fig 1:**
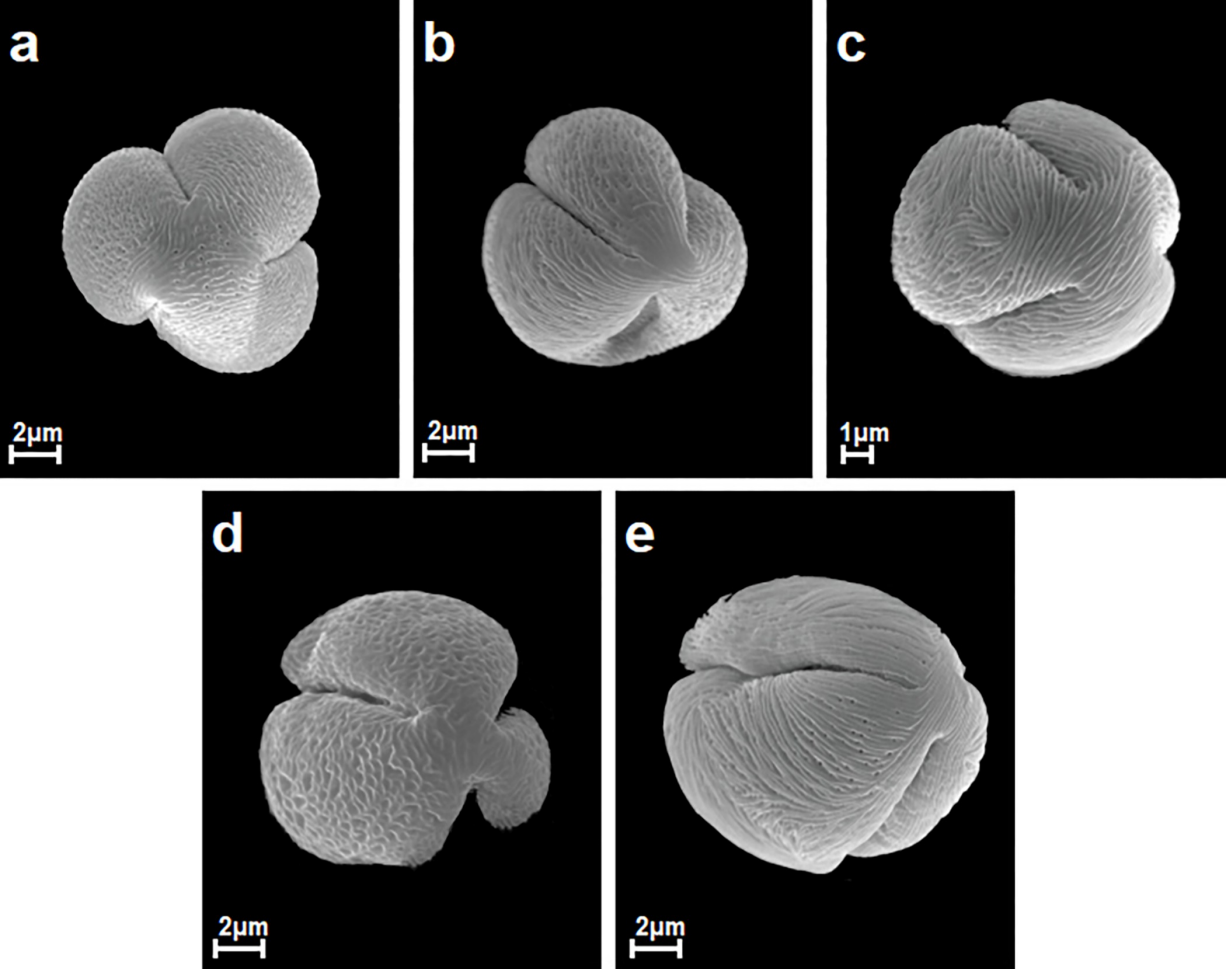
Pollen grains in polar view in *S*. *douglasii*, *S*. *elegans*, *S*. *thunbergii*, *S*. *japonica*, *S*. *uratensis*, A-E.

**Fig 2:**
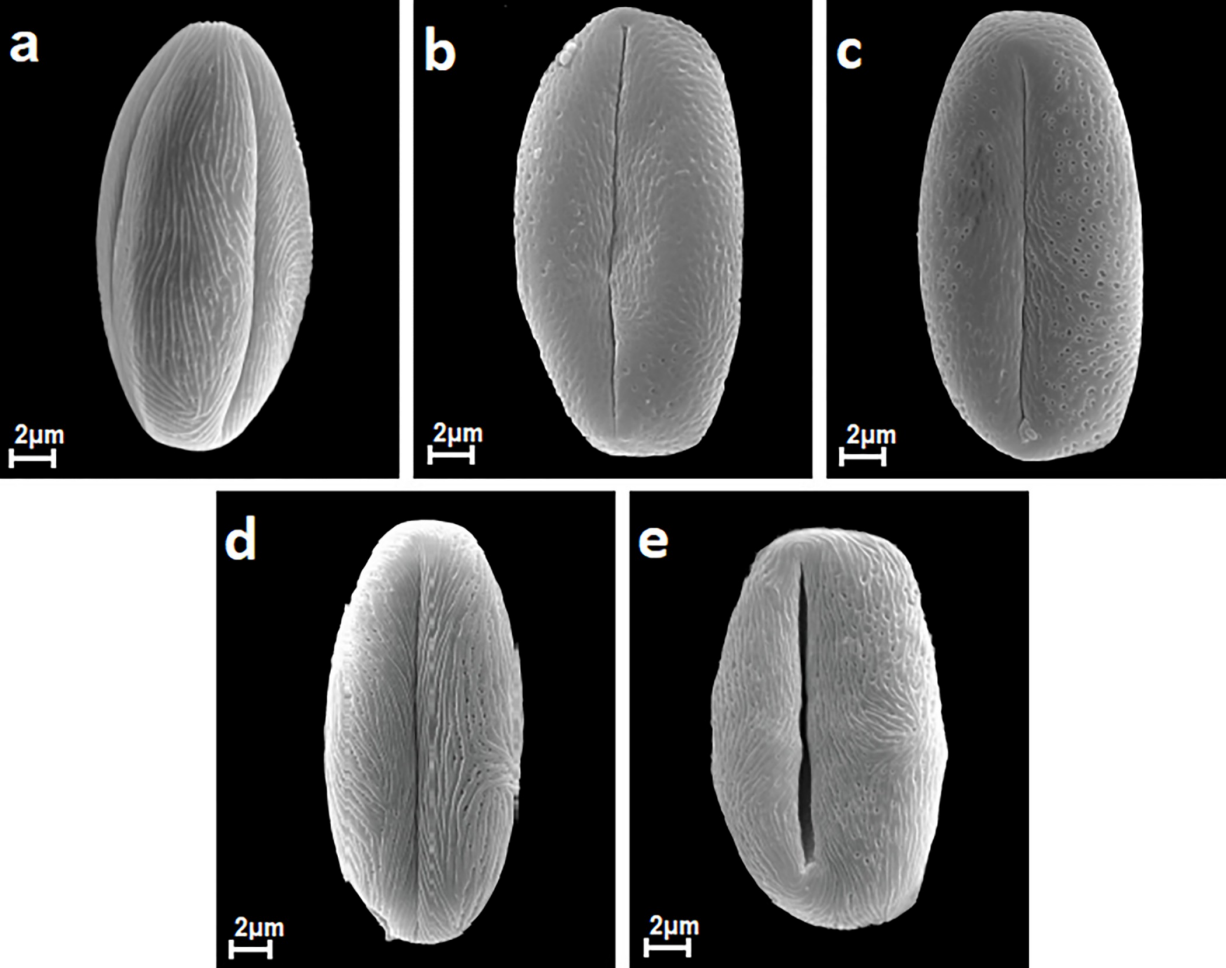
Pollen grains in equatorial view in *S*. *cana*, *S*. *henryi*, *S*. *salicifolia*, *S*. *trichocarpa*, *S*. *veitchii*, A-E.

**Fig 3:**
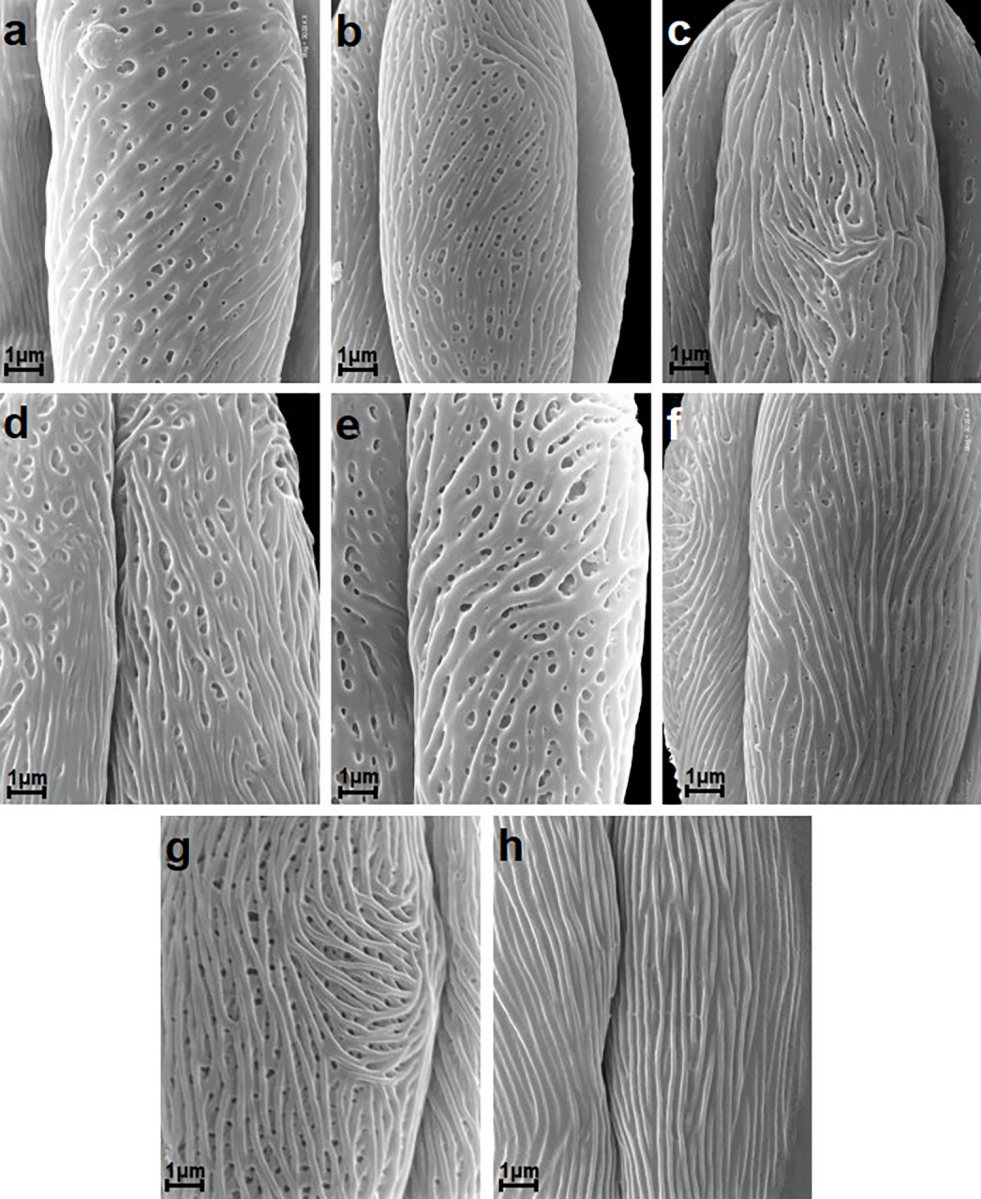
Striate exine ornamentation types according to Ueda and Tomita [63]; see Table 6. A-H. A, *S*. *nipponica*; B, *S*. *wilsonii*; C, *S*. *alba*; D, *S*. *betulifolia*; E, *S*. *×billardii*; F, *S*. *cana*; G, *S*. *chamaedryfolia*; H, *S*. *hypericifolia*.

**Fig 4:**
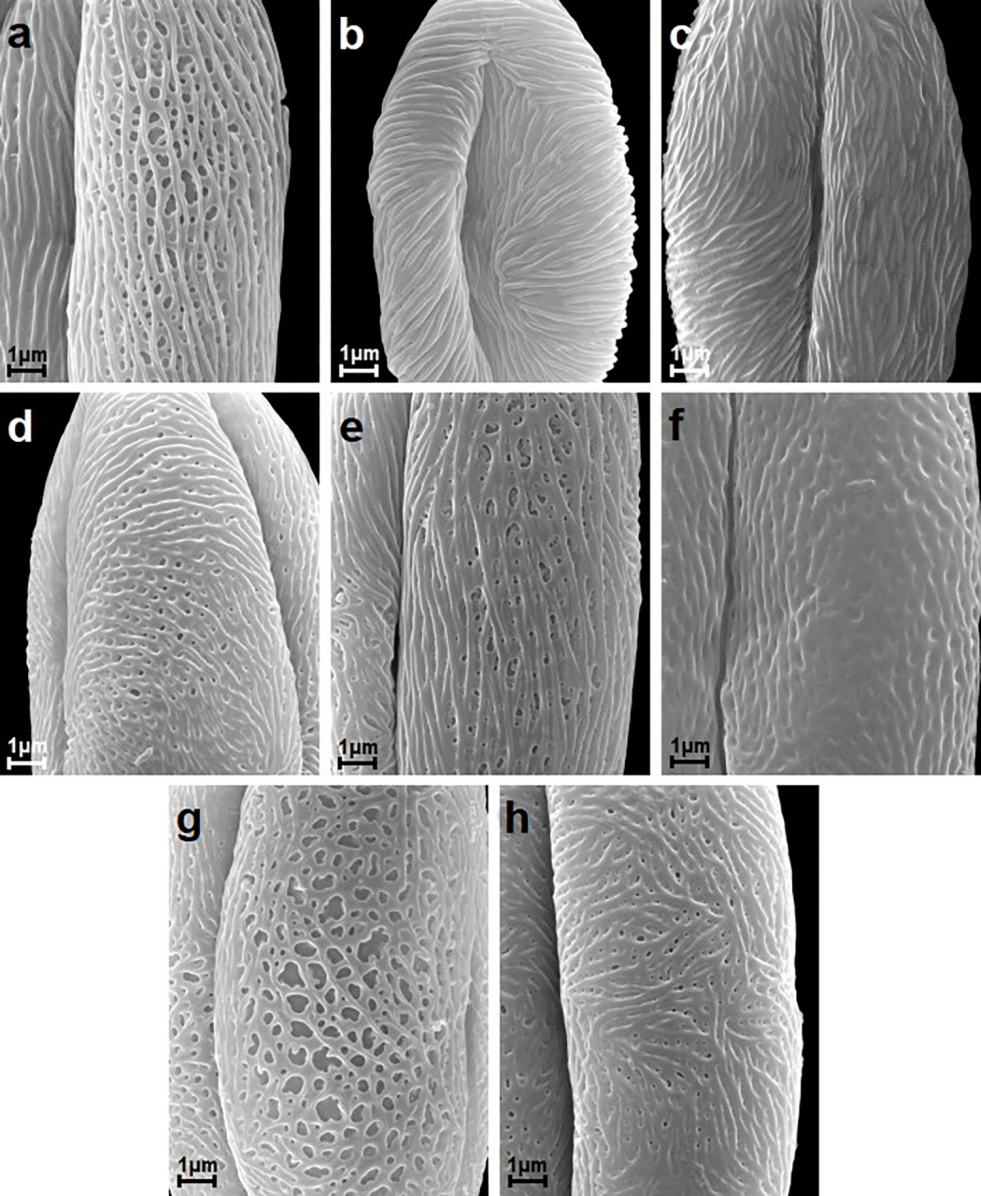
Striate exine ornamentation types according to Ueda and Tomita [63]; see Table 6. A-H. A, *S*. *chinensis*; B, *S*. *×cinerea* ‘Grefsheim’; C, *S*. *dasyantha*; D, *S*. *douglasii*; E, *S*. *elegans*; F, *S*. *henryi*; G, *S*. *japonica*; H, *S*. *alba* var. *latifolia*.

**Fig 5:**
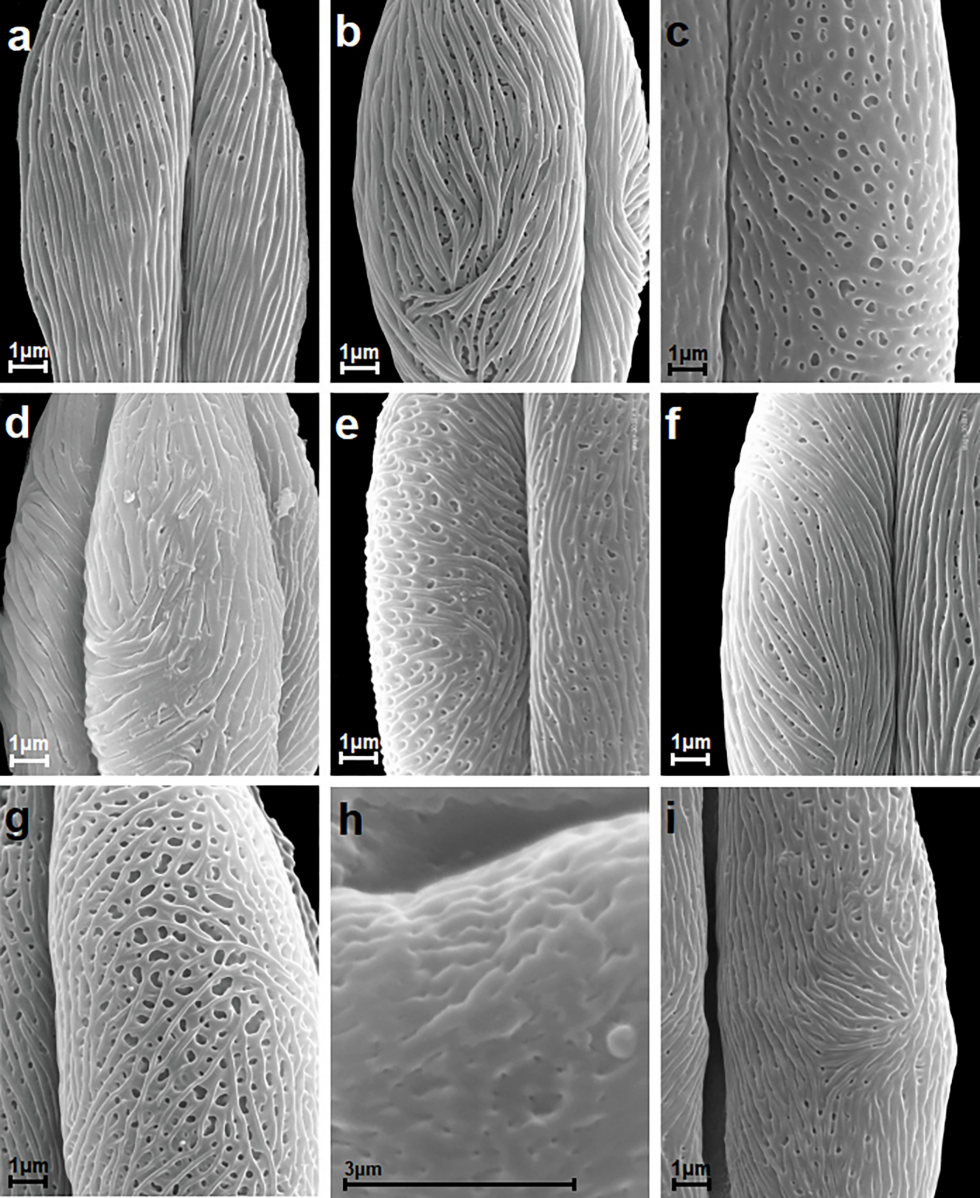
Striate exine ornamentation types according to Ueda and Tomita [63]; see Table 6. A-I. A, *S*. *media*; B, *S*. *pubescens*; C, *S*. *salicifolia*; D, *S*. *splendens*; E, *S*. *thunbergii*; F, *S*. *trichocarpa*; G, *S*. *uratensis*; H, *S*. *veitchii*; I, *S*. *tomentosa*.

A description of pollen grain morphology of the *Spiraea* species under analysis is given below and illustrated in the SEM photographs (Figs 1–5). The morphological observations for the quantitative features are summarized in Tables 3–5 and S1 Table.

**Table 3:** Minimal, maximal and mean values as well as coefficients of variation (CV) for length of polar axis (P), equatorial diameter (E) and length of ectoaperture (Le) in studied taxa.

Trait	P				E				Le			
Species	Mean		Min-Max	s.d.	Mean		Min-Max	s.d.	Mean		Min-Max	s.d.
*S*. *alba*	16.33	def	14–20	1.06	16.07	cd	14–20	1.11	15.27	c	12–20	1.70
*S*. *alba* var. *latifolia*	18.27	ab	16–20	1.02	17.87	b	16–20	1.04	17.53	ab	14–20	1.46
*S*. *betulifolia*	18.67	a	16–20	1.32	19.27	a	16–20	1.23	16.67	b	14–20	1.61
*S*. *cana*	16	defg	14–18	1.17	14.87	fghij	12–18	1.72	14.53	cde	12–18	1.57
*S*. *chamaedryfolia*	18.6	a	16–20	1.07	19.73	a	18–20	0.69	17.67	ab	16–20	1.40
*S*. *chinensis*	15.8	defg	14–18	1.10	15.6	cdef	14–18	0.97	14.93	cd	12–18	1.72
*S*. *dasyantha*	14.4	i	14–16	0.81	14.07	ij	12–16	0.64	13.8	def	12–16	1.10
*S*. *douglasii*	15.4	fgh	14–18	1.50	14.47	ghij	12–16	1.25	14.47	cdef	12–18	1.80
*S*. *elegans*	15.6	efg	14–16	0.81	15.73	cdef	14–16	0.69	15	cd	12–16	1.15
*S*. *henryi*	15.2	ghi	14–16	1.00	14.93	efghij	14–16	1.02	14.2	cdef	12–16	1.42
*S*. *hypericifolia*	18.27	ab	16–20	1.26	19.13	a	16–22	1.36	17.5	ab	16–20	1.55
*S*. *japonica*	15.6	efg	14–18	0.97	14.87	fghij	14–16	1.01	14.07	cdef	12–16	1.44
*S*. *media*	15.67	defg	14–18	1.18	15.8	cdef	14–18	1.10	14.67	cde	12–18	1.69
*S*. *nipponica*	16.6	cd	14–22	1.40	15.67	cdef	14–20	1.58	14.93	cd	12–22	1.80
*S*. *pubescens*	14.53	hi	12–16	1.04	14	j	12–16	1.05	14	cdef	12–16	1.39
*S*. *salicifolia*	19	a	18–20	1.02	19.33	a	16–20	1.09	18.27	a	16–20	1.26
*S*. *splendens*	16.47	de	14–20	1.25	15.2	defgh	14–18	1.13	15.2	c	14–18	1.13
*S*. *thunbergii*	15.4	fgh	14–18	1.07	15	efghi	12–18	1.26	13.6	ef	12–16	1.52
*S*. *tomentosa*	16.33	def	14–20	1.67	16.2	c	14–18	1.32	13.2	f	10–18	1.71
*S*. *trichocarpa*	15.8	defg	14–18	0.96	15.4	cdefg	14–16	0.93	14.6	cde	12–16	1.30
*S*. *uratensis*	17.53	bc	16–20	1.01	15.4	cdefg	14–16	0.93	17	ab	14–20	1.37
*S*. *veitchii*	16.53	de	14–20	1.28	15.87	cde	14–20	1.38	14.67	cde	12–20	2.12
*S*. *wilsonii*	15.73	defg	14–18	1.02	14.93	efghij	12–16	1.14	14.13	cdef	12–16	1.38
*S*. *×billardii*	18.13	ab	14–20	1.38	16.27	c	14–20	1.64	17.13	ab	14–20	1.87
*S*. *×cinerea*	15.67	defg	14–18	1.18	14.27	hij	12–16	1.02	14.67	cde	12–18	1.69
LSD0.001	0.989				0.992				1.317			
ANOVA F	39.57				64.52				26.73			
F value	<0.001				<0.001				<0.001			

**Table 4:** Minimal, maximal and mean values as well as coefficients of variation (CV) for exine thickness along the polar axis (Exp), exine thickness along the equatorial diameter (Exe) and P/E of analyzed species.

Trait	Exp				Exe				P/E			
Species	Mean		Min-Max	s.d.	Mean		Min-Max	s.d.	Mean		Min-Max	s.d.
*S*. *alba*	0.98	defg	0.2–1.6	0.28	0.98	cde	0.2–1.6	0.28	1.019	defghi	0.88–1.14	0.066
*S*. *alba var*. *latifolia*	0.787	gh	0.2–1.6	0.34	0.793	ef	0.2–1.6	0.38	1.024	defghi	0.90–1.13	0.062
*S*. *betulifolia*	0.653	h	0.2–1	0.23	0.653	f	0.2–1	0.23	0.972	hij	0.80–1.25	0.086
*S*. *cana*	1.253	bcd	0.6–1.6	0.34	1.253	abc	0.6–1.6	0.34	1.089	abcd	0.88–1.33	0.134
*S*. *chamaedryfolia*	0.913	efgh	0.2–1.6	0.43	0.913	def	0.2–1.6	0.43	0.943	j	0.80–1.00	0.057
*S*. *chinensis*	0.68	h	0.2–1	0.19	0.68	f	0.2–1	0.19	1.014	efghij	0.88–1.14	0.061
*S*. *dasyantha*	0.793	fgh	0.6–1.6	0.25	0.793	ef	0.6–1.6	0.25	1.026	defghi	0.88–1.17	0.075
*S*. *douglasii*	0.853	efgh	0.6–1.6	0.28	0.853	def	0.6–1.6	0.28	1.067	bcdef	0.88–1.29	0.089
*S*. *elegans*	1.053	bcdefg	0.6–1.6	0.31	1.053	abcde	0.6–1.6	0.31	0.993	ghij	0.88–1.14	0.059
*S*. *henryi*	0.973	defg	0.6–2	0.4	0.973	cde	0.6–2	0.4	1.02	defghi	0.88–1.14	0.07
*S*. *hypericifolia*	0.873	efgh	0.2–1.6	0.36	0.873	def	0.2–1.6	0.36	0.958	ij	0.80–1.13	0.076
*S*. *japonica*	1.013	cdefg	0.6–1.6	0.28	1.013	bcde	0.6–1.6	0.28	1.052	bcdefg	0.88–1.14	0.077
*S*. *media*	0.987	cdefg	0.6–1.6	0.3	0.987	bcde	0.6–1.6	0.3	0.996	fghij	0.78–1.29	0.096
*S*. *nipponica*	1.313	b	0.6–2	0.42	1.313	a	0.6–2	0.42	1.066	bcdef	0.89–1.29	0.098
*S*. *pubescens*	0.847	efgh	0.2–1.6	0.29	0.847	def	0.2–1.6	0.29	1.041	cdefgh	1.00–1.33	0.082
*S*. *salicifolia*	0.673	h	0.2–1.6	0.28	0.66	f	0.2–1.6	0.27	0.986	ghij	0.90–1.13	0.074
*S*. *splendens*	1.053	bcdefg	0.6–2	0.36	1.053	abcde	0.6–2	0.36	1.086	abcde	0.88–1.14	0.075
*S*. *thunbergii*	1.087	bcde	0.6–1.6	0.38	1.087	abcd	0.6–1.6	0.38	1.03	cdefghi	0.88–1.17	0.076
*S*. *tomentosa*	1.667	a	02-sty	0.48	1.273	ab	0.6–2	0.49	1.016	efghi	0.78–1.29	0.138
*S*. *trichocarpa*	1.08	bcdef	0.6–1.6	0.29	1.08	abcde	0.6–1.6	0.29	1.028	cdefghi	0.88–1.14	0.066
*S*. *uratensis*	1.22	bcd	0.6–1.6	0.36	1.22	abc	0.6–1.6	0.36	1.142	a	1.00–1.43	0.085
*S*. *veitchii*	1.02	cdefg	0.6–1.6	0.37	1.02	bcde	0.6–1.6	0.37	1.046	cdefg	0.88–1.14	0.086
*S*. *wilsonii*	0.907	efgh	0.6–1.6	0.34	0.893	def	0.6–1.6	0.34	1.057	bcdefg	0.88–1.17	0.079
*S*. *×billardii*	0.84	efgh	0.6–1.6	0.28	0.84	def	0.6–1.6	0.28	1.121	ab	1.00–1.29	0.095
*S*. *×cinerea*	1.273	bc	0.6–2	0.42	1.273	ab	0.6–2	0.42	1.1	abc	1.00–1.29	0.077
LSD0.001	0.288				0.29				0.072			
ANOVA F	13.97				9.7				10.13			
F value	<0.001				<0.001				<0.001			

**Table 5:** Minimal, maximal and mean values as well as coefficients of variation (CV) for Le/P, Exp/P and Exe/E of analyzed species.

Trait	Le/P				Exp/P				Exe/E			
Species	Mean		Min-Max	s.d.	Mean		Min-Max	s.d.	Mean		Min-Max	s.d.
*S*. *alba*	0.935	abcdef	0.75–1	0.083	0.06	def	0.013–0.100	0.018	0.061	cdefg	0.013–0.100	0.018
*S*. *alba var*. *latifolia*	0.961	abc	0.70–1	0.07	0.043	fg	0.011–0.089	0.019	0.045	fgh	0.011–0.089	0.022
*S*. *betulifolia*	0.894	ef	0.78–1	0.067	0.035	g	0.010–0.063	0.013	0.034	h	0.010–0.063	0.012
*S*. *cana*	0.908	abcdef	0.75–1	0.066	0.079	bc	0.033–0.114	0.022	0.085	ab	0.038–0.133	0.025
*S*. *chamaedryfolia*	0.95	abcde	0.80–1	0.06	0.049	efg	0.011–0.089	0.023	0.047	efgh	0.010–0.089	0.022
*S*. *chinensis*	0.945	abcdef	0.75–1	0.079	0.043	fg	0.013–0.063	0.012	0.044	gh	0.013–0.063	0.012
*S*. *dasyantha*	0.959	abcd	0.86–1	0.064	0.055	def	0.038–0.100	0.016	0.056	defg	0.043–0.114	0.017
*S*. *douglasii*	0.939	abcdef	0.86–1	0.067	0.056	def	0.033–0.114	0.019	0.059	defg	0.038–0.114	0.019
*S*. *elegans*	0.962	ab	0.86–1	0.059	0.068	bcd	0.038–0.114	0.02	0.067	bcd	0.038–0.100	0.02
*S*. *henryi*	0.935	abcdef	0.75–1	0.081	0.064	bcde	0.038–0.125	0.025	0.066	bcde	0.038–0.143	0.029
*S*. *hypericifolia*	0.959	abcd	0.80–1	0.064	0.048	efg	0.010–0.089	0.019	0.046	fgh	0.010–0.089	0.019
*S*. *japonica*	0.903	bcdef	0.75–1	0.091	0.065	bcde	0.033–0.114	0.019	0.068	bcd	0.038–0.114	0.019
*S*. *media*	0.935	abcdef	0.86–1	0.067	0.063	cde	0.033–0.100	0.018	0.063	cdefg	0.033–0.114	0.019
*S*. *nipponica*	0.899	def	0.78–1	0.062	0.079	bc	0.043–0.125	0.024	0.084	ab	0.043–0.125	0.026
*S*. *pubescens*	0.963	ab	0.86–1	0.062	0.058	def	0.013–0.100	0.019	0.061	cdefg	0.013–0.114	0.021
*S*. *salicifolia*	0.963	ab	0.80–1	0.062	0.036	g	0.010–0.089	0.015	0.034	h	0.010–0.089	0.015
*S*. *splendens*	0.926	abcdef	0.78–1	0.076	0.064	bcde	0.033–0.114	0.022	0.07	abcd	0.038–0.125	0.024
*S*. *thunbergii*	0.884	f	0.75–1	0.082	0.07	bcd	0.038–0.114	0.024	0.072	abcd	0.038–0.114	0.023
*S*. *tomentosa*	0.81	g	0.63–0.9	0.085	0.103	a	0.056–0.143	0.032	0.079	abc	0.038–0.143	0.03
*S*. *trichocarpa*	0.924	abcdef	0.86–1	0.063	0.069	bcd	0.033–0.100	0.018	0.07	abcd	0.038–0.114	0.019
*S*. *uratensis*	0.969	a	0.88–1	0.052	0.07	bcd	0.033–0.100	0.021	0.079	abc	0.038–0.114	0.022
*S*. *veitchii*	0.886	f	0.75–1	0.095	0.062	cde	0.033–0.114	0.022	0.064	cdef	0.038–0.114	0.022
*S*. *wilsonii*	0.9	cdef	0.75–1	0.083	0.058	def	0.033–0.114	0.023	0.061	cdefg	0.038–0.114	0.025
*S*. *×billardii*	0.945	abcdef	0.78–1	0.07	0.047	efg	0.030–0.089	0.017	0.053	defgh	0.030–0.114	0.022
*S*. *×cinerea*	0.935	abcdef	0.86–1	0.066	0.082	b	0.038–0.125	0.028	0.089	a	0.043–0.143	0.028
LSD0.001	0.061				0.018				0.019			
ANOVA F	7.52				16.31				14.44			
F value	<0.001				<0.001				<0.001			

The pollen grains of the studied species occur as radially symmetric, tricolporate, isopolar monads (Figs 1 and 2).

According to Erdtman’s pollen size classification [54], all of the analyzed pollen grains were small (10.1–25 μm). The length of the polar axis (P) was 16.46 (12.00–22.00) μm. The smallest P (12.00 μm) were found in *S*. *pubescens*, while the largest ones (22.00 μm) in *S*. *nipponica*. The average value of this feature ranged from 14.00 to 19.73 μm (S1 Fig).

The mean length of the equatorial diameter (E) was 16.00 (12.00–22.00) μm. The smallest value of this feature (12.00 μm) was found in *S*. *cana*, *S*. *douglasii*, *S*. *pubescens*, *S*. *thunbergii* and *S*. ×*cinerea* ‘Grefsheim’, whereas the largest ones (22.00 μm) only in *S*. *hypericifolia*. The average value of this trait ranged between 13.20 and 18.27 μm (S1 Fig).

The outline in the polar view was mostly circular or more rarely elliptic, while in the equatorial view it was circular or elliptic (Figs 1 and 2).

The mean P/E ratio was 1.04, ranging from 0.78 in *S*. *media* to 1.43 in *S*. *uratensis*. The average value of this ratio was very similar and ranged from 0.81 to 0.97. The pollen shape was most frequently spheroidal (398 pollen grains– 53.1%) and prolate-spheroidal (215–28.7%), rarely oblate-spheroidal (61–8.1%), suboblate (37–4.9%), subprolate (37–4.9%), and very rarely prolate (2–0.3%).

The mean exine thickness was 0.98 μm (Exe)– 0.99 μm (Exp) (within the range of 0.20–2.00 μm). The relative exine thickness (Exp/P and Exe/E ratios) averaged 0.09 and 0.06 (Exp/P–from 0.01 to 0.90 and Exe/E–from 0.01 to 0.14), respectively.

Exine ornamentation was usually striate-perforate, rarely striate and in one species striate-reticulate (Figs 3–5). The striae and grooves usually ran parallel to the polar axis, but they also frequently formed loops. They were straight or forked and of varying length and width (Figs 3–5). Either the grooves were narrower than the striae, or the widths of the grooves and striae were similar and averaged from 0.1 to 0.3 μm. Circular or elliptic perforations of different diameters–small to large (from 0.1 to 1 μm)–were found at the bottom of the striae (Figs 3–5).

Pollen of the individual *Spiraea* species under analysis was classified according to the striate exine ornamentation classification proposed by Ueda and Tomita [63] and Ueda [24] into five types (I-V) and six subtypes (I A,B, II A,B and III A,B). Ueda and Tomita [63] distinguished six types (I-VI) and six subtypes (I-III, each A and B). The above classification was applied, because it is based on the analysis of striate exine ornamentation in pollen of many species and genera from the family Rosaceae, and thus it best differentiates this type of ornamentation in the studied genus *Spiraea*. Type VI was not found in this study (Figs 3–5, Table 6). The greatest number of species (9) belonged to the IIA subtype, which was characterized by fairly distinct striae, narrow grooves and frequently numerous perforations of different diameters. Subtypes IA, IIIA, IIIB, IV and V were represented by two or three species, while type IB, by only one species (Figs 3–5, Table 6). In *S*. *henryi* only striate-reticulate exine ornamentation was found.

**Table 6:** Exine ornamentation types and subtypes of *Spiraea* species studied (according to the classification of Ueda and Tomita [63]).

Striate exine ornamentation type or subtype	Species
IA	*S*. *dasyantha*, *S*. *hypericifolia*, *S*. *media*
IB	*S*. *cana*
IIA	*S*. *alba* var. *latifolia*, *S*. *chamaedryfolia*, *S*. *douglasii*, *S*. *pubescens*, *S*. *thunbergii*, *S*. *tomentosa*, *S*. *trichocarpa*, *S*. *veitchii*, *S*. *wilsonii*
IIIA	*S*. *alba*, *S*. ***×****cinerea* ’Grefsheim’, *S*. *splendens*
IIIB	*S*. *betulifolia*, *S*. ***×****billardii*, *S*. *elegans*
IV	*S*. *chinensis*, *S*. *japonica*, *S*. *uratensis*
V	*S*. *nipponica*, *S*. *salicifolia*
striate-reticulate	*S*. *henryi*

Pollen grains usually possess three apertures–the colpi. These were distributed symmetrically, elongated, and narrowed toward the poles, with granular aperture membranes. The mean length of the colpi ranged between 10.00 and 22.00 μm, with an average of 15.27 μm. The length of the colpi typically constituted 93% of the polar axis length (P) and ranged from 63 to 100%. Their width was variable and usually the greatest in the equatorial region. The endoapertures were typically located in the middle of the colpi, less frequently asymmetrically, usually singly. They were circular or elliptic in outline with irregular margins.

Key to identification of the *Spiraea* species under analysis based on pollen features.

1 Exine ornamentation striate-reticulate…… …… …… …… …… …… …… …… …… …… …… …… .*S*. *henryi*

1*Exine ornamentation striate…………………………………………………………………2

2 Exine ornamentation striate without perforations…… …… …… …… …… …… …… …… …… …… ……3

2* Exine ornamentation striate with perforations…… …… …… …… …… …… …… …… …… …… …… …6

3 Exine ornamentation subtype IA (grooves distinct and of medium width, striae narrow; perforations few or absent, small)……………………………………………………………4

3* Exine ornamentation type IIIA (grooves distinct and wide, striae narrow; perforations few or absent, small)…….………………………………………………………………………5

4. Striae wavy……………………………………………………………………*S*. *dasyantha*

4* Striae straight……………………………………………………… …… …… … *S*. *hypericifolia*

5. Striae run across the P axis………………………………………………*S*. *cinerea* ’Grefsheim’

5* Striae extending along the P axis…………………………………….………….*S*. *splendens*

6 Exine ornamentation type I……….………………………………….………………………7

6* Other type of exine ornamentation………………………………………………………….8

7 Exine ornamentation subtype IA (grooves distinct and of medium width, striae narrow; perforations few or absent, small)………………………………………………………*S*. *media*

7* Exine ornamentation subtype IB (grooves distinct and of medium width, striae wide; perforations few or absent, small)………………………………………………………*…S*. *cana*

8 Exine ornamentation type II……….……………….………………………………………9

8* Other types of exine ornamentation………………….…………………………………….11

9 Exine ornamentation subtype IIA (grooves very distinct and of medium width, striae narrow; perforations numerous, with different diameters (mostly small)……….…………… …… …….10

10 Range of E narrow (14–18), range of P/E ratio large (0.778–1.286), exine thick (Exp—1.2733)……………………………………………………………………………*S*. *tomentosa*

10* Other features……………………………………….………………… .*S*. *alba* var. *latifolia*, *S*. *chamaedryfolia*, *S*. *douglasii*, *S*. *pubescens*, *S*. *thunbergii*, *S*. *trichocarpa*, *S*. *veitchii*, *S*. *wilsonii*

11 Exine ornamentation type III…….….……………………………………………….….12

11* Other type of exine ornamentation…….…………….………………………………….13

12 Exine ornamentation type IIIA (grooves distinct and wide, striae narrow; perforations few or absent, small)……………………………………….…………………………………*S*. *alba*

12* Exine ornamentation type IIIB (grooves distinct, flattened and wide, striae narrow; perforations numerous and mostly very large)………………………… …………….*S*. *betulifolia*, *S*. ***×****billardii*, *S*. *elegans*

13 Exine ornamentation type IV (grooves distinct and of medium width, flat, striae very wide; perforations very numerous and most extremely large)……………………………*S*. *chinensis*, *S*. *japonica*, *S*. *uratensis*

13* Exine ornamentation type V (grooves indistinct, flat and blurred, striae wide; perforations numerous and usually large)…………………………………………*S*. *nipponica*, *S*. *salicifolia*

### Intrageneric and interspecific variability of pollen grains

**Fig 6:**
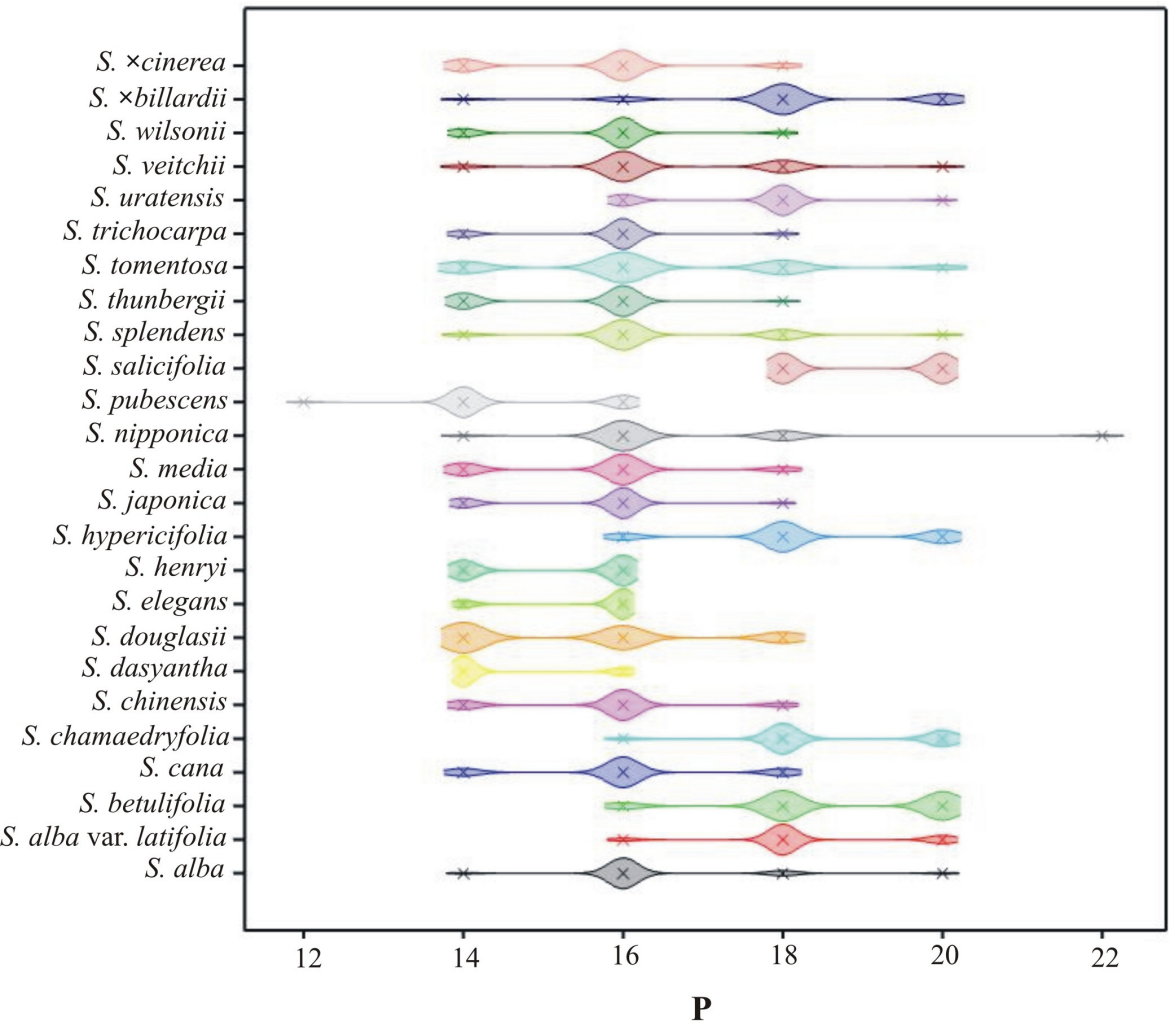
The density plot of P by *Spiraea* taxa.

**Fig 7:**
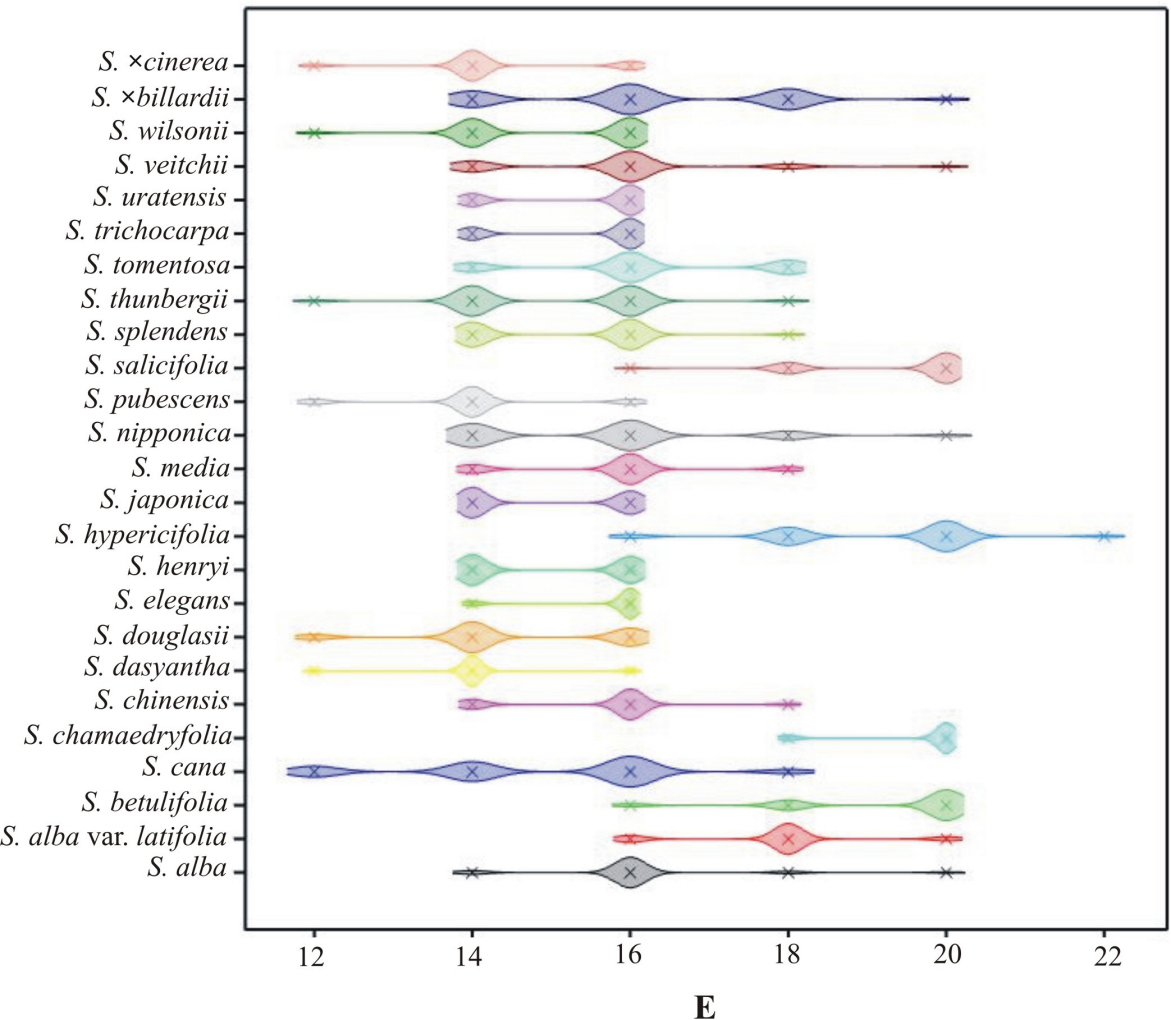
The density plot of E by *Spiraea* taxa.

**Fig 8:**
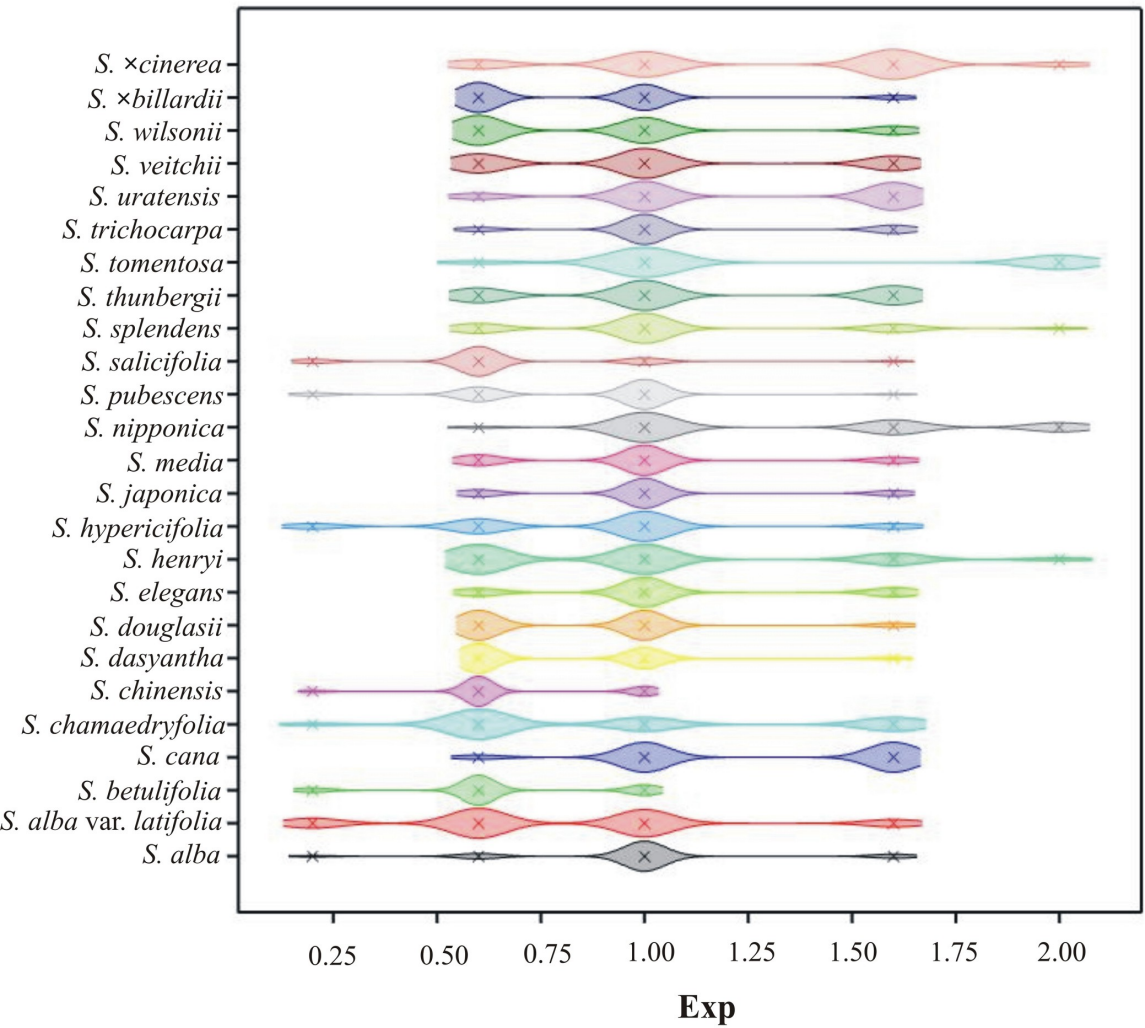
The density plot of Exp by *Spiraea* taxa.

**Fig 9:**
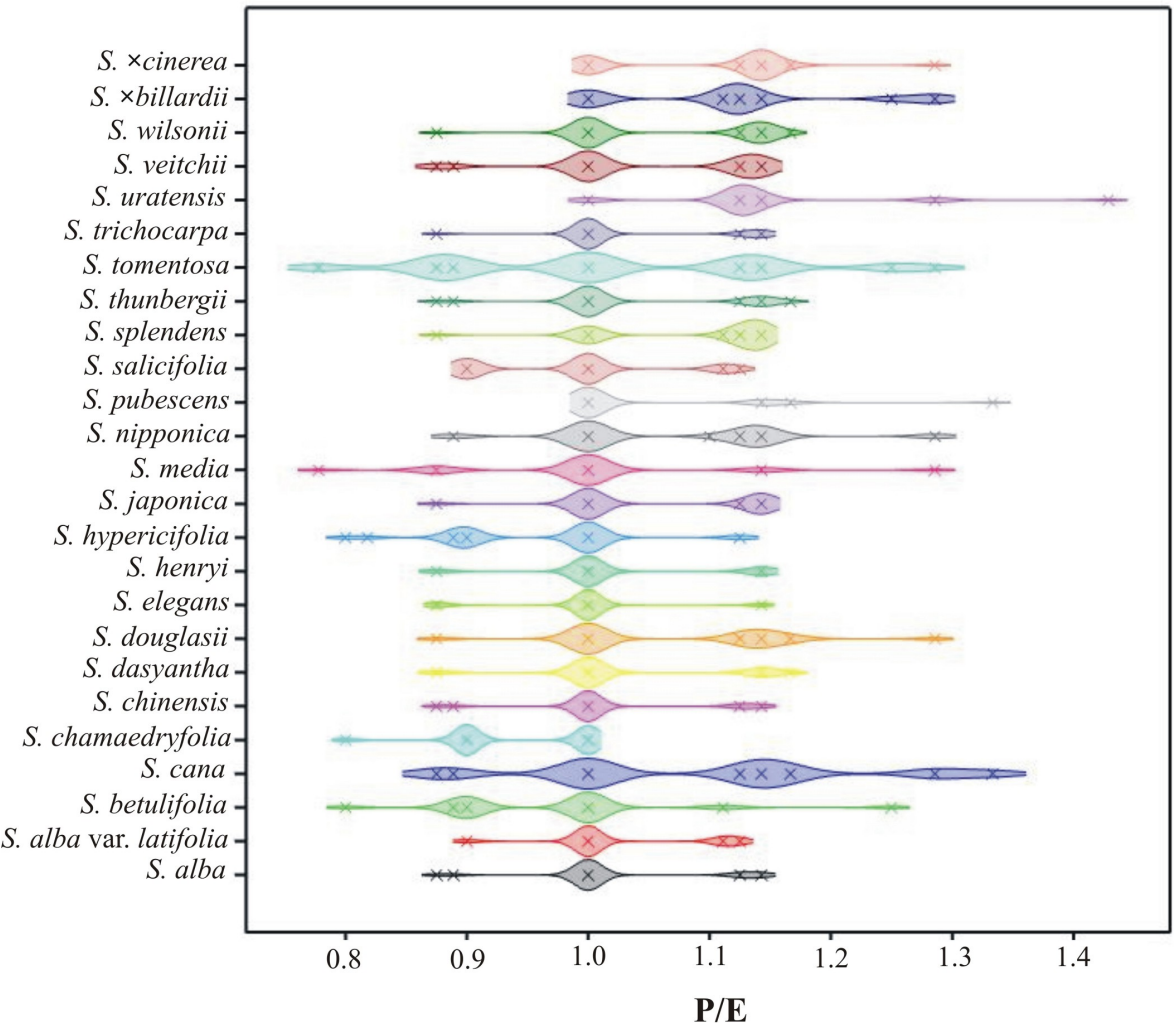
The density plot of P/E by *Spiraea* taxa.

The results of the MANOVA performed indicated that all the samples were significantly different with regard to all of the nine quantitative traits (Wilk’s λ = 0.05895; *F*_24,725_ = 11.20; *P*<0.0001). The results of ANOVA indicated that the main effects of the species were significant for all the nine observed traits (Tables 3–5 and S1 Table). The mean values and standard deviations for the observed traits indicated high variability among the tested samples, for which significant differences were found in terms of all the analyzed morphological traits (Tables 3–5 and S1 Table) (Figs 6–9).

The interspecific variability of the *Spiraea* pollen grains was examined based on nine selected quantitative features. Statistical analysis of the studied features indicated high variability among the tested species. The most variable biometric traits were E and P (Table 3), while lower variability was observed in the Le/P ratio and Exe (Tables 4 and 5). A ranking of variability of the observed traits is as follows: E > P > Le > Exp/P > Exe/E > Exp > P/E > Exe > Le/P. The most variable species for all the nine traits jointly were *S*. *×billardii*, *S*. *veitchii*, *S*. *nipponica* and *S*. *cana*, while those with lower variability were *S*. *dasyantha*, *S*. *elegans*, *S*. *trichocarpa* and *S*. *chamaedryfolia*.

**Fig 10:**
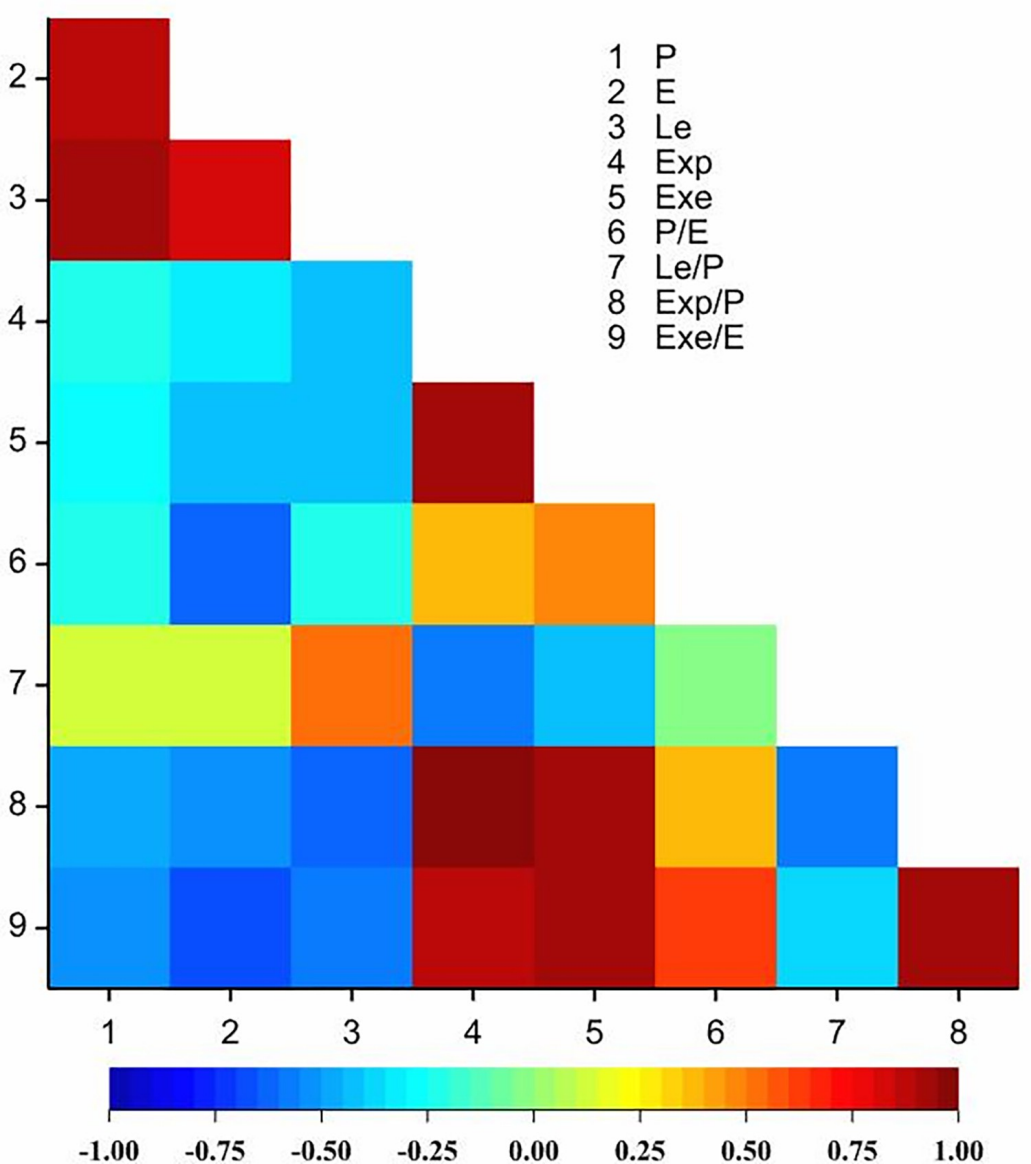
Heatmap for Pearson’s correlation coefficients between observed traits for *Spiraea* taxa (r_0.05_ = 0.38; r_0.01_ = 0.49; r_0.001_ = 0.60).

The correlation analysis performed indicated statistically significant correlation coefficients for 25 out of 36 coefficients (Table 7, Fig 10). Twelve out of 25 significantly correlated pairs of traits were characterized by positive correlation coefficients. Negative correlation coefficients were observed between P and Exp/P (-0.486), P and Exe/P (-0.530), E and Exe (-0.376), E and P/E (-0.629), E and Exp/P (-0.524), E and Exe/E (-0.666), Le and Exp (-0.432), Le and Exe (-0.396), Le and Exp/P (-0.645), Le and Exe/E (-0.600), Exp and Le/P (-0.592), Exe and Le/P (-0.430), and Le/P and Exp/P (-0.586). In the case of eleven pairs of traits, no significant correlation was established (Table 7, Fig 10).

**Table 7:** Correlation coefficients between observed traits.

Trait	P	E	Le	Exp	Exe	P/E	Le/P	Exp/P	Exe/E
P	1								
E	0.898***	1							
Le	0.914***	0.815***	1						
Exp	-0.245	-0.347	-0.444*	1					
Exe	-0.285	-0.426*	-0.409*	0.946***	1				
P/E	-0.241	-0.641***	-0.212	0.365	0.471*	1			
Le/P	0.132	0.109	0.522**	-0.587**	-0.422*	-0.025	1		
Exp/P	-0.481*	-0.542**	-0.644***	0.966***	0.922***	0.382	-0.579**	1	
Exe/E	-0.527**	-0.683***	-0.596**	0.870***	0.948***	0.614**	-0.367	0.918***	1

* P<0.05

** P<0.01

*** P<0.001

**Fig 11:**
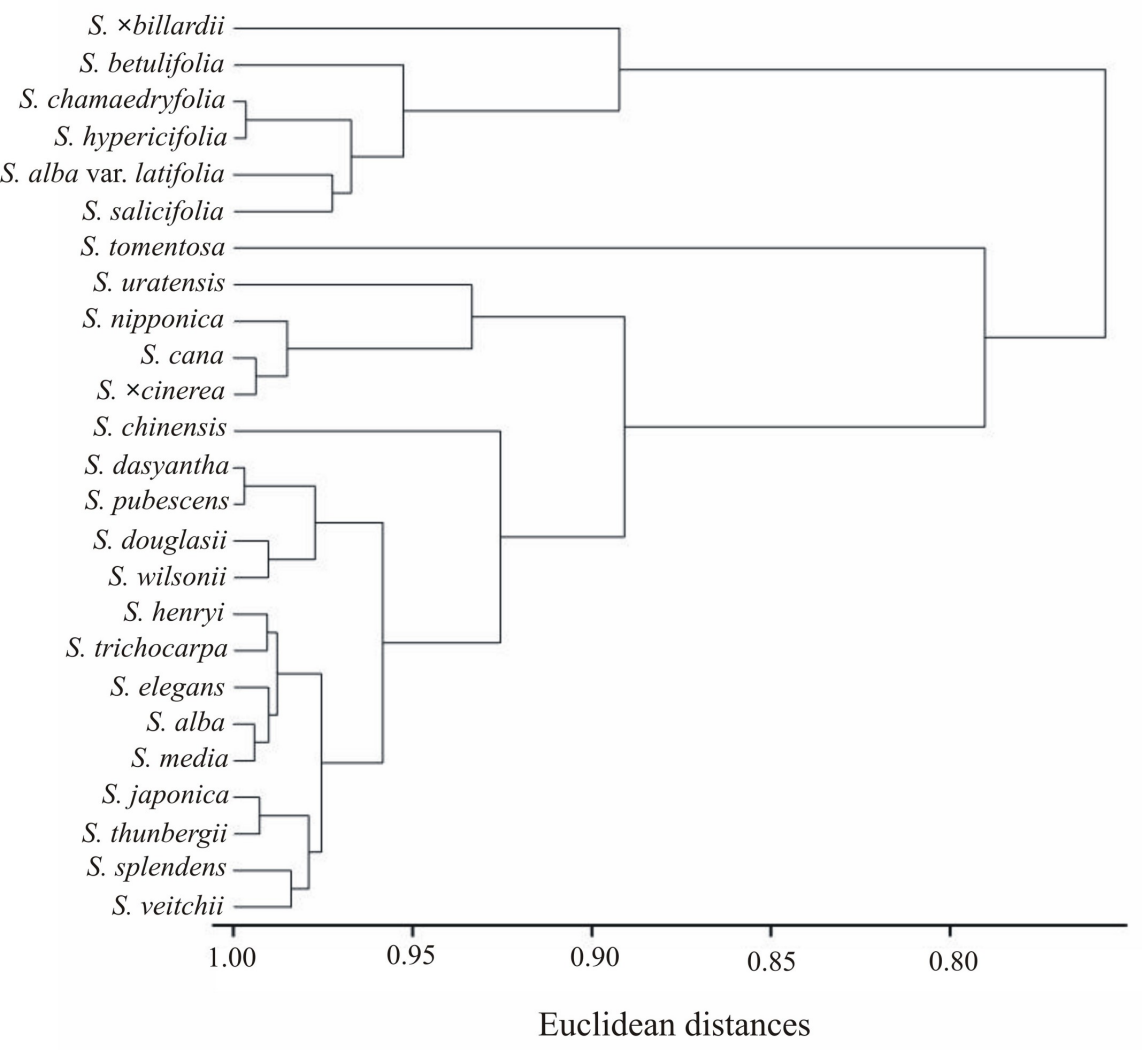
Clustering (Neighbor Joining Method) of *Spiraea* species based on all nine morphological traits.

**Fig 12:**
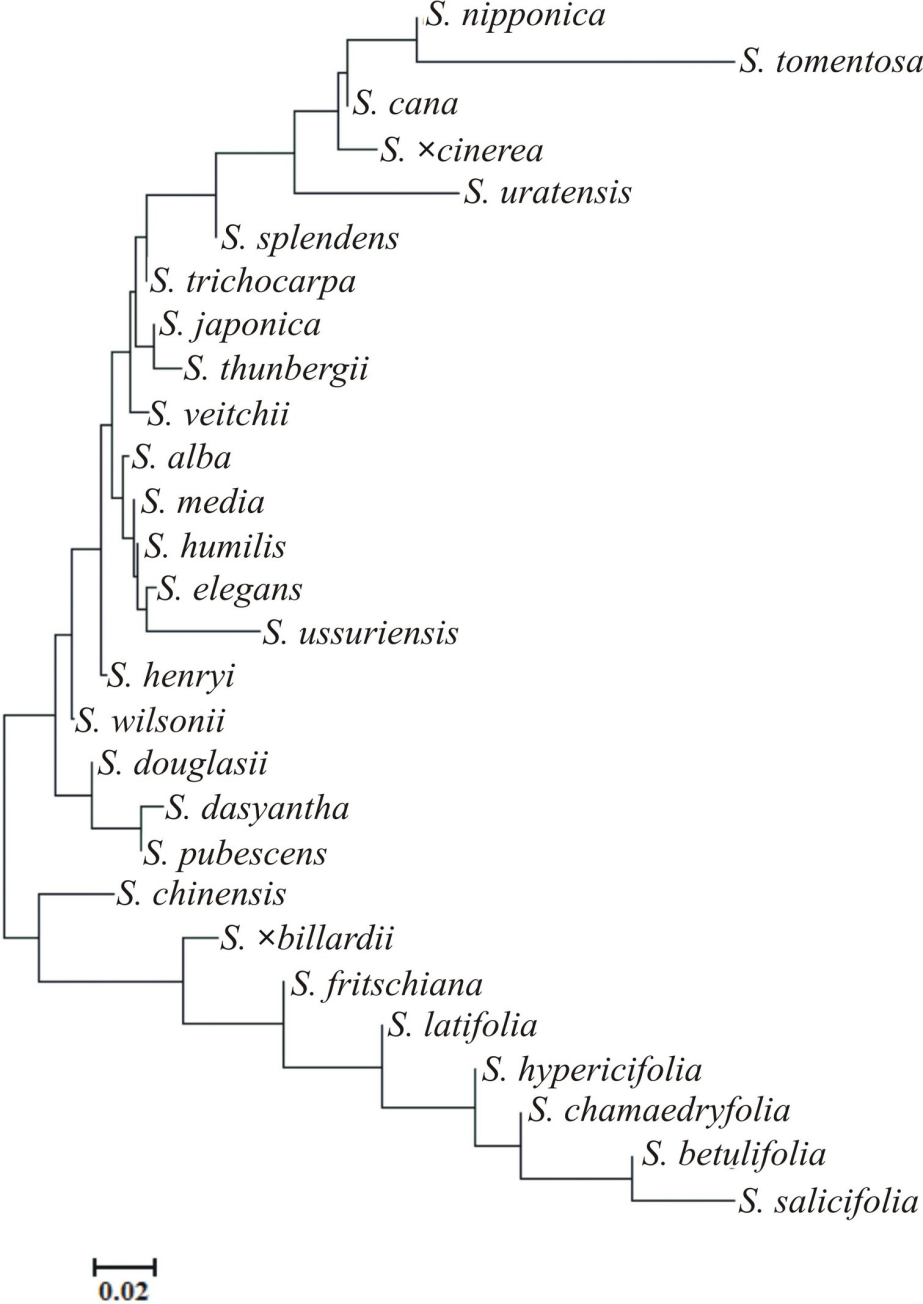
The phylogenetic tree of distribution of pollen morphological characters.

In the presented clustering according to the neighbor method of Euclidean distances, all the examined *Spiraea* species were divided into three groups (Fig 11). The first group included eight species: *S*. *alba* var. *latifolia*, *S*. *×billardii*, *S*. *chamaedryfolia*, *S*. *hypericifolia*, *S*. *betulifolia*, *S*. *salicifolia* and *S*. *chinensis*. The second one consisted of five species: *S*. *henri*, *S*. *wilsonii*, *S*. *douglasii*, *S*. *dasyantha* and *S*. *pubescens*. The third one comprised all the other species and was divided into two subgroups: A–*S*. *alba*, *S*. *media*, *S*. *elegans*, and B–all other species (Fig 11). The phylogenetic tree of distribution of pollen morphological characters was presented on the Fig 12. The obtained divisions were very similar to those presented in the dendrogram (Fig 11).

**Fig 13:**
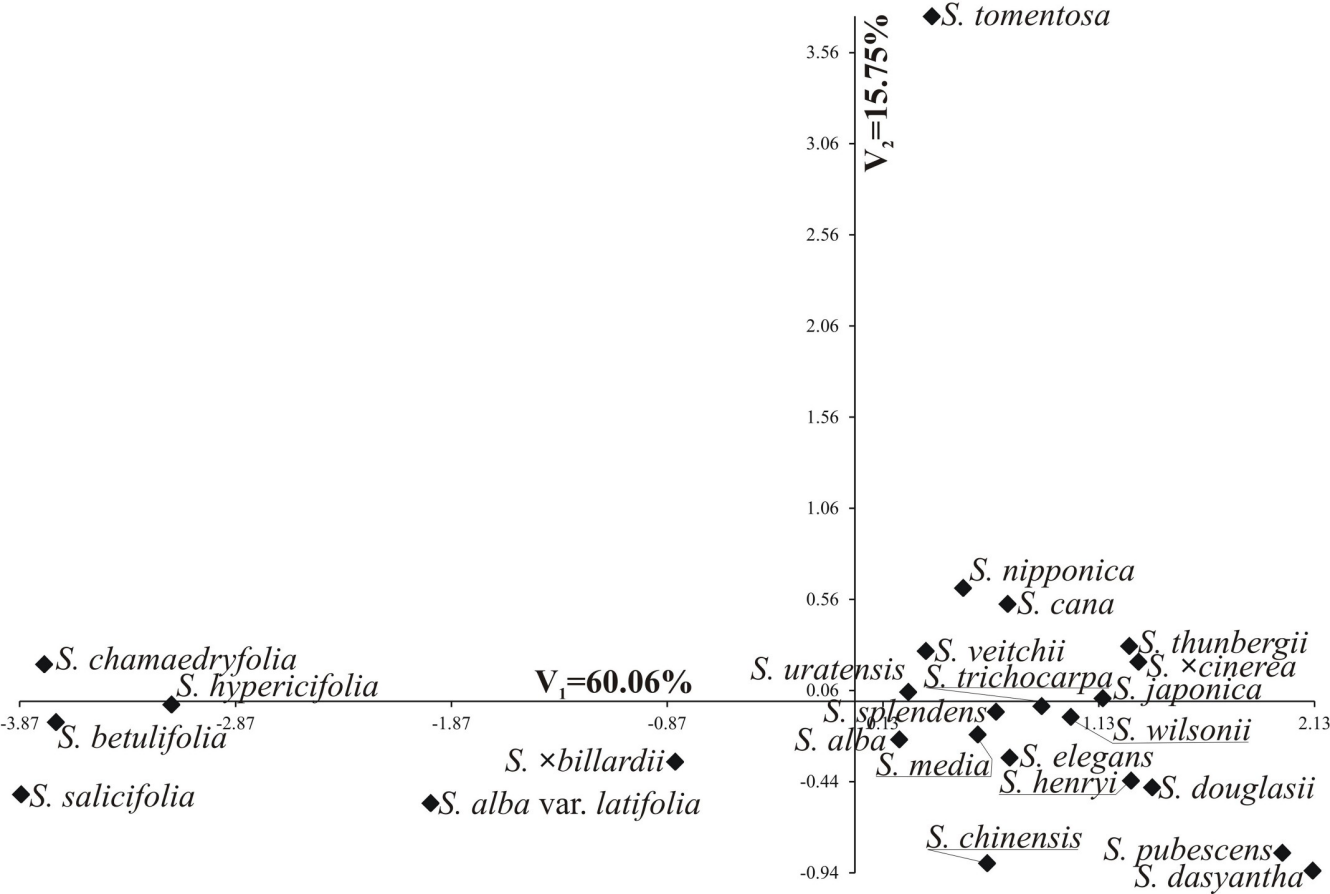
Distribution of 28 *Spiraea* taxa in space of two first canonical variables.

Fig 13 shows variability of the quantitative traits in the 25 *Spiraea* taxa under investigation in terms of the first two canonical variables. In the graph coordinates of the point for particular species are values for the first and second canonical variables, respectively. The first two canonical variables accounted for 80.08% of the total multivariate variability between individual species. Significant positive linear relationships with the first canonical variable were found for Exe, P/E, Exp/P and Exe/E (Table 8), while the first canonical variable correlated negatively with P, E and Le (Table 8). The second canonical variable had a significant positive correlation with Exp, Exe, Exp/P and Exe/E, but a significant negative correlation with Le/P (Table 8).

**Table 8:** Correlation coefficients between the first two canonical variables and original traits.

Trait	First canonical variable	Second canonical variable
P	-0.9376***	0.067
E	-0.986***	0.054
Le	-0.869***	-0.271
Exp	0.381	0.817***
Exe	0.441*	0.612***
P/E	0.537**	0.022
Le/P	-0.160	-0.811***
Exp/P	0.581**	0.730***
Exe/E	0.680***	0.459*
Percentage of explained multivariate variability	66.06	15.75

* P<0.05

** P<0.01

*** P<0.001

The greatest variation in terms of all the traits, based on the Mahalanobis distances measured, was found for the species *S*. *salicifolia* and the invasive *S*. *dasyantha* (the Mahalanobis distance between them amounted to 6.138). The greatest similarity was found for the species *S*. *japonica and S*. *wilsonii* (0.432). The values of the Mahalanobis distances for all the pairs of treatments are presented in Table 9. Pollen of the invasive *S*. *tomentosa* was distinctive due to it exhibiting the greatest variation in terms of all the traits together, based on the Mahalanobis distances measured. Pollen of these species was characterized by a relatively narrow range of E values (14–18 μm), the largest range of the P/E ratio (0.78–1.29) and the highest number of pollen grains (20) with the thickest exine (2 μm) (Tables 3–5).

**Table 9:** Mahalanobis distances between analyzed *Spiraea* species were calculated based on nine quantitative traits.

Species	*S*. *alba*	*S*. *alba* var. *latifolia*	*S*. *betulifolia*	*S*. *cana*	*S*. *chamaedryfolia*	*S*. *chinensis*	*S*. *dasyantha*	*S*. *douglasii*	*S*. *elegans*	*S*. *henryi*	*S*. *hypericifolia*	*S*. *japonica*	*S*. *media*	*S*. *nipponica*	*S*. *pubescens*	*S*. *salicifolia*	*S*. *splendens*	*S*. *thunbergii*	*S*. *tomentosa*	*S*. *trichocarpa*	*S*. *uratensis*	*S*. *veitchii*	*S*. *wilsonii*	*S*. *×billardii*	*S*. *×cinerea*
	1	2	3	4	5	6	7	8	9	10	11	12	13	14	15	16	17	18	19	20	21	22	23	24	25
**1**																									
**2**	2.36																								
**3**	4.19	2.59																							
**4**	1.82	3.44	4.80																						
**5**	4.12	2.63	1.87	4.70																					
**6**	1.12	2.86	4.67	2.51	4.69																				
**7**	2.26	4.36	6.02	2.77	6.10	1.68																			
**8**	1.48	3.51	5.28	1.84	5.31	1.27	1.26																		
**9**	0.84	2.96	4.70	1.97	4.63	1.28	1.78	1.36																	
**10**	1.25	3.47	5.14	1.84	5.19	1.24	1.27	0.97	0.93																
**11**	3.48	2.01	1.86	4.17	0.70	4.04	5.46	4.67	4.00	4.56															
**12**	1.21	3.34	4.98	1.36	5.06	1.54	1.76	0.86	1.20	0.86	4.45														
**13**	0.60	2.83	4.54	1.75	4.42	1.09	1.92	1.26	0.64	0.93	3.79	1.00													
**14**	1.33	3.06	4.64	1.16	4.46	2.25	2.84	1.98	1.67	1.77	3.92	1.39	1.46												
**15**	2.12	4.21	5.86	2.44	5.93	1.69	0.44	1.03	1.65	1.10	5.30	1.54	1.79	2.64											
**16**	4.22	2.12	1.62	4.91	1.81	4.67	6.14	5.32	4.76	5.22	1.66	5.11	4.59	4.75	5.95										
**17**	0.97	2.84	4.57	1.38	4.64	1.59	2.15	1.22	1.22	1.28	4.02	0.92	1.20	1.15	1.92	4.68									
**18**	1.44	3.58	5.19	1.48	5.17	1.81	2.01	1.26	1.44	1.13	4.58	0.61	1.12	1.37	1.85	5.32	1.38								
**19**	4.09	5.04	5.81	3.88	5.66	4.70	5.05	4.45	4.22	4.41	5.33	4.01	4.08	3.67	4.93	6.11	4.06	3.85							
**20**	0.77	3.02	4.78	1.48	4.75	1.38	1.88	1.12	0.75	0.78	4.13	0.61	0.60	1.17	1.70	4.84	0.85	0.82	3.99						
**21**	1.82	2.74	4.82	1.91	4.54	2.47	3.21	2.21	2.21	2.41	3.95	2.12	2.16	1.59	2.97	4.55	1.48	2.37	4.38	1.93					
**22**	0.84	2.66	4.32	1.59	4.28	1.59	2.55	1.59	1.44	1.58	3.69	1.10	1.04	1.02	2.41	4.46	1.01	1.15	3.75	0.96	1.83				
**23**	1.12	3.20	4.84	1.51	4.93	1.27	1.73	0.76	1.29	0.92	4.31	0.43	0.98	1.51	1.55	4.97	0.93	0.82	4.04	0.77	2.12	1.01			
**24**	2.02	1.58	3.68	2.70	3.84	2.51	3.72	2.67	2.61	2.95	3.22	2.58	2.50	2.48	3.52	3.41	1.99	2.92	4.66	2.45	1.75	2.07	2.45		
**25**	2.00	3.76	5.37	0.95	5.37	2.52	2.43	1.64	1.90	1.62	4.79	1.28	1.89	1.58	2.06	5.37	1.43	1.51	4.22	1.45	1.97	1.97	1.55	2.88	

Dα = 3.73

## Discussion

Palynological studies on the species from the genus *Spiraea* L. are not numerous. The least amount of research has been carried out in Europe [32], perhaps due to the small number of native species (7), and also in North America [23]. Most research papers come from Asia, which is the world center for the distribution of this genus [38–40, 63]. According to previous studies, the diagnostic features of the *Spiraea* pollen grains were exine ornamentation (striae and groove length, width and direction, and perforation number and diameter) and P and the P/E ratio [23, 32, 38–40], as well as endoaperture diameter [38]. In the opinion of Liu et al. [39] as well as the authors of this study, P or the P/E ratio were of a lesser diagnostic value, because the values of both these traits were often quite similar in all the studied taxa. Neither this study nor studies carried out by other researchers confirmed the observation of Polyakova and Gataulina [38] that endoapertures of some *Spiraea* species can be wide (*S*. *hypericifolia*, *S*. *salicifolia*), while others narrow (*S*. *alpine*, *S*. *crenata*, *S*. *schlothaurae*).

For the genus *Spiraea* other palynologists distinguished a striate or rarely striate-granulate exine ornamentation [23, 38, 39, 63]. Only the striate exine ornamentation was reported by Wrońska-Pilarek et al. [32] in *S*. *tomentosa*, while in the study presented here striate were observed in only one species, where it was the striate-reticulate ornamentation. All authors agree that the most important features of *Spiraea* pollen were the striae and the groove course and dimensions, as well as the presence or absence of perforations. Polyakova and Gataulina [38] reported that in the meadowsweet species the striae were variable in length and width. They crossed and branched, for the most part meridionally and in different directions. In the opinion of the above-mentioned authors, *S*. *salicifolia* was characterized by the ‘four-grooved’ pollen grains. However, such a feature was not found in the study presented here. Liu et al. [39] claimed that in the *Spiraea* species exine ornamentation was ‘fringe and stripe reticular’. Song et al. [40] recognized that all Spiraeeae taxa have a striate exine ornamentation. Indeed, in their opinion four types of striate exine ornamentation could be recognized. The first three types could each be divided into two subtypes based on the diameter of the perforations, which may be small or large. The significant importance of the diameters and number of perforations in the diagnosis of *Spiraea* species was also emphasized by Wrońska-Pilarek et al. [32].

The pollen grains of *Spiraea* species were described most often as small-sized [32, 38–40, 43, 44, 64] and rarely as medium-sized [38, 40]. In the research presented here, all the studied pollen grains were small. Additionally, Polyakova and Gataulina [38] reported that the significant variability of pollen size was typical of *S*. *media* and *S*. *alpine*. However, these results (P of 17.8–21.5 μm) for *S*. *media* were not confirmed in this study–the P and E values were average, ranging between 14 and 18 μm (Table 3). The differences may be due to the fact that the cited authors examined more samples (5) of *S*. *media* from populations scattered from Siberia to the Far East. Palynologists have mentioned different pollen shape types in the meadowsweet species. These pollen grains ranged from prolate and subspheroidal, spherical, trilobate, dolicho-round, oblong and fusiform [39], through elongated spheroidal [38], and from oblate to prolate [40]. The shape of the pollen in *S*. *tomentosa* was most frequently spheroidal, rarely oblate-spheroidal or prolate-spheroidal and very rarely subprolate or prolate [32]. The results in this study indicate that pollen shape in the 25 studied taxa was most frequently spheroidal and prolate-spheroidal, much less often oblate-spheroidal, suboblate or subprolate, and very rarely prolate. The reason for these differences may be connected with the detailed description of pollen shape classes in the last two studies.

The authors, as well as the other cited researchers, did not find Ubisch bodies (orbicules) in the studied taxa. These results were confirmed by Song et al. [40], who noted that orbicule distribution patterns indicated that the absence of orbicules was a synapomorphic condition of the more derived clade, comprising the genera *Pentactina*, *Petrophytum*, *Kelseya* and *Spiraea*.

Of the three invasive species studied (*S*. *douglasii*, *S*. *japonica* and *S*. *tomentosa*), the first two were not distinguished by specific pollen features, while *S*. *tomentosa* was distinctive as showing the greatest variation in terms of all the traits jointly, based on the Mahalanobis distances measured. It was found that the pollen features of this species differed from the other studied taxa in terms of the relatively narrow range of E, the largest range of the P/E ratio and the highest number of pollen grains with the thickest exine. In earlier studies by Wrońska-Pilarek et al. [32] carried out on a sample of 900 pollen grains of *S*. *tomentosa*, similar data was obtained. Thus, it could be concluded that the invasive *S*. *tomentosa* described shows a large variability of pollen features and a thick exine, which may indicate a greater ability to adapt its pollen to changing environmental conditions, guaranteeing it greater efficiency in taking over new areas. However, in the authors’ opinion to confirm this thesis the pollen of other invasive plant species should be compared, not only pollen from the genus *Spiraea*.

For the first time the intrageneric and interspecific variability of *Spiraea* pollen grains were studied based on nine quantitative traits. Statistical analysis of the investigated features indicated high variability among the species. The most variable species for all the nine traits jointly were *S*. *×billardii*, *S*. *veitchii*, *S*. *nipponica* and *S*. *cana*, while those with lower variability were *S*. *dasyantha*, *S*. *elegans*, *S*. *trichocarpa* and *S*. *chamaedryfolia*. The most variable biometric traits were the E and P, while lower variability was recorded in the Le/P ratio and Exe. The ranking of variability in the observed traits is as follows: E > P > Le > Exp/P > Exe/E > Exp > P/E > Exe > Le/P.

It needs to be stressed here that the above conclusions were drawn based on studies conducted applying the method of collecting flowers from single shrubs of individual taxa, an approach commonly used in palynology. Thus the recorded results may not illustrate the entire pollen variability in the investigated species. We are aware of this limitation in the sampling procedure; nevertheless, we are of an opinion that due to the definitely conservative character of pollen grains the obtained results are representative. This thesis is confirmed by the results of our numerous palynological studies, such as Wrońska-Pilarek et al. [31, 34, 65, 66] concerning pollen morphology and variability in dozens of species from many genera (e.g. *Crataegus* L., *Rosa* L., *Rubus* L., *Salix* L., *Spiraea* L.). They were performed using the same method and they were typically comparable to data cited in the above-mentioned papers given by other palynologists. A similar situation is found in the genus *Spiraea*.

## Conclusions

- The most useful pollen morphological features included exine ornamentation type or subtype determined based on traits of grooves and striae (length, width and course) and perforations (presence or absence, number, diameter). The other biometric features (pollen size and shape, exine thickness) turned out to be useless, as they were similar in all the studied taxa.
- The presented results showed that the morphological traits of pollen grains from 25 *Spiraea* taxa made it possible to isolate then individual species, while the other species formed groups of typically 2–3, up to 8 species. The same groups included species both from different and the same sections and series, including related and unrelated ones. Therefore, the pollen features do not fully confirm the classical taxonomic division of the studied genus. Thus, the morphological features of the pollen can be used in the taxonomy of the genus *Spiraea* as auxiliary traits to describe a particular species and taxonomic relations within the examined genus.
- The statistical analysis of the studied pollen traits indicated high variability among the tested species. The most variable biometric traits were E and P, while lower variability was observed for the Le/P ratio and Exe.
- Pollen of the invasive *S*. *tomentosa* differed from the other studied taxa in terms of the narrow range of the E values, the largest range of the P/E ratio and the highest number of pollen grains with the thickest exine. Pollen of the other invasive species (*S*. *douglasi* and *S*. *japonica*) did not differ significantly from the other studied species.

## Supporting information

S1 Table Complete morphological observations of quantitative features. (PDF)

S1 Fig LM micrographs of equatorial view of pollen showing colpori and exine ornamentation features (exine thickness). A, *S*. *alba*; B, *S*. *betulifolia*; C, *S*. *cana*; D, *S*. *chamaedryfolia*; E, *S*. *chinensis*; F, *S*. *dasyantha*; G, *S*. *douglasii*; H, *S*. *elegans*; I, *S*. *henryi*; J, *S*. *hypericifolia*; K, *S*. *alba* var. *latifolia*; L, *S*. *media*; M, *S*. *media*; N, *S*. *nipponica*; O, *S*. *pubescens*; P, *S*. *salicifolia*; Q, *S*. *splendens*; R, *S*. *thunbergii*; S, *S*. *trichocarpa*; T, *S*. *uratensis*; U, *S*. *veitchii*; V, *S*. *wilsonii*; W, *S*. *×billardii*; X, *S*. *×cinerea*; Y, *S*. *tomentosa*, A-Y.
(PDF)

## References

[1] Rosaceae Kalkman C.. In: Kubitzki K, editor. The families and genera of vascular plants, Vol. 6. Flowering Plants–Dicotyledons: Celastrales, Oxalidales, Rosales, Cornales, Ericales. Berlin: Springer; 2004. pp. 2–3.

[2] Potter D, Eriksson T, Evans RC, Oh S, Smedmark JEE, Morgan DR, et al. Phylogeny and classification of Rosaceae. Plant Syst Evol. 2007; 266: 5–43. doi: 10.1007/s00606-007-0539-9

[3] Potter D, Still SM, Grebenc T, Ballian D, Božič G, Franjiæ J, et al. Phylogenetic relationships in tribe *Spiraeeae* (Rosaceae) inferred from nucleotide sequence data. Plant Syst Evol. 2007; 266: 105–118. doi: 10.1007/s00606-007-0544-z

[4] Angiosperm Phylogeny Group IV. An update of the angiosperm phylogeny group classification for the orders and families of flowering plants. Bot J Linn Soc. 2016; 181: 1–20. doi: 10.1111/boj.12385

[5] Rehder A. Manual of Cultivated Trees and Shrubs Hardy in North America. New York: Macmillan Company; 1927.

[6] Yü TT, Kuan KC. Taxa Nova Rosacearum Sinicarum (I), I. *Spiraea* L., Systema *Spiraeae* Sinicae. Acta Phytotax. Sinica 1963; 8: 214–217.

[7] Phipps JB. Rosaceae Jussieu; 2018 [Cited 2021 March 15]. Database: Flora of North America [Internet]. Available from: http://www.efloras.org/florataxon.aspx?flora_id=1&taxon_id=10776

[8] Wu ZY, Raven PH, Hong DY. Flora of China. Vol. 9: Pittosporaceae through Connaraceae. Beijing: Science Press and St. Louis: Missouri Botanical Garden Press; 2003.

[9] Kurtto A, Lampinen R, Junikka L. Atlas Florae Europaeae. Distribution of vascular plants in Europe. Vol. 13. Rosaceae (*Spiraea* to *Fragaria*, excl. *Rubus*). Helsinki: The Committee for Mapping the Flora of Europe & Societas Biologica Fennica Vanamo; 2004.

[10] Tutin TG, Heywood WH, Burges NA, Moore DM, Valentine DH, Walters SM, et al. Vol. 2: Flora Europaea. Cambridge: Cambridge University Press; 1986.

[11] Hegi G. Illustrierte Flora von Mitteleuropa. 4/2A. *Spiraea* L. Spermatophyta: Angiospermae: Dicotyledones 2/2, 3rd ed. Berlin: Blackwell; 1995.

[12] Dajdok Z, Nowak A, Danielewicz W, Kujawa-Pawlaczyk J, Bena W. Invasive Alien Species Fact Sheet–*Spiraea tomentosa*. NOBANIS. 2011 March 20 [Cited 2021 March 10]. Available from: https://www.nobanis.org/globalassets/speciesinfo/s/spiraea-tomentosa/spiraea_tomentosa.pdf

[13] Brusa G, Sartori M, Cerabolini B. Analysis of Reproductive Strategies in an Invasive Alien Species, *Spiraea japonica* L., for Planning Control Actions. Italian Botanist 2008; 40: 143–150.

[14] Guillerme S, Barcet H, Munnik N, Maire E, Marais-Sicre C. Evolution of traditional agroforestry landscapes and development of invasive species: lessons from the Pyrenees (France). Sustainability Science 2020; 15: 1285–1299. doi: 10.1007/s11625-020-00847-1

[15] Tokarska-Guzik B, Dajdok Z, Zając M, Zając A, Urbisz A, Danielewicz W, et al. Rośliny obcego pochodzenia w Polsce ze szczególnym uwzględnieniem gatunków inwazyjnych. Warszawa: Generalna Dyrekcja Ochrony Środowiska; 2012. [in Polish].

[16] Lambdon PW, Pyšek P, Basnou C, Hejda M, Arianoutsou M, Essl F, et al. Alien flora of Europe: species diversity, temporal trends, geographical patterns and research needs. Preslia 2008; 80: 101–149.

[17] DAISIE. Handbook of Alien Species in Europe. Netherlands: Springer; 2009.

[18] Mirek Z, Piękoś-Mirkowa H, Zając A, Zając M. Flowering plants and pteridophytes of Poland. A checklist. Biodiversity of Poland. Vol. 1. Kraków: W. Szafer Institute of Botany, Polish Academy of Sciences; 2002.

[19] Silverside AJ. The nomenclature of some hybrids of the *Spiraea salicifolia* group naturalized in Britain. Watsonia 1990; 18: 147–151.

[20] Reitsma TJ. Pollen morphology of some European Rosaceae. Acta Bot Neerl. 1966; 15: 290–379.

[21] Eide F. Key for Northwest European Rosaceae pollen. Grana 1981; 20: 101–118.

[22] Marcucci MC, Sansavini S, Ciampolini F, Cresti M. Distinguish apple clones and cultivars by surface morphology and pollen physiology. J Am Soc Hort Sci. 1984; 109: 10–19.

[23] Hebda RJ, Chinnappa CC. Studies on pollen morphology of Rosaceae. Canada. Rev. Paleobot. Palynol. 1990; 64: 103–108. doi: 10.1016/0034-6667(90)90123-Z

[24] Ueda Y. Pollen surface morphology in the genus Rosa, related genera. Jpn J Palynol. 1992; 38: 94–105.

[25] Ueda Y, Okada Y. Discrimination of rose cultivar groups by pollen surface structure. J Hortic Sci. 1994; 69: 601–607.

[26] Hebda RJ, Chinnappa CC. Studies on pollen morphology of Rosaceae. Bot Lett. 1994; 141: 183–193.

[27] Popek R. Biosystematyczne studia nad rodzajem Rosa L. w Polsce i krajach ościennych. Kraków: Wydawnictwo Naukowe Wyższej Szkoły Pedagogicznej; 1996.

[28] Shinwari M, Khan MA. Pollen morphology of wild roses from Pakistan. Hamdard Medicus. 2004; 474: 5–13.

[29] Chung KS, Elisens WJ, Skvarla JJ. Pollen morphology and its phylogenetic significance in tribe Sanguisorbeae (Rosaceae). Plant Syst Evol. 2010; 285: 139–148. doi: 10.1007/s00606-009-0262-9

[30] Wrońska-Pilarek D, Jagodziński AM. Systematic importance of pollen morphological features of selected species from the genus Rosa (Rosaceae). Plant Syst Evol. 2011; 295: 55–72. doi: 10.1007/s00606-011-0462-y

[31] Wrońska-Pilarek D, Bocianowski J, Jagodziński AM. Comparison of pollen grain morphological features of selected species of the genus Crataegus L. (Rosaceae) and their spontaneous, interspecific hybrids. Bot J Linn Soc. 2013; 172: 555–571. doi: 10.1111/boj.12033

[32] Wrońska-Pilarek D, Wiatrowska B, Bocianowski J. Pollen morphology and variability of invasive Spiraea tomentosa L. (Rosaceae) from populations in Poland. PLoS ONE. 2019; 14: e0218276. doi: 10.1371/journal.pone.0218276 31442232 PMC6707766

[33] Xiong X, Zhou X, Li M, Xul B, Dengl H, Yul Q, et al. Pollen morphology in Rubus (Rosaceae) and its taxonomic implications. Plant Syst Evol. 2019; 305: 705–716. doi: 10.1007/s00606-019-01600-7

[34] Lechowicz K, Wrońska-Pilarek D, Bocianowski J, Maliński T. Pollen morphology of Polish species from the genus Rubus L. (Rosaceae) and its systematic importance. PLoS ONE. 2020; e0221607. doi: 10.1371/journal.pone.0221607 32469903 PMC7259507

[35] Ferguson IK. Pollen-morphological data in systematics and evolution: past, present and future. In: Plant systematics for the 21st century: Proceedings from a symposium held at the Wenner-Gren Centre. Stockholm: Portland Press; 2000. pp. 179–192.

[36] Matsuta N, Omura M, Akihama T. Difference in micromorphological pattern on pollen surface of Japanese Pear cultivars. Jpn J Breed. 1982; 32: 123–128.

[37] Wrońska-Pilarek D. Pollen morphology of Polish native species of the Rosa genus (Rosaceae) and its relation to systematic. Acta Soc Bot Polon. 2011; 80: 221.

[38] Polyakova TA, Gataulina GN. Morphology and variability of pollen of the genus *Spiraea* L. (Rosaceae) in Siberia and the Far East. Contemp Probl Ecol. 2008; 1: 420–424. doi: 10.1134/S199542550804005X

[39] Liu HM, Li L, Xing YQ, Lu G, Chen YJ. Pollen morphology of 18 species of *Spiraea* L. and its taxonomic significance. J. China Agric. Univ. 2010; 15: 42–48. [in Chinese].

[40] Song S, Min-Kyeong O, Hee-Seon R, Suk-Pyo H. Morphology of pollen and orbicules in the tribe *Spiraeeae* (Rosaceae) and its systematic implications. Grana. 2017; 56: 351–367. doi: 10.1080/00173134.2016.1274334

[41] Pathak ML, Idrees M, Xu B, GAO XF. Pollen morphology of subfamily Maloideae (Rosaceae) with special focus on the genus *Photinia*. Phytotaxa 2019; 404: 171–189. doi: 10.11646/phytotaxa.404.5.1

[42] Fogle HW. Identification of clones within four tree species by pollen exine patterns. J Am Soc Hort Sci. 1977; 102: 552–560.

[43] Heigl H. *Spiraea chamaedryfolia*; 2021 [Cited 2021 March 11]. Database: PalDat—Palynological Database [Internet]. Available from: https://www.paldat.org/pub/Spiraea_chamaedryfolia/305180

[44] Auer W. *Spiraea oblongifolia*; 2021 [Cited 2021 Mar 11]. Database: PalDat—Palynological Database [Internet]. Available from: https://www.paldat.org/pub/Spiraea_oblongifolia/305269

[45] Komarov VL. Flora of the U.S.S.R 9, Rosales and Sarraceniales. Izdatel’stvo Akademii Nauk SSSR. Moskva: Israel Program for Scientific Translation; 1939.

[46] Sokolov S. Deriewa i kustraniki SSSR vol. 3. Moskwa: Izdatielstwo Akademii Nauk SSSR; 1954.

[47] Polozhij AV, Malyschev LI. Flora Sibiriae. Rosaceae. Nowosybirsk: Izdatel’stvo Nauka, Divisio Sibirica; 1988.

[48] Ohwi J. Flora of Japan. Washington DC: Smithsonian Institution; 1965.

[49] Lu L, Crinan A. Spiraea. In: Zhengyi W, Raven PH, editors. Flora of China, vol. 9, Pittosporaceae through Connaraceae. St. Louis: Missouri Botanical Garden Press; 2003. pp. 47–73.

[50] Cullen J, Alexander JCM, Brady A, Brickel CD, Green PS, Heywood VH, et al. The European Garden Flora vol. 4. Cambridge: Cambridge University Press; 1995.

[51] The Plant List. A working list of all plant species; 2013 [cited 2021 Dec 2]. Database: The plant list [Internet]. Available from: http://www.theplantlist.org

[52] POWO “Plants of the World Online”. Facilitated by the Royal Botanic Gardens, Kew; 2021 [cited 2021 Dec 3]. Database: Plant names [Internet]. Available from: https://powo.science.kew.org

[53] Wrońska-Pilarek D, Jagodziński AM, Bocianowski J, Janyszek M. The optimal sample size in pollen morphological studies using the example of *Rosa canina* L.–Rosaceae. Palynology. 2015; 39: 56–75. doi: 10.1080/01916122.2014.933748

[54] Erdtman G. Pollen morphology and plant taxonomy. Angiosperms. An introduction to palynology. 1st ed. Stockholm: Almquist and Wiksell; 1952.

[55] Erdtman G. The acetolysis method. A revised description. Svensk Bot Tidskr. 1960; 54: 561–564.

[56] Punt W, Hoen PP, Blackmore S, Nilsson S, Le Thomas A. Glossary of pollen and spore terminology. Rev Palaeobot Palynol. 2007; 143: 1–81. doi: 10.1016/j.revpalbo.2006.06.008

[57] Halbritter H, Hess Ulrich S, Grímsson F, Weber M, Zetter R, Hesse M., et al. Illustrated Pollen Terminology. 2nd ed. Vienna: Springer; 2018.

[58] Shapiro SS, Wilk MB. An analysis of variance test for normality (complete samples). Biometrika 1965; 52: 591–611.

[59] Rencher AC. Interpretation of canonical discriminant functions, canonical variates, and principal components. Am. Stat. 1992; 46: 217–225.

[60] Seidler-Łożykowska K, Bocianowski J. Evaluation of variability of morphological traits of selected caraway (*Carum carvi* L.) genotypes. Industrial Crops Prod. 2012; 35: 140–145.

[61] Mahalanobis PC. On the generalized distance in statistics. Proc Natl Inst Sci India. 1936; 12: 49–55. doi: 10.1016/j.indcrop.2011.06.026

[62] Camussi A, Ottaviano E, Caliński T, Kaczmarek Z. Genetic distances based on quantitative traits. Genetics 1985; 111: 945–962. doi: 10.1093/genetics/111.4.945 4065546 PMC1202682

[63] Ueda Y, Tomita H. Morphometric analysis of pollen patterns in Roses. Hort J. 1989; 58: 211–220.

[64] Zhou L, Wei ZX, Wu ZY. Pollen morphology of Spiraeoideae in China (Rosaceae). Acta Bot Yunnanica. 1999; 21: 303–308.

[65] Wrońska-Pilarek D, Halbritter H, Krzymińska A, Bednorz L, Bocianowski J. Pollen morphology and variability of selected European species of the genus *Allium* L. (Alliaceae). Acta Sci Pol-Hortoru. 2016; 15: 65–84.

[66] Maciejewska-Rutkowska I, Bocianowski J, Wrońska-Pilarek D. Pollen morphology and variability of Polish native species from genus Salix L. PLoS ONE. 2021; e0243993. doi: 10.1371/journal.pone.0243993 33600499 PMC7891718

