# Supplementary material for: Pollen morphology and variability of native and alien, including invasive, species of the genus Spiraea L. (Rosaceae) in Poland
Source: PLoS One. 2022 Aug 29;17(8):e0273743. doi: 10.1371/journal.pone.0273743 (PMC9423682; doi:10.1371/journal.pone.0273743)
Supplement: S1 Table — (PDF) [file pone.0273743.s001.pdf]

S1 Table. Complete morphological observations of quantitative features.

| Species | No | Repl | P | E | Le | Exp | Exe | P/E | Le/P | Exp/P | Exe/E |
|---|---|---|---|---|---|---|---|---|---|---|---|
| *S. alba* | 1 | 1 | 16.00 | 16.00 | 12.00 | 0.60 | 0.60 | 1.00 | 0.75 | 0.04 | 0.04 |
| *S. alba* | 1 | 2 | 18.00 | 16.00 | 14.00 | 1.00 | 1.00 | 1.13 | 0.78 | 0.06 | 0.06 |
| *S. alba* | 1 | 3 | 16.00 | 16.00 | 16.00 | 1.00 | 1.00 | 1.00 | 1.00 | 0.06 | 0.06 |
| *S. alba* | 1 | 4 | 16.00 | 16.00 | 16.00 | 1.00 | 1.00 | 1.00 | 1.00 | 0.06 | 0.06 |
| *S. alba* | 1 | 5 | 16.00 | 16.00 | 14.00 | 1.60 | 1.60 | 1.00 | 0.88 | 0.10 | 0.10 |
| *S. alba* | 1 | 6 | 16.00 | 16.00 | 14.00 | 1.00 | 1.00 | 1.00 | 0.88 | 0.06 | 0.06 |
| *S. alba* | 1 | 7 | 16.00 | 16.00 | 12.00 | 1.00 | 1.00 | 1.00 | 0.75 | 0.06 | 0.06 |
| *S. alba* | 1 | 8 | 16.00 | 16.00 | 14.00 | 1.00 | 1.00 | 1.00 | 0.88 | 0.06 | 0.06 |
| *S. alba* | 1 | 9 | 16.00 | 16.00 | 16.00 | 1.00 | 1.00 | 1.00 | 1.00 | 0.06 | 0.06 |
| *S. alba* | 1 | 10 | 16.00 | 18.00 | 16.00 | 1.00 | 1.00 | 0.89 | 1.00 | 0.06 | 0.06 |
| *S. alba* | 1 | 11 | 16.00 | 16.00 | 14.00 | 0.60 | 0.60 | 1.00 | 0.88 | 0.04 | 0.04 |
| *S. alba* | 1 | 12 | 16.00 | 16.00 | 16.00 | 0.60 | 0.60 | 1.00 | 1.00 | 0.04 | 0.04 |
| *S. alba* | 1 | 13 | 18.00 | 16.00 | 18.00 | 1.60 | 1.60 | 1.13 | 1.00 | 0.09 | 0.10 |
| *S. alba* | 1 | 14 | 16.00 | 16.00 | 16.00 | 1.00 | 1.00 | 1.00 | 1.00 | 0.06 | 0.06 |
| *S. alba* | 1 | 15 | 18.00 | 18.00 | 16.00 | 0.60 | 0.60 | 1.00 | 0.89 | 0.03 | 0.03 |
| *S. alba* | 1 | 16 | 18.00 | 16.00 | 18.00 | 1.00 | 1.00 | 1.13 | 1.00 | 0.06 | 0.06 |
| *S. alba* | 1 | 17 | 14.00 | 16.00 | 14.00 | 1.00 | 1.00 | 0.88 | 1.00 | 0.07 | 0.06 |
| *S. alba* | 1 | 18 | 20.00 | 20.00 | 20.00 | 1.00 | 1.00 | 1.00 | 1.00 | 0.05 | 0.05 |
| *S. alba* | 1 | 19 | 16.00 | 16.00 | 14.00 | 1.00 | 1.00 | 1.00 | 0.88 | 0.06 | 0.06 |
| *S. alba* | 1 | 20 | 16.00 | 16.00 | 14.00 | 1.00 | 1.00 | 1.00 | 0.88 | 0.06 | 0.06 |
| *S. alba* | 1 | 21 | 16.00 | 14.00 | 16.00 | 1.00 | 1.00 | 1.14 | 1.00 | 0.06 | 0.07 |
| *S. alba* | 1 | 22 | 16.00 | 14.00 | 16.00 | 1.00 | 1.00 | 1.14 | 1.00 | 0.06 | 0.07 |
| *S. alba* | 1 | 23 | 16.00 | 16.00 | 14.00 | 1.00 | 1.00 | 1.00 | 0.88 | 0.06 | 0.06 |
| *S. alba* | 1 | 24 | 16.00 | 16.00 | 14.00 | 1.00 | 1.00 | 1.00 | 0.88 | 0.06 | 0.06 |
| *S. alba* | 1 | 25 | 16.00 | 16.00 | 16.00 | 0.20 | 0.20 | 1.00 | 1.00 | 0.01 | 0.01 |
| *S. alba* | 1 | 26 | 16.00 | 16.00 | 16.00 | 1.00 | 1.00 | 1.00 | 1.00 | 0.06 | 0.06 |
| *S. alba* | 1 | 27 | 16.00 | 16.00 | 16.00 | 1.00 | 1.00 | 1.00 | 1.00 | 0.06 | 0.06 |
| *S. alba* | 1 | 28 | 16.00 | 14.00 | 16.00 | 1.00 | 1.00 | 1.14 | 1.00 | 0.06 | 0.07 |
| *S. alba* | 1 | 29 | 16.00 | 16.00 | 14.00 | 1.60 | 1.60 | 1.00 | 0.88 | 0.10 | 0.10 |
| *S. alba* | 1 | 30 | 16.00 | 16.00 | 16.00 | 1.00 | 1.00 | 1.00 | 1.00 | 0.06 | 0.06 |
| *S. alba* var. *latifolia* | 2 | 1 | 20.00 | 18.00 | 14.00 | 0.60 | 0.60 | 1.11 | 0.70 | 0.03 | 0.03 |
| *S. alba* var. *latifolia* | 2 | 2 | 18.00 | 18.00 | 16.00 | 0.60 | 0.60 | 1.00 | 0.89 | 0.03 | 0.03 |
| *S. alba* var. *latifolia* | 2 | 3 | 18.00 | 18.00 | 16.00 | 1.00 | 1.00 | 1.00 | 0.89 | 0.06 | 0.06 |
| *S. alba* var. *latifolia* | 2 | 4 | 18.00 | 18.00 | 18.00 | 0.60 | 0.60 | 1.00 | 1.00 | 0.03 | 0.03 |
| *S. alba* var. *latifolia* | 2 | 5 | 18.00 | 18.00 | 18.00 | 0.20 | 0.20 | 1.00 | 1.00 | 0.01 | 0.01 |
| *S. alba* var. *latifolia* | 2 | 6 | 16.00 | 16.00 | 16.00 | 1.00 | 1.00 | 1.00 | 1.00 | 0.06 | 0.06 |
| *S. alba* var. *latifolia* | 2 | 7 | 16.00 | 16.00 | 16.00 | 1.00 | 1.00 | 1.00 | 1.00 | 0.06 | 0.06 |
| *S. alba* var. *latifolia* | 2 | 8 | 18.00 | 16.00 | 16.00 | 0.60 | 0.20 | 1.13 | 0.89 | 0.03 | 0.01 |
| *S. alba* var. *latifolia* | 2 | 9 | 18.00 | 18.00 | 16.00 | 1.00 | 1.00 | 1.00 | 0.89 | 0.06 | 0.06 |
| *S. alba* var. *latifolia* | 2 | 10 | 18.00 | 20.00 | 18.00 | 0.60 | 0.60 | 0.90 | 1.00 | 0.03 | 0.03 |
| *S. alba* var. *latifolia* | 2 | 11 | 18.00 | 18.00 | 18.00 | 0.60 | 0.60 | 1.00 | 1.00 | 0.03 | 0.03 |
| *S. alba* var. *latifolia* | 2 | 12 | 18.00 | 18.00 | 18.00 | 1.00 | 1.00 | 1.00 | 1.00 | 0.06 | 0.06 |
| *S. alba* var. *latifolia* | 2 | 13 | 20.00 | 18.00 | 20.00 | 1.00 | 1.00 | 1.11 | 1.00 | 0.05 | 0.06 |
| *S. alba* var. *latifolia* | 2 | 14 | 18.00 | 16.00 | 18.00 | 1.00 | 1.00 | 1.13 | 1.00 | 0.06 | 0.06 |
| *S. alba* var. *latifolia* | 2 | 15 | 18.00 | 18.00 | 18.00 | 1.00 | 1.60 | 1.00 | 1.00 | 0.06 | 0.09 |
| *S. alba* var. *latifolia* | 2 | 16 | 18.00 | 18.00 | 18.00 | 0.20 | 0.20 | 1.00 | 1.00 | 0.01 | 0.01 |
| *S. alba* var. *latifolia* | 2 | 17 | 18.00 | 18.00 | 18.00 | 0.20 | 0.20 | 1.00 | 1.00 | 0.01 | 0.01 |
| *S. alba* var. *latifolia* | 2 | 18 | 18.00 | 18.00 | 18.00 | 1.60 | 1.60 | 1.00 | 1.00 | 0.09 | 0.09 |
| *S. alba* var. *latifolia* | 2 | 19 | 18.00 | 16.00 | 18.00 | 1.00 | 1.00 | 1.13 | 1.00 | 0.06 | 0.06 |
| *S. alba* var. *latifolia* | 2 | 20 | 18.00 | 18.00 | 18.00 | 1.00 | 1.00 | 1.00 | 1.00 | 0.06 | 0.06 |
| *S. alba* var. *latifolia* | 2 | 21 | 18.00 | 18.00 | 18.00 | 0.60 | 0.60 | 1.00 | 1.00 | 0.03 | 0.03 |

| | | | | | | | | | | | |
|---|---|---|---|---|---|---|---|---|---|---|---|
| *S. alba* var. *latifolia* | 2 | 22 | 18.00 | 18.00 | 16.00 | 0.60 | 0.60 | 1.00 | 0.89 | 0.03 | 0.03 |
| *S. alba* var. *latifolia* | 2 | 23 | 18.00 | 18.00 | 18.00 | 1.60 | 1.60 | 1.00 | 1.00 | 0.09 | 0.09 |
| *S. alba* var. *latifolia* | 2 | 24 | 20.00 | 20.00 | 20.00 | 1.00 | 1.00 | 1.00 | 1.00 | 0.05 | 0.05 |
| *S. alba* var. *latifolia* | 2 | 25 | 18.00 | 20.00 | 16.00 | 0.60 | 0.60 | 0.90 | 0.89 | 0.03 | 0.03 |
| *S. alba* var. *latifolia* | 2 | 26 | 18.00 | 18.00 | 16.00 | 0.60 | 0.60 | 1.00 | 0.89 | 0.03 | 0.03 |
| *S. alba* var. *latifolia* | 2 | 27 | 20.00 | 18.00 | 18.00 | 0.60 | 0.60 | 1.11 | 0.90 | 0.03 | 0.03 |
| *S. alba* var. *latifolia* | 2 | 28 | 20.00 | 18.00 | 20.00 | 0.60 | 0.60 | 1.11 | 1.00 | 0.03 | 0.03 |
| *S. alba* var. *latifolia* | 2 | 29 | 18.00 | 18.00 | 18.00 | 0.60 | 0.60 | 1.00 | 1.00 | 0.03 | 0.03 |
| *S. alba* var. *latifolia* | 2 | 30 | 20.00 | 18.00 | 20.00 | 1.00 | 1.00 | 1.11 | 1.00 | 0.05 | 0.06 |
| *S. betulifolia* | 3 | 1 | 20.00 | 18.00 | 18.00 | 0.60 | 0.60 | 1.11 | 0.90 | 0.03 | 0.03 |
| *S. betulifolia* | 3 | 2 | 16.00 | 20.00 | 14.00 | 1.00 | 1.00 | 0.80 | 0.88 | 0.06 | 0.05 |
| *S. betulifolia* | 3 | 3 | 18.00 | 20.00 | 16.00 | 0.60 | 0.60 | 0.90 | 0.89 | 0.03 | 0.03 |
| *S. betulifolia* | 3 | 4 | 18.00 | 18.00 | 14.00 | 1.00 | 1.00 | 1.00 | 0.78 | 0.06 | 0.06 |
| *S. betulifolia* | 3 | 5 | 18.00 | 20.00 | 16.00 | 0.60 | 0.60 | 0.90 | 0.89 | 0.03 | 0.03 |
| *S. betulifolia* | 3 | 6 | 20.00 | 20.00 | 16.00 | 1.00 | 1.00 | 1.00 | 0.80 | 0.05 | 0.05 |
| *S. betulifolia* | 3 | 7 | 20.00 | 16.00 | 16.00 | 0.60 | 0.60 | 1.25 | 0.80 | 0.03 | 0.04 |
| *S. betulifolia* | 3 | 8 | 16.00 | 18.00 | 14.00 | 0.60 | 0.60 | 0.89 | 0.88 | 0.04 | 0.03 |
| *S. betulifolia* | 3 | 9 | 18.00 | 20.00 | 16.00 | 0.60 | 0.60 | 0.90 | 0.89 | 0.03 | 0.03 |
| *S. betulifolia* | 3 | 10 | 18.00 | 18.00 | 16.00 | 0.60 | 0.60 | 1.00 | 0.89 | 0.03 | 0.03 |
| *S. betulifolia* | 3 | 11 | 18.00 | 20.00 | 16.00 | 0.60 | 0.60 | 0.90 | 0.89 | 0.03 | 0.03 |
| *S. betulifolia* | 3 | 12 | 20.00 | 20.00 | 18.00 | 0.60 | 0.60 | 1.00 | 0.90 | 0.03 | 0.03 |
| *S. betulifolia* | 3 | 13 | 16.00 | 16.00 | 16.00 | 1.00 | 1.00 | 1.00 | 1.00 | 0.06 | 0.06 |
| *S. betulifolia* | 3 | 14 | 20.00 | 20.00 | 20.00 | 1.00 | 1.00 | 1.00 | 1.00 | 0.05 | 0.05 |
| *S. betulifolia* | 3 | 15 | 20.00 | 18.00 | 18.00 | 0.60 | 0.60 | 1.11 | 0.90 | 0.03 | 0.03 |
| *S. betulifolia* | 3 | 16 | 20.00 | 20.00 | 16.00 | 0.60 | 0.60 | 1.00 | 0.80 | 0.03 | 0.03 |
| *S. betulifolia* | 3 | 17 | 18.00 | 20.00 | 18.00 | 0.60 | 0.60 | 0.90 | 1.00 | 0.03 | 0.03 |
| *S. betulifolia* | 3 | 18 | 20.00 | 20.00 | 20.00 | 0.60 | 0.60 | 1.00 | 1.00 | 0.03 | 0.03 |
| *S. betulifolia* | 3 | 19 | 20.00 | 20.00 | 16.00 | 0.60 | 0.60 | 1.00 | 0.80 | 0.03 | 0.03 |
| *S. betulifolia* | 3 | 20 | 20.00 | 20.00 | 18.00 | 0.60 | 0.60 | 1.00 | 0.90 | 0.03 | 0.03 |
| *S. betulifolia* | 3 | 21 | 18.00 | 18.00 | 16.00 | 0.60 | 0.60 | 1.00 | 0.89 | 0.03 | 0.03 |
| *S. betulifolia* | 3 | 22 | 18.00 | 20.00 | 18.00 | 1.00 | 1.00 | 0.90 | 1.00 | 0.06 | 0.05 |
| *S. betulifolia* | 3 | 23 | 18.00 | 20.00 | 18.00 | 0.60 | 0.60 | 0.90 | 1.00 | 0.03 | 0.03 |
| *S. betulifolia* | 3 | 24 | 18.00 | 18.00 | 16.00 | 0.60 | 0.60 | 1.00 | 0.89 | 0.03 | 0.03 |
| *S. betulifolia* | 3 | 25 | 20.00 | 20.00 | 18.00 | 0.60 | 0.60 | 1.00 | 0.90 | 0.03 | 0.03 |
| *S. betulifolia* | 3 | 26 | 18.00 | 20.00 | 16.00 | 0.20 | 0.20 | 0.90 | 0.89 | 0.01 | 0.01 |
| *S. betulifolia* | 3 | 27 | 18.00 | 20.00 | 14.00 | 0.20 | 0.20 | 0.90 | 0.78 | 0.01 | 0.01 |
| *S. betulifolia* | 3 | 28 | 20.00 | 20.00 | 18.00 | 0.20 | 0.20 | 1.00 | 0.90 | 0.01 | 0.01 |
| *S. betulifolia* | 3 | 29 | 18.00 | 20.00 | 16.00 | 1.00 | 1.00 | 0.90 | 0.89 | 0.06 | 0.05 |
| *S. betulifolia* | 3 | 30 | 20.00 | 20.00 | 18.00 | 0.60 | 0.60 | 1.00 | 0.90 | 0.03 | 0.03 |
| *S. cana* | 4 | 1 | 14.00 | 12.00 | 12.00 | 1.60 | 1.60 | 1.17 | 0.86 | 0.11 | 0.13 |
| *S. cana* | 4 | 2 | 16.00 | 14.00 | 14.00 | 1.00 | 1.00 | 1.14 | 0.88 | 0.06 | 0.07 |
| *S. cana* | 4 | 3 | 18.00 | 16.00 | 18.00 | 1.60 | 1.60 | 1.13 | 1.00 | 0.09 | 0.10 |
| *S. cana* | 4 | 4 | 16.00 | 14.00 | 14.00 | 1.60 | 1.60 | 1.14 | 0.88 | 0.10 | 0.11 |
| *S. cana* | 4 | 5 | 16.00 | 16.00 | 14.00 | 1.00 | 1.00 | 1.00 | 0.88 | 0.06 | 0.06 |
| *S. cana* | 4 | 6 | 16.00 | 12.00 | 14.00 | 1.00 | 1.00 | 1.33 | 0.88 | 0.06 | 0.08 |
| *S. cana* | 4 | 7 | 16.00 | 16.00 | 14.00 | 1.60 | 1.60 | 1.00 | 0.88 | 0.10 | 0.10 |
| *S. cana* | 4 | 8 | 16.00 | 16.00 | 14.00 | 1.60 | 1.60 | 1.00 | 0.88 | 0.10 | 0.10 |
| *S. cana* | 4 | 9 | 16.00 | 14.00 | 14.00 | 1.00 | 1.00 | 1.14 | 0.88 | 0.06 | 0.07 |
| *S. cana* | 4 | 10 | 16.00 | 14.00 | 14.00 | 1.60 | 1.60 | 1.14 | 0.88 | 0.10 | 0.11 |
| *S. cana* | 4 | 11 | 16.00 | 12.00 | 16.00 | 1.60 | 1.60 | 1.33 | 1.00 | 0.10 | 0.13 |
| *S. cana* | 4 | 12 | 14.00 | 12.00 | 14.00 | 1.00 | 1.00 | 1.17 | 1.00 | 0.07 | 0.08 |
| *S. cana* | 4 | 13 | 16.00 | 16.00 | 14.00 | 1.00 | 1.00 | 1.00 | 0.88 | 0.06 | 0.06 |
| *S. cana* | 4 | 14 | 14.00 | 16.00 | 14.00 | 1.60 | 1.60 | 0.88 | 1.00 | 0.11 | 0.10 |
| *S. cana* | 4 | 15 | 14.00 | 16.00 | 14.00 | 1.00 | 1.00 | 0.88 | 1.00 | 0.07 | 0.06 |

| | | | | | | | | | | | |
|---|---|---|---|---|---|---|---|---|---|---|---|
| *S. cana* | 4 | 16 | 16.00 | 14.00 | 14.00 | 1.00 | 1.00 | 1.14 | 0.88 | 0.06 | 0.07 |
| *S. cana* | 4 | 17 | 16.00 | 14.00 | 14.00 | 1.00 | 1.00 | 1.14 | 0.88 | 0.06 | 0.07 |
| *S. cana* | 4 | 18 | 16.00 | 16.00 | 14.00 | 1.60 | 1.60 | 1.00 | 0.88 | 0.10 | 0.10 |
| *S. cana* | 4 | 19 | 16.00 | 18.00 | 16.00 | 1.00 | 1.00 | 0.89 | 1.00 | 0.06 | 0.06 |
| *S. cana* | 4 | 20 | 18.00 | 16.00 | 18.00 | 0.60 | 0.60 | 1.13 | 1.00 | 0.03 | 0.04 |
| *S. cana* | 4 | 21 | 16.00 | 16.00 | 14.00 | 1.60 | 1.60 | 1.00 | 0.88 | 0.10 | 0.10 |
| *S. cana* | 4 | 22 | 14.00 | 12.00 | 12.00 | 1.00 | 1.00 | 1.17 | 0.86 | 0.07 | 0.08 |
| *S. cana* | 4 | 23 | 16.00 | 18.00 | 14.00 | 1.60 | 1.60 | 0.89 | 0.88 | 0.10 | 0.09 |
| *S. cana* | 4 | 24 | 18.00 | 14.00 | 18.00 | 1.00 | 1.00 | 1.29 | 1.00 | 0.06 | 0.07 |
| *S. cana* | 4 | 25 | 16.00 | 16.00 | 16.00 | 1.00 | 1.00 | 1.00 | 1.00 | 0.06 | 0.06 |
| *S. cana* | 4 | 26 | 16.00 | 16.00 | 14.00 | 1.00 | 1.00 | 1.00 | 0.88 | 0.06 | 0.06 |
| *S. cana* | 4 | 27 | 18.00 | 14.00 | 16.00 | 1.60 | 1.60 | 1.29 | 0.89 | 0.09 | 0.11 |
| *S. cana* | 4 | 28 | 18.00 | 14.00 | 16.00 | 1.60 | 1.60 | 1.29 | 0.89 | 0.09 | 0.11 |
| *S. cana* | 4 | 29 | 16.00 | 16.00 | 12.00 | 0.60 | 0.60 | 1.00 | 0.75 | 0.04 | 0.04 |
| *S. cana* | 4 | 30 | 16.00 | 16.00 | 14.00 | 1.60 | 1.60 | 1.00 | 0.88 | 0.10 | 0.10 |
| *S. chamaedryfolia* | 5 | 1 | 18.00 | 20.00 | 18.00 | 1.60 | 1.60 | 0.90 | 1.00 | 0.09 | 0.08 |
| *S. chamaedryfolia* | 5 | 2 | 20.00 | 20.00 | 20.00 | 0.60 | 0.60 | 1.00 | 1.00 | 0.03 | 0.03 |
| *S. chamaedryfolia* | 5 | 3 | 18.00 | 18.00 | 16.00 | 1.00 | 1.00 | 1.00 | 0.89 | 0.06 | 0.06 |
| *S. chamaedryfolia* | 5 | 4 | 18.00 | 20.00 | 16.00 | 0.60 | 0.60 | 0.90 | 0.89 | 0.03 | 0.03 |
| *S. chamaedryfolia* | 5 | 5 | 16.00 | 20.00 | 16.00 | 0.60 | 0.60 | 0.80 | 1.00 | 0.04 | 0.03 |
| *S. chamaedryfolia* | 5 | 6 | 18.00 | 20.00 | 16.00 | 0.60 | 0.60 | 0.90 | 0.89 | 0.03 | 0.03 |
| *S. chamaedryfolia* | 5 | 7 | 20.00 | 20.00 | 20.00 | 1.60 | 1.60 | 1.00 | 1.00 | 0.08 | 0.08 |
| *S. chamaedryfolia* | 5 | 8 | 20.00 | 20.00 | 18.00 | 1.60 | 1.60 | 1.00 | 0.90 | 0.08 | 0.08 |
| *S. chamaedryfolia* | 5 | 9 | 20.00 | 20.00 | 20.00 | 1.60 | 1.60 | 1.00 | 1.00 | 0.08 | 0.08 |
| *S. chamaedryfolia* | 5 | 10 | 18.00 | 18.00 | 18.00 | 1.60 | 1.60 | 1.00 | 1.00 | 0.09 | 0.09 |
| *S. chamaedryfolia* | 5 | 11 | 20.00 | 20.00 | 20.00 | 1.00 | 1.00 | 1.00 | 1.00 | 0.05 | 0.05 |
| *S. chamaedryfolia* | 5 | 12 | 20.00 | 20.00 | 20.00 | 0.60 | 0.60 | 1.00 | 1.00 | 0.03 | 0.03 |
| *S. chamaedryfolia* | 5 | 13 | 20.00 | 20.00 | 18.00 | 0.60 | 0.60 | 1.00 | 0.90 | 0.03 | 0.03 |
| *S. chamaedryfolia* | 5 | 14 | 18.00 | 20.00 | 18.00 | 0.60 | 0.60 | 0.90 | 1.00 | 0.03 | 0.03 |
| *S. chamaedryfolia* | 5 | 15 | 20.00 | 20.00 | 18.00 | 0.60 | 0.60 | 1.00 | 0.90 | 0.03 | 0.03 |
| *S. chamaedryfolia* | 5 | 16 | 18.00 | 20.00 | 16.00 | 0.60 | 0.60 | 0.90 | 0.89 | 0.03 | 0.03 |
| *S. chamaedryfolia* | 5 | 17 | 18.00 | 20.00 | 18.00 | 0.60 | 0.60 | 0.90 | 1.00 | 0.03 | 0.03 |
| *S. chamaedryfolia* | 5 | 18 | 18.00 | 20.00 | 16.00 | 0.20 | 0.20 | 0.90 | 0.89 | 0.01 | 0.01 |
| *S. chamaedryfolia* | 5 | 19 | 18.00 | 20.00 | 16.00 | 0.60 | 0.60 | 0.90 | 0.89 | 0.03 | 0.03 |
| *S. chamaedryfolia* | 5 | 20 | 18.00 | 20.00 | 16.00 | 1.60 | 1.60 | 0.90 | 0.89 | 0.09 | 0.08 |
| *S. chamaedryfolia* | 5 | 21 | 18.00 | 20.00 | 18.00 | 0.60 | 0.60 | 0.90 | 1.00 | 0.03 | 0.03 |
| *S. chamaedryfolia* | 5 | 22 | 18.00 | 18.00 | 18.00 | 1.00 | 1.00 | 1.00 | 1.00 | 0.06 | 0.06 |
| *S. chamaedryfolia* | 5 | 23 | 18.00 | 20.00 | 18.00 | 0.60 | 0.60 | 0.90 | 1.00 | 0.03 | 0.03 |
| *S. chamaedryfolia* | 5 | 24 | 18.00 | 18.00 | 18.00 | 1.00 | 1.00 | 1.00 | 1.00 | 0.06 | 0.06 |
| *S. chamaedryfolia* | 5 | 25 | 20.00 | 20.00 | 16.00 | 0.60 | 0.60 | 1.00 | 0.80 | 0.03 | 0.03 |
| *S. chamaedryfolia* | 5 | 26 | 18.00 | 20.00 | 18.00 | 1.00 | 1.00 | 0.90 | 1.00 | 0.06 | 0.05 |
| *S. chamaedryfolia* | 5 | 27 | 18.00 | 20.00 | 18.00 | 1.60 | 1.60 | 0.90 | 1.00 | 0.09 | 0.08 |
| *S. chamaedryfolia* | 5 | 28 | 18.00 | 20.00 | 16.00 | 1.00 | 1.00 | 0.90 | 0.89 | 0.06 | 0.05 |
| *S. chamaedryfolia* | 5 | 29 | 18.00 | 20.00 | 18.00 | 1.00 | 1.00 | 0.90 | 1.00 | 0.06 | 0.05 |
| *S. chamaedryfolia* | 5 | 30 | 20.00 | 20.00 | 18.00 | 0.60 | 0.60 | 1.00 | 0.90 | 0.03 | 0.03 |
| *S. chinensis* | 6 | 1 | 16.00 | 14.00 | 16.00 | 0.60 | 0.60 | 1.14 | 1.00 | 0.04 | 0.04 |
| *S. chinensis* | 6 | 2 | 16.00 | 18.00 | 14.00 | 0.60 | 0.60 | 0.89 | 0.88 | 0.04 | 0.03 |
| *S. chinensis* | 6 | 3 | 16.00 | 16.00 | 12.00 | 0.60 | 0.60 | 1.00 | 0.75 | 0.04 | 0.04 |
| *S. chinensis* | 6 | 4 | 14.00 | 14.00 | 14.00 | 0.60 | 0.60 | 1.00 | 1.00 | 0.04 | 0.04 |
| *S. chinensis* | 6 | 5 | 16.00 | 14.00 | 16.00 | 0.60 | 0.60 | 1.14 | 1.00 | 0.04 | 0.04 |
| *S. chinensis* | 6 | 6 | 18.00 | 16.00 | 18.00 | 0.60 | 0.60 | 1.13 | 1.00 | 0.03 | 0.04 |
| *S. chinensis* | 6 | 7 | 16.00 | 16.00 | 14.00 | 0.60 | 0.60 | 1.00 | 0.88 | 0.04 | 0.04 |
| *S. chinensis* | 6 | 8 | 16.00 | 16.00 | 16.00 | 0.60 | 0.60 | 1.00 | 1.00 | 0.04 | 0.04 |
| *S. chinensis* | 6 | 9 | 14.00 | 14.00 | 12.00 | 0.60 | 0.60 | 1.00 | 0.86 | 0.04 | 0.04 |

| | | | | | | | | | | | |
|---|---|---|---|---|---|---|---|---|---|---|---|
| *S. chinensis* | 6 | 10 | 14.00 | 14.00 | 14.00 | 0.60 | 0.60 | 1.00 | 1.00 | 0.04 | 0.04 |
| *S. chinensis* | 6 | 11 | 16.00 | 16.00 | 16.00 | 1.00 | 1.00 | 1.00 | 1.00 | 0.06 | 0.06 |
| *S. chinensis* | 6 | 12 | 16.00 | 16.00 | 14.00 | 0.60 | 0.60 | 1.00 | 0.88 | 0.04 | 0.04 |
| *S. chinensis* | 6 | 13 | 16.00 | 16.00 | 14.00 | 0.60 | 0.60 | 1.00 | 0.88 | 0.04 | 0.04 |
| *S. chinensis* | 6 | 14 | 16.00 | 16.00 | 16.00 | 0.60 | 0.60 | 1.00 | 1.00 | 0.04 | 0.04 |
| *S. chinensis* | 6 | 15 | 16.00 | 16.00 | 12.00 | 1.00 | 1.00 | 1.00 | 0.75 | 0.06 | 0.06 |
| *S. chinensis* | 6 | 16 | 16.00 | 16.00 | 16.00 | 0.60 | 0.60 | 1.00 | 1.00 | 0.04 | 0.04 |
| *S. chinensis* | 6 | 17 | 14.00 | 14.00 | 14.00 | 0.60 | 0.60 | 1.00 | 1.00 | 0.04 | 0.04 |
| *S. chinensis* | 6 | 18 | 14.00 | 16.00 | 12.00 | 0.60 | 0.60 | 0.88 | 0.86 | 0.04 | 0.04 |
| *S. chinensis* | 6 | 19 | 18.00 | 16.00 | 18.00 | 0.60 | 0.60 | 1.13 | 1.00 | 0.03 | 0.04 |
| *S. chinensis* | 6 | 20 | 16.00 | 16.00 | 16.00 | 1.00 | 1.00 | 1.00 | 1.00 | 0.06 | 0.06 |
| *S. chinensis* | 6 | 21 | 16.00 | 16.00 | 16.00 | 0.60 | 0.60 | 1.00 | 1.00 | 0.04 | 0.04 |
| *S. chinensis* | 6 | 22 | 16.00 | 16.00 | 14.00 | 1.00 | 1.00 | 1.00 | 0.88 | 0.06 | 0.06 |
| *S. chinensis* | 6 | 23 | 16.00 | 16.00 | 14.00 | 1.00 | 1.00 | 1.00 | 0.88 | 0.06 | 0.06 |
| *S. chinensis* | 6 | 24 | 16.00 | 16.00 | 16.00 | 0.60 | 0.60 | 1.00 | 1.00 | 0.04 | 0.04 |
| *S. chinensis* | 6 | 25 | 18.00 | 16.00 | 18.00 | 0.60 | 0.60 | 1.13 | 1.00 | 0.03 | 0.04 |
| *S. chinensis* | 6 | 26 | 14.00 | 14.00 | 14.00 | 0.60 | 0.60 | 1.00 | 1.00 | 0.04 | 0.04 |
| *S. chinensis* | 6 | 27 | 16.00 | 16.00 | 16.00 | 1.00 | 1.00 | 1.00 | 1.00 | 0.06 | 0.06 |
| *S. chinensis* | 6 | 28 | 16.00 | 16.00 | 16.00 | 0.60 | 0.60 | 1.00 | 1.00 | 0.04 | 0.04 |
| *S. chinensis* | 6 | 29 | 16.00 | 16.00 | 16.00 | 0.20 | 0.20 | 1.00 | 1.00 | 0.01 | 0.01 |
| *S. chinensis* | 6 | 30 | 16.00 | 16.00 | 14.00 | 1.00 | 1.00 | 1.00 | 0.88 | 0.06 | 0.06 |
| *S. dasyantha* | 7 | 1 | 14.00 | 12.00 | 14.00 | 0.60 | 0.60 | 1.17 | 1.00 | 0.04 | 0.05 |
| *S. dasyantha* | 7 | 2 | 14.00 | 14.00 | 14.00 | 1.00 | 1.00 | 1.00 | 1.00 | 0.07 | 0.07 |
| *S. dasyantha* | 7 | 3 | 16.00 | 14.00 | 16.00 | 1.00 | 1.00 | 1.14 | 1.00 | 0.06 | 0.07 |
| *S. dasyantha* | 7 | 4 | 14.00 | 14.00 | 14.00 | 0.60 | 0.60 | 1.00 | 1.00 | 0.04 | 0.04 |
| *S. dasyantha* | 7 | 5 | 14.00 | 14.00 | 14.00 | 1.00 | 1.00 | 1.00 | 1.00 | 0.07 | 0.07 |
| *S. dasyantha* | 7 | 6 | 14.00 | 14.00 | 12.00 | 1.00 | 1.00 | 1.00 | 0.86 | 0.07 | 0.07 |
| *S. dasyantha* | 7 | 7 | 14.00 | 14.00 | 14.00 | 0.60 | 0.60 | 1.00 | 1.00 | 0.04 | 0.04 |
| *S. dasyantha* | 7 | 8 | 14.00 | 14.00 | 14.00 | 0.60 | 0.60 | 1.00 | 1.00 | 0.04 | 0.04 |
| *S. dasyantha* | 7 | 9 | 14.00 | 14.00 | 12.00 | 0.60 | 0.60 | 1.00 | 0.86 | 0.04 | 0.04 |
| *S. dasyantha* | 7 | 10 | 14.00 | 14.00 | 14.00 | 1.00 | 1.00 | 1.00 | 1.00 | 0.07 | 0.07 |
| *S. dasyantha* | 7 | 11 | 16.00 | 14.00 | 16.00 | 1.60 | 1.60 | 1.14 | 1.00 | 0.10 | 0.11 |
| *S. dasyantha* | 7 | 12 | 16.00 | 14.00 | 14.00 | 1.00 | 1.00 | 1.14 | 0.88 | 0.06 | 0.07 |
| *S. dasyantha* | 7 | 13 | 14.00 | 14.00 | 12.00 | 0.60 | 0.60 | 1.00 | 0.86 | 0.04 | 0.04 |
| *S. dasyantha* | 7 | 14 | 14.00 | 14.00 | 14.00 | 0.60 | 0.60 | 1.00 | 1.00 | 0.04 | 0.04 |
| *S. dasyantha* | 7 | 15 | 14.00 | 14.00 | 14.00 | 0.60 | 0.60 | 1.00 | 1.00 | 0.04 | 0.04 |
| *S. dasyantha* | 7 | 16 | 14.00 | 14.00 | 14.00 | 0.60 | 0.60 | 1.00 | 1.00 | 0.04 | 0.04 |
| *S. dasyantha* | 7 | 17 | 14.00 | 16.00 | 14.00 | 1.00 | 1.00 | 0.88 | 1.00 | 0.07 | 0.06 |
| *S. dasyantha* | 7 | 18 | 16.00 | 14.00 | 14.00 | 0.60 | 0.60 | 1.14 | 0.88 | 0.04 | 0.04 |
| *S. dasyantha* | 7 | 19 | 14.00 | 14.00 | 12.00 | 1.00 | 1.00 | 1.00 | 0.86 | 0.07 | 0.07 |
| *S. dasyantha* | 7 | 20 | 14.00 | 14.00 | 14.00 | 0.60 | 0.60 | 1.00 | 1.00 | 0.04 | 0.04 |
| *S. dasyantha* | 7 | 21 | 14.00 | 16.00 | 14.00 | 1.00 | 1.00 | 0.88 | 1.00 | 0.07 | 0.06 |
| *S. dasyantha* | 7 | 22 | 14.00 | 14.00 | 14.00 | 0.60 | 0.60 | 1.00 | 1.00 | 0.04 | 0.04 |
| *S. dasyantha* | 7 | 23 | 14.00 | 14.00 | 14.00 | 1.00 | 1.00 | 1.00 | 1.00 | 0.07 | 0.07 |
| *S. dasyantha* | 7 | 24 | 14.00 | 14.00 | 12.00 | 1.00 | 1.00 | 1.00 | 0.86 | 0.07 | 0.07 |
| *S. dasyantha* | 7 | 25 | 14.00 | 14.00 | 14.00 | 0.60 | 0.60 | 1.00 | 1.00 | 0.04 | 0.04 |
| *S. dasyantha* | 7 | 26 | 14.00 | 14.00 | 12.00 | 0.60 | 0.60 | 1.00 | 0.86 | 0.04 | 0.04 |
| *S. dasyantha* | 7 | 27 | 14.00 | 14.00 | 14.00 | 1.00 | 1.00 | 1.00 | 1.00 | 0.07 | 0.07 |
| *S. dasyantha* | 7 | 28 | 16.00 | 14.00 | 16.00 | 0.60 | 0.60 | 1.14 | 1.00 | 0.04 | 0.04 |
| *S. dasyantha* | 7 | 29 | 16.00 | 14.00 | 14.00 | 0.60 | 0.60 | 1.14 | 0.88 | 0.04 | 0.04 |
| *S. dasyantha* | 7 | 30 | 14.00 | 14.00 | 14.00 | 0.60 | 0.60 | 1.00 | 1.00 | 0.04 | 0.04 |
| *S. douglasii* | 8 | 1 | 14.00 | 14.00 | 12.00 | 0.60 | 0.60 | 1.00 | 0.86 | 0.04 | 0.04 |
| *S. douglasii* | 8 | 2 | 14.00 | 12.00 | 14.00 | 0.60 | 0.60 | 1.17 | 1.00 | 0.04 | 0.05 |
| *S. douglasii* | 8 | 3 | 14.00 | 12.00 | 14.00 | 1.00 | 1.00 | 1.17 | 1.00 | 0.07 | 0.08 |

| | | | | | | | | | | | |
|---|---|---|---|---|---|---|---|---|---|---|---|
| *S. douglasii* | 8 | 4 | 16.00 | 16.00 | 14.00 | 0.60 | 0.60 | 1.00 | 0.88 | 0.04 | 0.04 |
| *S. douglasii* | 8 | 5 | 14.00 | 14.00 | 12.00 | 0.60 | 0.60 | 1.00 | 0.86 | 0.04 | 0.04 |
| *S. douglasii* | 8 | 6 | 16.00 | 14.00 | 14.00 | 0.60 | 0.60 | 1.14 | 0.88 | 0.04 | 0.04 |
| *S. douglasii* | 8 | 7 | 14.00 | 14.00 | 14.00 | 1.00 | 1.00 | 1.00 | 1.00 | 0.07 | 0.07 |
| *S. douglasii* | 8 | 8 | 14.00 | 12.00 | 14.00 | 0.60 | 0.60 | 1.17 | 1.00 | 0.04 | 0.05 |
| *S. douglasii* | 8 | 9 | 14.00 | 14.00 | 14.00 | 0.60 | 0.60 | 1.00 | 1.00 | 0.04 | 0.04 |
| *S. douglasii* | 8 | 10 | 16.00 | 14.00 | 14.00 | 0.60 | 0.60 | 1.14 | 0.88 | 0.04 | 0.04 |
| *S. douglasii* | 8 | 11 | 14.00 | 16.00 | 14.00 | 1.00 | 1.00 | 0.88 | 1.00 | 0.07 | 0.06 |
| *S. douglasii* | 8 | 12 | 16.00 | 16.00 | 16.00 | 1.00 | 1.00 | 1.00 | 1.00 | 0.06 | 0.06 |
| *S. douglasii* | 8 | 13 | 18.00 | 16.00 | 16.00 | 0.60 | 0.60 | 1.13 | 0.89 | 0.03 | 0.04 |
| *S. douglasii* | 8 | 14 | 14.00 | 14.00 | 12.00 | 1.00 | 1.00 | 1.00 | 0.86 | 0.07 | 0.07 |
| *S. douglasii* | 8 | 15 | 14.00 | 14.00 | 12.00 | 0.60 | 0.60 | 1.00 | 0.86 | 0.04 | 0.04 |
| *S. douglasii* | 8 | 16 | 18.00 | 16.00 | 18.00 | 1.00 | 1.00 | 1.13 | 1.00 | 0.06 | 0.06 |
| *S. douglasii* | 8 | 17 | 18.00 | 16.00 | 16.00 | 1.60 | 1.60 | 1.13 | 0.89 | 0.09 | 0.10 |
| *S. douglasii* | 8 | 18 | 16.00 | 14.00 | 16.00 | 1.00 | 1.00 | 1.14 | 1.00 | 0.06 | 0.07 |
| *S. douglasii* | 8 | 19 | 18.00 | 14.00 | 16.00 | 0.60 | 0.60 | 1.29 | 0.89 | 0.03 | 0.04 |
| *S. douglasii* | 8 | 20 | 16.00 | 14.00 | 16.00 | 0.60 | 0.60 | 1.14 | 1.00 | 0.04 | 0.04 |
| *S. douglasii* | 8 | 21 | 14.00 | 14.00 | 12.00 | 1.00 | 1.00 | 1.00 | 0.86 | 0.07 | 0.07 |
| *S. douglasii* | 8 | 22 | 14.00 | 14.00 | 14.00 | 1.60 | 1.60 | 1.00 | 1.00 | 0.11 | 0.11 |
| *S. douglasii* | 8 | 23 | 14.00 | 14.00 | 12.00 | 1.00 | 1.00 | 1.00 | 0.86 | 0.07 | 0.07 |
| *S. douglasii* | 8 | 24 | 16.00 | 16.00 | 16.00 | 0.60 | 0.60 | 1.00 | 1.00 | 0.04 | 0.04 |
| *S. douglasii* | 8 | 25 | 16.00 | 16.00 | 14.00 | 1.00 | 1.00 | 1.00 | 0.88 | 0.06 | 0.06 |
| *S. douglasii* | 8 | 26 | 16.00 | 16.00 | 16.00 | 0.60 | 0.60 | 1.00 | 1.00 | 0.04 | 0.04 |
| *S. douglasii* | 8 | 27 | 16.00 | 14.00 | 16.00 | 1.00 | 1.00 | 1.14 | 1.00 | 0.06 | 0.07 |
| *S. douglasii* | 8 | 28 | 18.00 | 16.00 | 18.00 | 1.00 | 1.00 | 1.13 | 1.00 | 0.06 | 0.06 |
| *S. douglasii* | 8 | 29 | 16.00 | 14.00 | 16.00 | 1.00 | 1.00 | 1.14 | 1.00 | 0.06 | 0.07 |
| *S. douglasii* | 8 | 30 | 14.00 | 14.00 | 12.00 | 1.00 | 1.00 | 1.00 | 0.86 | 0.07 | 0.07 |
| *S. elegans* | 9 | 1 | 14.00 | 16.00 | 12.00 | 1.60 | 1.60 | 0.88 | 0.86 | 0.11 | 0.10 |
| *S. elegans* | 9 | 2 | 14.00 | 14.00 | 14.00 | 1.00 | 1.00 | 1.00 | 1.00 | 0.07 | 0.07 |
| *S. elegans* | 9 | 3 | 14.00 | 16.00 | 14.00 | 1.00 | 1.00 | 0.88 | 1.00 | 0.07 | 0.06 |
| *S. elegans* | 9 | 4 | 16.00 | 16.00 | 14.00 | 1.60 | 1.60 | 1.00 | 0.88 | 0.10 | 0.10 |
| *S. elegans* | 9 | 5 | 16.00 | 16.00 | 16.00 | 1.00 | 1.00 | 1.00 | 1.00 | 0.06 | 0.06 |
| *S. elegans* | 9 | 6 | 16.00 | 16.00 | 14.00 | 1.00 | 1.00 | 1.00 | 0.88 | 0.06 | 0.06 |
| *S. elegans* | 9 | 7 | 14.00 | 16.00 | 14.00 | 1.00 | 1.00 | 0.88 | 1.00 | 0.07 | 0.06 |
| *S. elegans* | 9 | 8 | 16.00 | 16.00 | 16.00 | 1.00 | 1.00 | 1.00 | 1.00 | 0.06 | 0.06 |
| *S. elegans* | 9 | 9 | 16.00 | 16.00 | 14.00 | 1.00 | 1.00 | 1.00 | 0.88 | 0.06 | 0.06 |
| *S. elegans* | 9 | 10 | 16.00 | 16.00 | 16.00 | 1.00 | 1.00 | 1.00 | 1.00 | 0.06 | 0.06 |
| *S. elegans* | 9 | 11 | 14.00 | 16.00 | 14.00 | 0.60 | 0.60 | 0.88 | 1.00 | 0.04 | 0.04 |
| *S. elegans* | 9 | 12 | 16.00 | 16.00 | 16.00 | 1.60 | 1.60 | 1.00 | 1.00 | 0.10 | 0.10 |
| *S. elegans* | 9 | 13 | 16.00 | 14.00 | 16.00 | 1.00 | 1.00 | 1.14 | 1.00 | 0.06 | 0.07 |
| *S. elegans* | 9 | 14 | 16.00 | 16.00 | 16.00 | 1.00 | 1.00 | 1.00 | 1.00 | 0.06 | 0.06 |
| *S. elegans* | 9 | 15 | 16.00 | 16.00 | 14.00 | 1.60 | 1.60 | 1.00 | 0.88 | 0.10 | 0.10 |
| *S. elegans* | 9 | 16 | 14.00 | 14.00 | 14.00 | 1.00 | 1.00 | 1.00 | 1.00 | 0.07 | 0.07 |
| *S. elegans* | 9 | 17 | 16.00 | 16.00 | 16.00 | 0.60 | 0.60 | 1.00 | 1.00 | 0.04 | 0.04 |
| *S. elegans* | 9 | 18 | 16.00 | 16.00 | 16.00 | 1.00 | 1.00 | 1.00 | 1.00 | 0.06 | 0.06 |
| *S. elegans* | 9 | 19 | 16.00 | 14.00 | 16.00 | 1.00 | 1.00 | 1.14 | 1.00 | 0.06 | 0.07 |
| *S. elegans* | 9 | 20 | 16.00 | 16.00 | 16.00 | 1.00 | 1.00 | 1.00 | 1.00 | 0.06 | 0.06 |
| *S. elegans* | 9 | 21 | 16.00 | 16.00 | 16.00 | 1.60 | 1.60 | 1.00 | 1.00 | 0.10 | 0.10 |
| *S. elegans* | 9 | 22 | 16.00 | 16.00 | 16.00 | 0.60 | 0.60 | 1.00 | 1.00 | 0.04 | 0.04 |
| *S. elegans* | 9 | 23 | 16.00 | 16.00 | 14.00 | 1.00 | 1.00 | 1.00 | 0.88 | 0.06 | 0.06 |
| *S. elegans* | 9 | 24 | 16.00 | 16.00 | 16.00 | 1.00 | 1.00 | 1.00 | 1.00 | 0.06 | 0.06 |
| *S. elegans* | 9 | 25 | 16.00 | 16.00 | 16.00 | 0.60 | 0.60 | 1.00 | 1.00 | 0.04 | 0.04 |
| *S. elegans* | 9 | 26 | 16.00 | 16.00 | 14.00 | 1.00 | 1.00 | 1.00 | 0.88 | 0.06 | 0.06 |
| *S. elegans* | 9 | 27 | 16.00 | 16.00 | 16.00 | 0.60 | 0.60 | 1.00 | 1.00 | 0.04 | 0.04 |

| | | | | | | | | | | | |
|---|---|---|---|---|---|---|---|---|---|---|---|
| *S. elegans* | 9 | 28 | 16.00 | 16.00 | 14.00 | 1.00 | 1.00 | 1.00 | 0.88 | 0.06 | 0.06 |
| *S. elegans* | 9 | 29 | 16.00 | 16.00 | 14.00 | 1.60 | 1.60 | 1.00 | 0.88 | 0.10 | 0.10 |
| *S. elegans* | 9 | 30 | 16.00 | 16.00 | 16.00 | 1.00 | 1.00 | 1.00 | 1.00 | 0.06 | 0.06 |
| *S. henryi* | 10 | 1 | 14.00 | 14.00 | 14.00 | 1.00 | 1.00 | 1.00 | 1.00 | 0.07 | 0.07 |
| *S. henryi* | 10 | 2 | 14.00 | 14.00 | 12.00 | 0.60 | 0.60 | 1.00 | 0.86 | 0.04 | 0.04 |
| *S. henryi* | 10 | 3 | 14.00 | 16.00 | 12.00 | 1.00 | 1.00 | 0.88 | 0.86 | 0.07 | 0.06 |
| *S. henryi* | 10 | 4 | 16.00 | 16.00 | 12.00 | 0.60 | 0.60 | 1.00 | 0.75 | 0.04 | 0.04 |
| *S. henryi* | 10 | 5 | 16.00 | 16.00 | 14.00 | 0.60 | 0.60 | 1.00 | 0.88 | 0.04 | 0.04 |
| *S. henryi* | 10 | 6 | 14.00 | 14.00 | 14.00 | 0.60 | 0.60 | 1.00 | 1.00 | 0.04 | 0.04 |
| *S. henryi* | 10 | 7 | 16.00 | 16.00 | 16.00 | 0.60 | 0.60 | 1.00 | 1.00 | 0.04 | 0.04 |
| *S. henryi* | 10 | 8 | 16.00 | 16.00 | 14.00 | 0.60 | 0.60 | 1.00 | 0.88 | 0.04 | 0.04 |
| *S. henryi* | 10 | 9 | 14.00 | 14.00 | 14.00 | 0.60 | 0.60 | 1.00 | 1.00 | 0.04 | 0.04 |
| *S. henryi* | 10 | 10 | 16.00 | 14.00 | 12.00 | 1.00 | 1.00 | 1.14 | 0.75 | 0.06 | 0.07 |
| *S. henryi* | 10 | 11 | 14.00 | 14.00 | 14.00 | 1.60 | 1.60 | 1.00 | 1.00 | 0.11 | 0.11 |
| *S. henryi* | 10 | 12 | 14.00 | 16.00 | 14.00 | 0.60 | 0.60 | 0.88 | 1.00 | 0.04 | 0.04 |
| *S. henryi* | 10 | 13 | 14.00 | 14.00 | 12.00 | 0.60 | 0.60 | 1.00 | 0.86 | 0.04 | 0.04 |
| *S. henryi* | 10 | 14 | 16.00 | 16.00 | 14.00 | 1.00 | 1.00 | 1.00 | 0.88 | 0.06 | 0.06 |
| *S. henryi* | 10 | 15 | 16.00 | 16.00 | 16.00 | 1.00 | 1.00 | 1.00 | 1.00 | 0.06 | 0.06 |
| *S. henryi* | 10 | 16 | 16.00 | 14.00 | 16.00 | 1.00 | 1.00 | 1.14 | 1.00 | 0.06 | 0.07 |
| *S. henryi* | 10 | 17 | 16.00 | 16.00 | 14.00 | 0.60 | 0.60 | 1.00 | 0.88 | 0.04 | 0.04 |
| *S. henryi* | 10 | 18 | 16.00 | 16.00 | 16.00 | 1.60 | 1.60 | 1.00 | 1.00 | 0.10 | 0.10 |
| *S. henryi* | 10 | 19 | 16.00 | 16.00 | 14.00 | 1.00 | 1.00 | 1.00 | 0.88 | 0.06 | 0.06 |
| *S. henryi* | 10 | 20 | 16.00 | 14.00 | 16.00 | 1.60 | 1.60 | 1.14 | 1.00 | 0.10 | 0.11 |
| *S. henryi* | 10 | 21 | 16.00 | 14.00 | 14.00 | 1.00 | 1.00 | 1.14 | 0.88 | 0.06 | 0.07 |
| *S. henryi* | 10 | 22 | 16.00 | 16.00 | 14.00 | 0.60 | 0.60 | 1.00 | 0.88 | 0.04 | 0.04 |
| *S. henryi* | 10 | 23 | 16.00 | 14.00 | 16.00 | 2.00 | 2.00 | 1.14 | 1.00 | 0.13 | 0.14 |
| *S. henryi* | 10 | 24 | 14.00 | 14.00 | 14.00 | 1.00 | 1.00 | 1.00 | 1.00 | 0.07 | 0.07 |
| *S. henryi* | 10 | 25 | 16.00 | 16.00 | 16.00 | 1.60 | 1.60 | 1.00 | 1.00 | 0.10 | 0.10 |
| *S. henryi* | 10 | 26 | 14.00 | 14.00 | 14.00 | 1.00 | 1.00 | 1.00 | 1.00 | 0.07 | 0.07 |
| *S. henryi* | 10 | 27 | 14.00 | 14.00 | 12.00 | 1.00 | 1.00 | 1.00 | 0.86 | 0.07 | 0.07 |
| *S. henryi* | 10 | 28 | 14.00 | 14.00 | 14.00 | 0.60 | 0.60 | 1.00 | 1.00 | 0.04 | 0.04 |
| *S. henryi* | 10 | 29 | 16.00 | 16.00 | 16.00 | 1.00 | 1.00 | 1.00 | 1.00 | 0.06 | 0.06 |
| *S. henryi* | 10 | 30 | 16.00 | 14.00 | 16.00 | 1.60 | 1.60 | 1.14 | 1.00 | 0.10 | 0.11 |
| *S. hypericifolia* | 11 | 1 | 16.00 | 20.00 | 16.00 | 1.00 | 1.00 | 0.80 | 1.00 | 0.06 | 0.05 |
| *S. hypericifolia* | 11 | 2 | 20.00 | 20.00 | 20.00 | 1.00 | 1.00 | 1.00 | 1.00 | 0.05 | 0.05 |
| *S. hypericifolia* | 11 | 3 | 20.00 | 20.00 | 20.00 | 1.00 | 1.00 | 1.00 | 1.00 | 0.05 | 0.05 |
| *S. hypericifolia* | 11 | 4 | 18.00 | 18.00 | 17.00 | 1.60 | 1.60 | 1.00 | 0.94 | 0.09 | 0.09 |
| *S. hypericifolia* | 11 | 5 | 18.00 | 20.00 | 18.00 | 0.60 | 0.60 | 0.90 | 1.00 | 0.03 | 0.03 |
| *S. hypericifolia* | 11 | 6 | 18.00 | 16.00 | 16.00 | 1.00 | 1.00 | 1.13 | 0.89 | 0.06 | 0.06 |
| *S. hypericifolia* | 11 | 7 | 20.00 | 20.00 | 16.00 | 1.60 | 1.60 | 1.00 | 0.80 | 0.08 | 0.08 |
| *S. hypericifolia* | 11 | 8 | 18.00 | 16.00 | 18.00 | 0.60 | 0.60 | 1.13 | 1.00 | 0.03 | 0.04 |
| *S. hypericifolia* | 11 | 9 | 18.00 | 20.00 | 18.00 | 0.20 | 0.20 | 0.90 | 1.00 | 0.01 | 0.01 |
| *S. hypericifolia* | 11 | 10 | 18.00 | 20.00 | 16.00 | 0.60 | 0.60 | 0.90 | 0.89 | 0.03 | 0.03 |
| *S. hypericifolia* | 11 | 11 | 16.00 | 18.00 | 16.00 | 1.00 | 1.00 | 0.89 | 1.00 | 0.06 | 0.06 |
| *S. hypericifolia* | 11 | 12 | 18.00 | 20.00 | 18.00 | 1.00 | 1.00 | 0.90 | 1.00 | 0.06 | 0.05 |
| *S. hypericifolia* | 11 | 13 | 20.00 | 20.00 | 20.00 | 1.60 | 1.60 | 1.00 | 1.00 | 0.08 | 0.08 |
| *S. hypericifolia* | 11 | 14 | 18.00 | 20.00 | 18.00 | 0.60 | 0.60 | 0.90 | 1.00 | 0.03 | 0.03 |
| *S. hypericifolia* | 11 | 15 | 20.00 | 20.00 | 20.00 | 1.00 | 1.00 | 1.00 | 1.00 | 0.05 | 0.05 |
| *S. hypericifolia* | 11 | 16 | 18.00 | 20.00 | 16.00 | 1.00 | 1.00 | 0.90 | 0.89 | 0.06 | 0.05 |
| *S. hypericifolia* | 11 | 17 | 16.00 | 18.00 | 16.00 | 1.00 | 1.00 | 0.89 | 1.00 | 0.06 | 0.06 |
| *S. hypericifolia* | 11 | 18 | 20.00 | 20.00 | 20.00 | 1.00 | 1.00 | 1.00 | 1.00 | 0.05 | 0.05 |
| *S. hypericifolia* | 11 | 19 | 18.00 | 18.00 | 18.00 | 1.00 | 1.00 | 1.00 | 1.00 | 0.06 | 0.06 |
| *S. hypericifolia* | 11 | 20 | 20.00 | 20.00 | 16.00 | 1.00 | 1.00 | 1.00 | 0.80 | 0.05 | 0.05 |
| *S. hypericifolia* | 11 | 21 | 18.00 | 20.00 | 18.00 | 0.60 | 0.60 | 0.90 | 1.00 | 0.03 | 0.03 |

| | | | | | | | | | | | |
|---|---|---|---|---|---|---|---|---|---|---|---|
| *S. hypericifolia* | 11 | 22 | 20.00 | 20.00 | 20.00 | 0.20 | 0.20 | 1.00 | 1.00 | 0.01 | 0.01 |
| *S. hypericifolia* | 11 | 23 | 18.00 | 22.00 | 16.00 | 1.00 | 1.00 | 0.82 | 0.89 | 0.06 | 0.05 |
| *S. hypericifolia* | 11 | 24 | 18.00 | 20.00 | 18.00 | 1.00 | 1.00 | 0.90 | 1.00 | 0.06 | 0.05 |
| *S. hypericifolia* | 11 | 25 | 18.00 | 18.00 | 18.00 | 0.60 | 0.60 | 1.00 | 1.00 | 0.03 | 0.03 |
| *S. hypericifolia* | 11 | 26 | 18.00 | 18.00 | 16.00 | 0.20 | 0.20 | 1.00 | 0.89 | 0.01 | 0.01 |
| *S. hypericifolia* | 11 | 27 | 18.00 | 18.00 | 18.00 | 0.60 | 0.60 | 1.00 | 1.00 | 0.03 | 0.03 |
| *S. hypericifolia* | 11 | 28 | 18.00 | 18.00 | 16.00 | 0.60 | 0.60 | 1.00 | 0.89 | 0.03 | 0.03 |
| *S. hypericifolia* | 11 | 29 | 16.00 | 18.00 | 16.00 | 1.00 | 1.00 | 0.89 | 1.00 | 0.06 | 0.06 |
| *S. hypericifolia* | 11 | 30 | 18.00 | 18.00 | 16.00 | 1.00 | 1.00 | 1.00 | 0.89 | 0.06 | 0.06 |
| *S. japonica* | 12 | 1 | 16.00 | 14.00 | 12.00 | 1.60 | 1.60 | 1.14 | 0.75 | 0.10 | 0.11 |
| *S. japonica* | 12 | 2 | 14.00 | 14.00 | 14.00 | 1.60 | 1.60 | 1.00 | 1.00 | 0.11 | 0.11 |
| *S. japonica* | 12 | 3 | 16.00 | 14.00 | 12.00 | 1.00 | 1.00 | 1.14 | 0.75 | 0.06 | 0.07 |
| *S. japonica* | 12 | 4 | 16.00 | 14.00 | 14.00 | 1.00 | 1.00 | 1.14 | 0.88 | 0.06 | 0.07 |
| *S. japonica* | 12 | 5 | 16.00 | 16.00 | 16.00 | 1.60 | 1.60 | 1.00 | 1.00 | 0.10 | 0.10 |
| *S. japonica* | 12 | 6 | 16.00 | 16.00 | 14.00 | 1.60 | 1.60 | 1.00 | 0.88 | 0.10 | 0.10 |
| *S. japonica* | 12 | 7 | 14.00 | 14.00 | 14.00 | 1.00 | 1.00 | 1.00 | 1.00 | 0.07 | 0.07 |
| *S. japonica* | 12 | 8 | 14.00 | 14.00 | 12.00 | 1.00 | 1.00 | 1.00 | 0.86 | 0.07 | 0.07 |
| *S. japonica* | 12 | 9 | 16.00 | 16.00 | 16.00 | 1.00 | 1.00 | 1.00 | 1.00 | 0.06 | 0.06 |
| *S. japonica* | 12 | 10 | 16.00 | 16.00 | 16.00 | 1.00 | 1.00 | 1.00 | 1.00 | 0.06 | 0.06 |
| *S. japonica* | 12 | 11 | 16.00 | 16.00 | 14.00 | 1.00 | 1.00 | 1.00 | 0.88 | 0.06 | 0.06 |
| *S. japonica* | 12 | 12 | 14.00 | 14.00 | 14.00 | 1.00 | 1.00 | 1.00 | 1.00 | 0.07 | 0.07 |
| *S. japonica* | 12 | 13 | 16.00 | 16.00 | 14.00 | 1.00 | 1.00 | 1.00 | 0.88 | 0.06 | 0.06 |
| *S. japonica* | 12 | 14 | 16.00 | 16.00 | 12.00 | 1.00 | 1.00 | 1.00 | 0.75 | 0.06 | 0.06 |
| *S. japonica* | 12 | 15 | 18.00 | 16.00 | 16.00 | 0.60 | 0.60 | 1.13 | 0.89 | 0.03 | 0.04 |
| *S. japonica* | 12 | 16 | 16.00 | 16.00 | 14.00 | 0.60 | 0.60 | 1.00 | 0.88 | 0.04 | 0.04 |
| *S. japonica* | 12 | 17 | 16.00 | 14.00 | 14.00 | 0.60 | 0.60 | 1.14 | 0.88 | 0.04 | 0.04 |
| *S. japonica* | 12 | 18 | 16.00 | 14.00 | 12.00 | 1.00 | 1.00 | 1.14 | 0.75 | 0.06 | 0.07 |
| *S. japonica* | 12 | 19 | 16.00 | 14.00 | 14.00 | 0.60 | 0.60 | 1.14 | 0.88 | 0.04 | 0.04 |
| *S. japonica* | 12 | 20 | 16.00 | 14.00 | 14.00 | 1.00 | 1.00 | 1.14 | 0.88 | 0.06 | 0.07 |
| *S. japonica* | 12 | 21 | 14.00 | 14.00 | 14.00 | 1.00 | 1.00 | 1.00 | 1.00 | 0.07 | 0.07 |
| *S. japonica* | 12 | 22 | 14.00 | 14.00 | 12.00 | 1.00 | 1.00 | 1.00 | 0.86 | 0.07 | 0.07 |
| *S. japonica* | 12 | 23 | 16.00 | 14.00 | 16.00 | 1.00 | 1.00 | 1.14 | 1.00 | 0.06 | 0.07 |
| *S. japonica* | 12 | 24 | 16.00 | 16.00 | 16.00 | 1.00 | 1.00 | 1.00 | 1.00 | 0.06 | 0.06 |
| *S. japonica* | 12 | 25 | 16.00 | 16.00 | 14.00 | 1.00 | 1.00 | 1.00 | 0.88 | 0.06 | 0.06 |
| *S. japonica* | 12 | 26 | 16.00 | 14.00 | 16.00 | 1.00 | 1.00 | 1.14 | 1.00 | 0.06 | 0.07 |
| *S. japonica* | 12 | 27 | 14.00 | 16.00 | 14.00 | 1.00 | 1.00 | 0.88 | 1.00 | 0.07 | 0.06 |
| *S. japonica* | 12 | 28 | 16.00 | 14.00 | 14.00 | 0.60 | 0.60 | 1.14 | 0.88 | 0.04 | 0.04 |
| *S. japonica* | 12 | 29 | 16.00 | 16.00 | 16.00 | 1.00 | 1.00 | 1.00 | 1.00 | 0.06 | 0.06 |
| *S. japonica* | 12 | 30 | 16.00 | 14.00 | 12.00 | 1.00 | 1.00 | 1.14 | 0.75 | 0.06 | 0.07 |
| *S. media* | 13 | 1 | 16.00 | 16.00 | 16.00 | 0.60 | 0.60 | 1.00 | 1.00 | 0.04 | 0.04 |
| *S. media* | 13 | 2 | 16.00 | 16.00 | 16.00 | 1.00 | 1.00 | 1.00 | 1.00 | 0.06 | 0.06 |
| *S. media* | 13 | 3 | 16.00 | 14.00 | 16.00 | 1.60 | 1.60 | 1.14 | 1.00 | 0.10 | 0.11 |
| *S. media* | 13 | 4 | 16.00 | 16.00 | 16.00 | 1.00 | 1.00 | 1.00 | 1.00 | 0.06 | 0.06 |
| *S. media* | 13 | 5 | 14.00 | 14.00 | 12.00 | 1.00 | 1.00 | 1.00 | 0.86 | 0.07 | 0.07 |
| *S. media* | 13 | 6 | 16.00 | 16.00 | 14.00 | 0.60 | 0.60 | 1.00 | 0.88 | 0.04 | 0.04 |
| *S. media* | 13 | 7 | 16.00 | 16.00 | 16.00 | 0.60 | 0.60 | 1.00 | 1.00 | 0.04 | 0.04 |
| *S. media* | 13 | 8 | 16.00 | 16.00 | 14.00 | 1.00 | 1.00 | 1.00 | 0.88 | 0.06 | 0.06 |
| *S. media* | 13 | 9 | 14.00 | 16.00 | 14.00 | 1.00 | 1.00 | 0.88 | 1.00 | 0.07 | 0.06 |
| *S. media* | 13 | 10 | 16.00 | 16.00 | 16.00 | 1.00 | 1.00 | 1.00 | 1.00 | 0.06 | 0.06 |
| *S. media* | 13 | 11 | 18.00 | 18.00 | 16.00 | 1.60 | 1.60 | 1.00 | 0.89 | 0.09 | 0.09 |
| *S. media* | 13 | 12 | 18.00 | 18.00 | 16.00 | 0.60 | 0.60 | 1.00 | 0.89 | 0.03 | 0.03 |
| *S. media* | 13 | 13 | 14.00 | 16.00 | 12.00 | 1.00 | 1.00 | 0.88 | 0.86 | 0.07 | 0.06 |
| *S. media* | 13 | 14 | 16.00 | 16.00 | 14.00 | 1.00 | 1.00 | 1.00 | 0.88 | 0.06 | 0.06 |
| *S. media* | 13 | 15 | 16.00 | 16.00 | 14.00 | 1.60 | 1.60 | 1.00 | 0.88 | 0.10 | 0.10 |

| | | | | | | | | | | |
|---|---|---|---|---|---|---|---|---|---|---|
| *S. media* | 13 | 16 | 16.00 | 16.00 | 14.00 | 1.00 | 1.00 | 1.00 | 0.88 | 0.06 | 0.06 |
| *S. media* | 13 | 17 | 16.00 | 16.00 | 16.00 | 1.00 | 1.00 | 1.00 | 1.00 | 0.06 | 0.06 |
| *S. media* | 13 | 18 | 14.00 | 16.00 | 12.00 | 0.60 | 0.60 | 0.88 | 0.86 | 0.04 | 0.04 |
| *S. media* | 13 | 19 | 14.00 | 14.00 | 12.00 | 0.60 | 0.60 | 1.00 | 0.86 | 0.04 | 0.04 |
| *S. media* | 13 | 20 | 14.00 | 18.00 | 12.00 | 1.00 | 1.00 | 0.78 | 0.86 | 0.07 | 0.06 |
| *S. media* | 13 | 21 | 18.00 | 14.00 | 18.00 | 1.00 | 1.00 | 1.29 | 1.00 | 0.06 | 0.07 |
| *S. media* | 13 | 22 | 14.00 | 16.00 | 12.00 | 1.00 | 1.00 | 0.88 | 0.86 | 0.07 | 0.06 |
| *S. media* | 13 | 23 | 16.00 | 14.00 | 16.00 | 1.00 | 1.00 | 1.14 | 1.00 | 0.06 | 0.07 |
| *S. media* | 13 | 24 | 16.00 | 16.00 | 16.00 | 1.00 | 1.00 | 1.00 | 1.00 | 0.06 | 0.06 |
| *S. media* | 13 | 25 | 16.00 | 16.00 | 14.00 | 1.00 | 1.00 | 1.00 | 0.88 | 0.06 | 0.06 |
| *S. media* | 13 | 26 | 16.00 | 16.00 | 16.00 | 1.00 | 1.00 | 1.00 | 1.00 | 0.06 | 0.06 |
| *S. media* | 13 | 27 | 16.00 | 16.00 | 16.00 | 1.60 | 1.60 | 1.00 | 1.00 | 0.10 | 0.10 |
| *S. media* | 13 | 28 | 16.00 | 14.00 | 16.00 | 0.60 | 0.60 | 1.14 | 1.00 | 0.04 | 0.04 |
| *S. media* | 13 | 29 | 14.00 | 16.00 | 14.00 | 1.00 | 1.00 | 0.88 | 1.00 | 0.07 | 0.06 |
| *S. media* | 13 | 30 | 16.00 | 16.00 | 14.00 | 1.00 | 1.00 | 1.00 | 0.88 | 0.06 | 0.06 |
| *S. nipponica* | 14 | 1 | 22.00 | 20.00 | 22.00 | 2.00 | 2.00 | 1.10 | 1.00 | 0.09 | 0.10 |
| *S. nipponica* | 14 | 2 | 16.00 | 16.00 | 16.00 | 2.00 | 2.00 | 1.00 | 1.00 | 0.13 | 0.13 |
| *S. nipponica* | 14 | 3 | 16.00 | 16.00 | 16.00 | 2.00 | 2.00 | 1.00 | 1.00 | 0.13 | 0.13 |
| *S. nipponica* | 14 | 4 | 16.00 | 16.00 | 14.00 | 2.00 | 2.00 | 1.00 | 0.88 | 0.13 | 0.13 |
| *S. nipponica* | 14 | 5 | 16.00 | 16.00 | 14.00 | 1.00 | 1.00 | 1.00 | 0.88 | 0.06 | 0.06 |
| *S. nipponica* | 14 | 6 | 18.00 | 16.00 | 16.00 | 2.00 | 2.00 | 1.13 | 0.89 | 0.11 | 0.13 |
| *S. nipponica* | 14 | 7 | 16.00 | 14.00 | 14.00 | 1.00 | 1.00 | 1.14 | 0.88 | 0.06 | 0.07 |
| *S. nipponica* | 14 | 8 | 16.00 | 14.00 | 14.00 | 1.00 | 1.00 | 1.14 | 0.88 | 0.06 | 0.07 |
| *S. nipponica* | 14 | 9 | 16.00 | 16.00 | 14.00 | 1.00 | 1.00 | 1.00 | 0.88 | 0.06 | 0.06 |
| *S. nipponica* | 14 | 10 | 16.00 | 14.00 | 14.00 | 1.00 | 1.00 | 1.14 | 0.88 | 0.06 | 0.07 |
| *S. nipponica* | 14 | 11 | 16.00 | 16.00 | 14.00 | 1.60 | 1.60 | 1.00 | 0.88 | 0.10 | 0.10 |
| *S. nipponica* | 14 | 12 | 16.00 | 14.00 | 14.00 | 1.60 | 1.60 | 1.14 | 0.88 | 0.10 | 0.11 |
| *S. nipponica* | 14 | 13 | 16.00 | 16.00 | 14.00 | 1.00 | 1.00 | 1.00 | 0.88 | 0.06 | 0.06 |
| *S. nipponica* | 14 | 14 | 14.00 | 14.00 | 12.00 | 0.60 | 0.60 | 1.00 | 0.86 | 0.04 | 0.04 |
| *S. nipponica* | 14 | 15 | 16.00 | 16.00 | 14.00 | 1.00 | 1.00 | 1.00 | 0.88 | 0.06 | 0.06 |
| *S. nipponica* | 14 | 16 | 16.00 | 18.00 | 16.00 | 1.00 | 1.00 | 0.89 | 1.00 | 0.06 | 0.06 |
| *S. nipponica* | 14 | 17 | 16.00 | 14.00 | 14.00 | 1.60 | 1.60 | 1.14 | 0.88 | 0.10 | 0.11 |
| *S. nipponica* | 14 | 18 | 16.00 | 18.00 | 14.00 | 1.00 | 1.00 | 0.89 | 0.88 | 0.06 | 0.06 |
| *S. nipponica* | 14 | 19 | 16.00 | 14.00 | 14.00 | 1.00 | 1.00 | 1.14 | 0.88 | 0.06 | 0.07 |
| *S. nipponica* | 14 | 20 | 18.00 | 16.00 | 14.00 | 1.60 | 1.60 | 1.13 | 0.78 | 0.09 | 0.10 |
| *S. nipponica* | 14 | 21 | 16.00 | 14.00 | 16.00 | 1.00 | 1.00 | 1.14 | 1.00 | 0.06 | 0.07 |
| *S. nipponica* | 14 | 22 | 16.00 | 14.00 | 14.00 | 1.60 | 1.60 | 1.14 | 0.88 | 0.10 | 0.11 |
| *S. nipponica* | 14 | 23 | 18.00 | 14.00 | 14.00 | 1.60 | 1.60 | 1.29 | 0.78 | 0.09 | 0.11 |
| *S. nipponica* | 14 | 24 | 18.00 | 14.00 | 18.00 | 1.00 | 1.00 | 1.29 | 1.00 | 0.06 | 0.07 |
| *S. nipponica* | 14 | 25 | 18.00 | 18.00 | 16.00 | 1.00 | 1.00 | 1.00 | 0.89 | 0.06 | 0.06 |
| *S. nipponica* | 14 | 26 | 16.00 | 16.00 | 14.00 | 1.00 | 1.00 | 1.00 | 0.88 | 0.06 | 0.06 |
| *S. nipponica* | 14 | 27 | 16.00 | 16.00 | 14.00 | 1.00 | 1.00 | 1.00 | 0.88 | 0.06 | 0.06 |
| *S. nipponica* | 14 | 28 | 18.00 | 18.00 | 16.00 | 1.00 | 1.00 | 1.00 | 0.89 | 0.06 | 0.06 |
| *S. nipponica* | 14 | 29 | 18.00 | 16.00 | 16.00 | 1.60 | 1.60 | 1.13 | 0.89 | 0.09 | 0.10 |
| *S. nipponica* | 14 | 30 | 16.00 | 16.00 | 16.00 | 1.60 | 1.60 | 1.00 | 1.00 | 0.10 | 0.10 |
| *S. pubescens* | 15 | 1 | 14.00 | 14.00 | 12.00 | 1.00 | 1.00 | 1.00 | 0.86 | 0.07 | 0.07 |
| *S. pubescens* | 15 | 2 | 16.00 | 16.00 | 16.00 | 0.20 | 0.20 | 1.00 | 1.00 | 0.01 | 0.01 |
| *S. pubescens* | 15 | 3 | 14.00 | 12.00 | 14.00 | 1.00 | 1.00 | 1.17 | 1.00 | 0.07 | 0.08 |
| *S. pubescens* | 15 | 4 | 14.00 | 14.00 | 14.00 | 1.00 | 1.00 | 1.00 | 1.00 | 0.07 | 0.07 |
| *S. pubescens* | 15 | 5 | 16.00 | 14.00 | 14.00 | 1.00 | 1.00 | 1.14 | 0.88 | 0.06 | 0.07 |
| *S. pubescens* | 15 | 6 | 14.00 | 14.00 | 12.00 | 1.00 | 1.00 | 1.00 | 0.86 | 0.07 | 0.07 |
| *S. pubescens* | 15 | 7 | 14.00 | 14.00 | 14.00 | 0.60 | 0.60 | 1.00 | 1.00 | 0.04 | 0.04 |
| *S. pubescens* | 15 | 8 | 14.00 | 14.00 | 14.00 | 0.20 | 0.20 | 1.00 | 1.00 | 0.01 | 0.01 |
| *S. pubescens* | 15 | 9 | 14.00 | 14.00 | 14.00 | 1.00 | 1.00 | 1.00 | 1.00 | 0.07 | 0.07 |

| | | | | | | | | | | | |
|---|---|---|---|---|---|---|---|---|---|---|---|
| *S. pubescens* | 15 | 10 | 14.00 | 14.00 | 14.00 | 0.60 | 0.60 | 1.00 | 1.00 | 0.04 | 0.04 |
| *S. pubescens* | 15 | 11 | 14.00 | 14.00 | 14.00 | 1.00 | 1.00 | 1.00 | 1.00 | 0.07 | 0.07 |
| *S. pubescens* | 15 | 12 | 16.00 | 14.00 | 16.00 | 1.60 | 1.60 | 1.14 | 1.00 | 0.10 | 0.11 |
| *S. pubescens* | 15 | 13 | 14.00 | 14.00 | 14.00 | 1.00 | 1.00 | 1.00 | 1.00 | 0.07 | 0.07 |
| *S. pubescens* | 15 | 14 | 14.00 | 14.00 | 14.00 | 1.00 | 1.00 | 1.00 | 1.00 | 0.07 | 0.07 |
| *S. pubescens* | 15 | 15 | 12.00 | 12.00 | 12.00 | 0.60 | 0.60 | 1.00 | 1.00 | 0.05 | 0.05 |
| *S. pubescens* | 15 | 16 | 14.00 | 14.00 | 12.00 | 0.60 | 0.60 | 1.00 | 0.86 | 0.04 | 0.04 |
| *S. pubescens* | 15 | 17 | 14.00 | 14.00 | 12.00 | 0.60 | 0.60 | 1.00 | 0.86 | 0.04 | 0.04 |
| *S. pubescens* | 15 | 18 | 14.00 | 14.00 | 14.00 | 1.00 | 1.00 | 1.00 | 1.00 | 0.07 | 0.07 |
| *S. pubescens* | 15 | 19 | 14.00 | 14.00 | 12.00 | 1.00 | 1.00 | 1.00 | 0.86 | 0.07 | 0.07 |
| *S. pubescens* | 15 | 20 | 14.00 | 14.00 | 14.00 | 0.60 | 0.60 | 1.00 | 1.00 | 0.04 | 0.04 |
| *S. pubescens* | 15 | 21 | 14.00 | 14.00 | 14.00 | 0.60 | 0.60 | 1.00 | 1.00 | 0.04 | 0.04 |
| *S. pubescens* | 15 | 22 | 14.00 | 14.00 | 12.00 | 1.00 | 1.00 | 1.00 | 0.86 | 0.07 | 0.07 |
| *S. pubescens* | 15 | 23 | 16.00 | 14.00 | 16.00 | 1.00 | 1.00 | 1.14 | 1.00 | 0.06 | 0.07 |
| *S. pubescens* | 15 | 24 | 16.00 | 16.00 | 16.00 | 1.00 | 1.00 | 1.00 | 1.00 | 0.06 | 0.06 |
| *S. pubescens* | 15 | 25 | 16.00 | 12.00 | 16.00 | 0.60 | 0.60 | 1.33 | 1.00 | 0.04 | 0.05 |
| *S. pubescens* | 15 | 26 | 14.00 | 14.00 | 14.00 | 1.00 | 1.00 | 1.00 | 1.00 | 0.07 | 0.07 |
| *S. pubescens* | 15 | 27 | 16.00 | 14.00 | 16.00 | 0.60 | 0.60 | 1.14 | 1.00 | 0.04 | 0.04 |
| *S. pubescens* | 15 | 28 | 14.00 | 12.00 | 14.00 | 1.00 | 1.00 | 1.17 | 1.00 | 0.07 | 0.08 |
| *S. pubescens* | 15 | 29 | 16.00 | 16.00 | 16.00 | 1.00 | 1.00 | 1.00 | 1.00 | 0.06 | 0.06 |
| *S. pubescens* | 15 | 30 | 16.00 | 16.00 | 14.00 | 1.00 | 1.00 | 1.00 | 0.88 | 0.06 | 0.06 |
| *S. salicifolia* | 16 | 1 | 20.00 | 20.00 | 16.00 | 0.60 | 0.60 | 1.00 | 0.80 | 0.03 | 0.03 |
| *S. salicifolia* | 16 | 2 | 18.00 | 20.00 | 18.00 | 0.60 | 0.60 | 0.90 | 1.00 | 0.03 | 0.03 |
| *S. salicifolia* | 16 | 3 | 18.00 | 18.00 | 16.00 | 1.00 | 1.00 | 1.00 | 0.89 | 0.06 | 0.06 |
| *S. salicifolia* | 16 | 4 | 20.00 | 20.00 | 18.00 | 0.60 | 0.60 | 1.00 | 0.90 | 0.03 | 0.03 |
| *S. salicifolia* | 16 | 5 | 20.00 | 20.00 | 20.00 | 0.20 | 0.20 | 1.00 | 1.00 | 0.01 | 0.01 |
| *S. salicifolia* | 16 | 6 | 18.00 | 20.00 | 18.00 | 0.60 | 0.60 | 0.90 | 1.00 | 0.03 | 0.03 |
| *S. salicifolia* | 16 | 7 | 20.00 | 20.00 | 16.00 | 0.60 | 0.60 | 1.00 | 0.80 | 0.03 | 0.03 |
| *S. salicifolia* | 16 | 8 | 18.00 | 20.00 | 16.00 | 0.60 | 0.60 | 0.90 | 0.89 | 0.03 | 0.03 |
| *S. salicifolia* | 16 | 9 | 20.00 | 20.00 | 20.00 | 0.60 | 0.60 | 1.00 | 1.00 | 0.03 | 0.03 |
| *S. salicifolia* | 16 | 10 | 20.00 | 20.00 | 18.00 | 1.00 | 0.60 | 1.00 | 0.90 | 0.05 | 0.03 |
| *S. salicifolia* | 16 | 11 | 18.00 | 20.00 | 18.00 | 0.60 | 0.60 | 0.90 | 1.00 | 0.03 | 0.03 |
| *S. salicifolia* | 16 | 12 | 20.00 | 18.00 | 20.00 | 0.60 | 0.60 | 1.11 | 1.00 | 0.03 | 0.03 |
| *S. salicifolia* | 16 | 13 | 20.00 | 20.00 | 18.00 | 0.60 | 0.60 | 1.00 | 0.90 | 0.03 | 0.03 |
| *S. salicifolia* | 16 | 14 | 18.00 | 20.00 | 18.00 | 0.60 | 0.60 | 0.90 | 1.00 | 0.03 | 0.03 |
| *S. salicifolia* | 16 | 15 | 20.00 | 20.00 | 20.00 | 1.00 | 1.00 | 1.00 | 1.00 | 0.05 | 0.05 |
| *S. salicifolia* | 16 | 16 | 18.00 | 20.00 | 18.00 | 0.60 | 0.60 | 0.90 | 1.00 | 0.03 | 0.03 |
| *S. salicifolia* | 16 | 17 | 18.00 | 16.00 | 18.00 | 0.60 | 0.60 | 1.13 | 1.00 | 0.03 | 0.04 |
| *S. salicifolia* | 16 | 18 | 20.00 | 18.00 | 20.00 | 1.00 | 1.00 | 1.11 | 1.00 | 0.05 | 0.06 |
| *S. salicifolia* | 16 | 19 | 20.00 | 18.00 | 18.00 | 0.60 | 0.60 | 1.11 | 0.90 | 0.03 | 0.03 |
| *S. salicifolia* | 16 | 20 | 18.00 | 18.00 | 18.00 | 0.60 | 0.60 | 1.00 | 1.00 | 0.03 | 0.03 |
| *S. salicifolia* | 16 | 21 | 20.00 | 20.00 | 20.00 | 0.60 | 0.60 | 1.00 | 1.00 | 0.03 | 0.03 |
| *S. salicifolia* | 16 | 22 | 20.00 | 20.00 | 20.00 | 0.20 | 0.20 | 1.00 | 1.00 | 0.01 | 0.01 |
| *S. salicifolia* | 16 | 23 | 18.00 | 20.00 | 18.00 | 0.60 | 0.60 | 0.90 | 1.00 | 0.03 | 0.03 |
| *S. salicifolia* | 16 | 24 | 20.00 | 20.00 | 18.00 | 0.60 | 0.60 | 1.00 | 0.90 | 0.03 | 0.03 |
| *S. salicifolia* | 16 | 25 | 20.00 | 18.00 | 20.00 | 0.60 | 0.60 | 1.11 | 1.00 | 0.03 | 0.03 |
| *S. salicifolia* | 16 | 26 | 18.00 | 20.00 | 18.00 | 0.20 | 0.20 | 0.90 | 1.00 | 0.01 | 0.01 |
| *S. salicifolia* | 16 | 27 | 18.00 | 18.00 | 18.00 | 0.60 | 0.60 | 1.00 | 1.00 | 0.03 | 0.03 |
| *S. salicifolia* | 16 | 28 | 18.00 | 20.00 | 18.00 | 1.00 | 1.00 | 0.90 | 1.00 | 0.06 | 0.05 |
| *S. salicifolia* | 16 | 29 | 18.00 | 18.00 | 18.00 | 1.60 | 1.60 | 1.00 | 1.00 | 0.09 | 0.09 |
| *S. salicifolia* | 16 | 30 | 18.00 | 20.00 | 18.00 | 1.00 | 1.00 | 0.90 | 1.00 | 0.06 | 0.05 |
| *S. splendens* | 17 | 1 | 16.00 | 14.00 | 16.00 | 1.00 | 1.00 | 1.14 | 1.00 | 0.06 | 0.07 |
| *S. splendens* | 17 | 2 | 18.00 | 16.00 | 14.00 | 0.60 | 0.60 | 1.13 | 0.78 | 0.03 | 0.04 |
| *S. splendens* | 17 | 3 | 16.00 | 14.00 | 16.00 | 0.60 | 0.60 | 1.14 | 1.00 | 0.04 | 0.04 |

| | | | | | | | | | | | |
|---|---|---|---|---|---|---|---|---|---|---|---|
| *S. splendens* | 17 | 4 | 16.00 | 16.00 | 14.00 | 0.60 | 0.60 | 1.00 | 0.88 | 0.04 | 0.04 |
| *S. splendens* | 17 | 5 | 16.00 | 16.00 | 14.00 | 0.60 | 0.60 | 1.00 | 0.88 | 0.04 | 0.04 |
| *S. splendens* | 17 | 6 | 16.00 | 16.00 | 14.00 | 1.00 | 1.00 | 1.00 | 0.88 | 0.06 | 0.06 |
| *S. splendens* | 17 | 7 | 16.00 | 14.00 | 16.00 | 1.00 | 1.00 | 1.14 | 1.00 | 0.06 | 0.07 |
| *S. splendens* | 17 | 8 | 16.00 | 16.00 | 14.00 | 1.00 | 1.00 | 1.00 | 0.88 | 0.06 | 0.06 |
| *S. splendens* | 17 | 9 | 16.00 | 14.00 | 16.00 | 1.00 | 1.00 | 1.14 | 1.00 | 0.06 | 0.07 |
| *S. splendens* | 17 | 10 | 16.00 | 16.00 | 14.00 | 0.60 | 0.60 | 1.00 | 0.88 | 0.04 | 0.04 |
| *S. splendens* | 17 | 11 | 18.00 | 16.00 | 16.00 | 1.00 | 1.00 | 1.13 | 0.89 | 0.06 | 0.06 |
| *S. splendens* | 17 | 12 | 16.00 | 14.00 | 16.00 | 1.00 | 1.00 | 1.14 | 1.00 | 0.06 | 0.07 |
| *S. splendens* | 17 | 13 | 18.00 | 16.00 | 16.00 | 1.60 | 1.60 | 1.13 | 0.89 | 0.09 | 0.10 |
| *S. splendens* | 17 | 14 | 18.00 | 16.00 | 14.00 | 1.00 | 1.00 | 1.13 | 0.78 | 0.06 | 0.06 |
| *S. splendens* | 17 | 15 | 16.00 | 14.00 | 16.00 | 1.60 | 1.60 | 1.14 | 1.00 | 0.10 | 0.11 |
| *S. splendens* | 17 | 16 | 14.00 | 16.00 | 14.00 | 1.00 | 1.00 | 0.88 | 1.00 | 0.07 | 0.06 |
| *S. splendens* | 17 | 17 | 16.00 | 14.00 | 14.00 | 1.00 | 1.00 | 1.14 | 0.88 | 0.06 | 0.07 |
| *S. splendens* | 17 | 18 | 18.00 | 16.00 | 16.00 | 1.00 | 1.00 | 1.13 | 0.89 | 0.06 | 0.06 |
| *S. splendens* | 17 | 19 | 20.00 | 18.00 | 16.00 | 1.00 | 1.00 | 1.11 | 0.80 | 0.05 | 0.06 |
| *S. splendens* | 17 | 20 | 18.00 | 16.00 | 16.00 | 1.00 | 1.00 | 1.13 | 0.89 | 0.06 | 0.06 |
| *S. splendens* | 17 | 21 | 16.00 | 16.00 | 16.00 | 1.60 | 1.60 | 1.00 | 1.00 | 0.10 | 0.10 |
| *S. splendens* | 17 | 22 | 16.00 | 14.00 | 16.00 | 1.00 | 1.00 | 1.14 | 1.00 | 0.06 | 0.07 |
| *S. splendens* | 17 | 23 | 16.00 | 16.00 | 14.00 | 1.00 | 1.00 | 1.00 | 0.88 | 0.06 | 0.06 |
| *S. splendens* | 17 | 24 | 16.00 | 14.00 | 16.00 | 1.60 | 1.60 | 1.14 | 1.00 | 0.10 | 0.11 |
| *S. splendens* | 17 | 25 | 16.00 | 14.00 | 16.00 | 1.00 | 1.00 | 1.14 | 1.00 | 0.06 | 0.07 |
| *S. splendens* | 17 | 26 | 16.00 | 16.00 | 16.00 | 1.00 | 1.00 | 1.00 | 1.00 | 0.06 | 0.06 |
| *S. splendens* | 17 | 27 | 16.00 | 14.00 | 14.00 | 1.00 | 1.00 | 1.14 | 0.88 | 0.06 | 0.07 |
| *S. splendens* | 17 | 28 | 14.00 | 14.00 | 14.00 | 1.60 | 1.60 | 1.00 | 1.00 | 0.11 | 0.11 |
| *S. splendens* | 17 | 29 | 18.00 | 16.00 | 18.00 | 2.00 | 2.00 | 1.13 | 1.00 | 0.11 | 0.13 |
| *S. splendens* | 17 | 30 | 16.00 | 14.00 | 14.00 | 0.60 | 0.60 | 1.14 | 0.88 | 0.04 | 0.04 |
| *S. thunbergii* | 18 | 1 | 16.00 | 18.00 | 14.00 | 1.60 | 1.60 | 0.89 | 0.88 | 0.10 | 0.09 |
| *S. thunbergii* | 18 | 2 | 18.00 | 16.00 | 14.00 | 1.00 | 1.00 | 1.13 | 0.78 | 0.06 | 0.06 |
| *S. thunbergii* | 18 | 3 | 14.00 | 14.00 | 14.00 | 1.60 | 1.60 | 1.00 | 1.00 | 0.11 | 0.11 |
| *S. thunbergii* | 18 | 4 | 16.00 | 16.00 | 14.00 | 1.60 | 1.60 | 1.00 | 0.88 | 0.10 | 0.10 |
| *S. thunbergii* | 18 | 5 | 16.00 | 14.00 | 14.00 | 1.00 | 1.00 | 1.14 | 0.88 | 0.06 | 0.07 |
| *S. thunbergii* | 18 | 6 | 16.00 | 16.00 | 16.00 | 1.00 | 1.00 | 1.00 | 1.00 | 0.06 | 0.06 |
| *S. thunbergii* | 18 | 7 | 16.00 | 16.00 | 14.00 | 0.60 | 0.60 | 1.00 | 0.88 | 0.04 | 0.04 |
| *S. thunbergii* | 18 | 8 | 14.00 | 14.00 | 12.00 | 0.60 | 0.60 | 1.00 | 0.86 | 0.04 | 0.04 |
| *S. thunbergii* | 18 | 9 | 16.00 | 14.00 | 14.00 | 1.00 | 1.00 | 1.14 | 0.88 | 0.06 | 0.07 |
| *S. thunbergii* | 18 | 10 | 16.00 | 16.00 | 16.00 | 1.60 | 1.60 | 1.00 | 1.00 | 0.10 | 0.10 |
| *S. thunbergii* | 18 | 11 | 14.00 | 14.00 | 12.00 | 0.60 | 0.60 | 1.00 | 0.86 | 0.04 | 0.04 |
| *S. thunbergii* | 18 | 12 | 14.00 | 12.00 | 12.00 | 0.60 | 0.60 | 1.17 | 0.86 | 0.04 | 0.05 |
| *S. thunbergii* | 18 | 13 | 16.00 | 14.00 | 16.00 | 1.00 | 1.00 | 1.14 | 1.00 | 0.06 | 0.07 |
| *S. thunbergii* | 18 | 14 | 16.00 | 16.00 | 14.00 | 1.60 | 1.60 | 1.00 | 0.88 | 0.10 | 0.10 |
| *S. thunbergii* | 18 | 15 | 16.00 | 16.00 | 14.00 | 1.00 | 1.00 | 1.00 | 0.88 | 0.06 | 0.06 |
| *S. thunbergii* | 18 | 16 | 14.00 | 14.00 | 14.00 | 1.00 | 1.00 | 1.00 | 1.00 | 0.07 | 0.07 |
| *S. thunbergii* | 18 | 17 | 16.00 | 16.00 | 12.00 | 1.00 | 1.00 | 1.00 | 0.75 | 0.06 | 0.06 |
| *S. thunbergii* | 18 | 18 | 14.00 | 14.00 | 12.00 | 1.60 | 1.60 | 1.00 | 0.86 | 0.11 | 0.11 |
| *S. thunbergii* | 18 | 19 | 16.00 | 14.00 | 12.00 | 1.00 | 1.00 | 1.14 | 0.75 | 0.06 | 0.07 |
| *S. thunbergii* | 18 | 20 | 16.00 | 16.00 | 16.00 | 1.60 | 1.60 | 1.00 | 1.00 | 0.10 | 0.10 |
| *S. thunbergii* | 18 | 21 | 16.00 | 16.00 | 14.00 | 1.60 | 1.60 | 1.00 | 0.88 | 0.10 | 0.10 |
| *S. thunbergii* | 18 | 22 | 16.00 | 14.00 | 12.00 | 1.00 | 1.00 | 1.14 | 0.75 | 0.06 | 0.07 |
| *S. thunbergii* | 18 | 23 | 14.00 | 14.00 | 12.00 | 0.60 | 0.60 | 1.00 | 0.86 | 0.04 | 0.04 |
| *S. thunbergii* | 18 | 24 | 14.00 | 14.00 | 12.00 | 0.60 | 0.60 | 1.00 | 0.86 | 0.04 | 0.04 |
| *S. thunbergii* | 18 | 25 | 14.00 | 14.00 | 12.00 | 0.60 | 0.60 | 1.00 | 0.86 | 0.04 | 0.04 |
| *S. thunbergii* | 18 | 26 | 14.00 | 16.00 | 12.00 | 1.00 | 1.00 | 0.88 | 0.86 | 0.07 | 0.06 |
| *S. thunbergii* | 18 | 27 | 16.00 | 16.00 | 16.00 | 1.00 | 1.00 | 1.00 | 1.00 | 0.06 | 0.06 |

| | | | | | | | | | | | |
|---|---|---|---|---|---|---|---|---|---|---|---|
| *S. thunbergii* | 18 | 28 | 16.00 | 16.00 | 14.00 | 1.00 | 1.00 | 1.00 | 0.88 | 0.06 | 0.06 |
| *S. thunbergii* | 18 | 29 | 16.00 | 14.00 | 12.00 | 1.00 | 1.00 | 1.14 | 0.75 | 0.06 | 0.07 |
| *S. thunbergii* | 18 | 30 | 16.00 | 16.00 | 16.00 | 1.60 | 1.60 | 1.00 | 1.00 | 0.10 | 0.10 |
| *S. tomentosa* | 19 | 1 | 16.00 | 16.00 | 14.00 | 2.00 | 1.00 | 1.00 | 0.88 | 0.125 | 0.0625 |
| *S. tomentosa* | 19 | 2 | 18.00 | 14.00 | 12.00 | 2.00 | 2.00 | 1.29 | 0.67 | 0.111111111 | 0.142857143 |
| *S. tomentosa* | 19 | 3 | 14.00 | 16.00 | 10.00 | 1.00 | 1.00 | 0.88 | 0.71 | 0.071428571 | 0.0625 |
| *S. tomentosa* | 19 | 4 | 16.00 | 16.00 | 14.00 | 2.00 | 2.00 | 1.00 | 0.88 | 0.125 | 0.125 |
| *S. tomentosa* | 19 | 5 | 14.00 | 16.00 | 12.00 | 2.00 | 1.00 | 0.88 | 0.86 | 0.142857143 | 0.0625 |
| *S. tomentosa* | 19 | 6 | 16.00 | 14.00 | 14.00 | 1.00 | 1.00 | 1.14 | 0.88 | 0.0625 | 0.071428571 |
| *S. tomentosa* | 19 | 7 | 14.00 | 16.00 | 12.00 | 2.00 | 1.00 | 0.88 | 0.86 | 0.142857143 | 0.0625 |
| *S. tomentosa* | 19 | 8 | 20.00 | 16.00 | 14.00 | 2.00 | 1.00 | 1.25 | 0.70 | 0.1 | 0.0625 |
| *S. tomentosa* | 19 | 9 | 18.00 | 16.00 | 14.00 | 2.00 | 1.00 | 1.13 | 0.78 | 0.111111111 | 0.0625 |
| *S. tomentosa* | 19 | 10 | 16.00 | 14.00 | 14.00 | 1.00 | 1.00 | 1.14 | 0.88 | 0.0625 | 0.071428571 |
| *S. tomentosa* | 19 | 11 | 16.00 | 16.00 | 14.00 | 1.00 | 0.60 | 1.00 | 0.88 | 0.0625 | 0.0375 |
| *S. tomentosa* | 19 | 12 | 18.00 | 16.00 | 12.00 | 1.00 | 0.60 | 1.13 | 0.67 | 0.055555556 | 0.0375 |
| *S. tomentosa* | 19 | 13 | 14.00 | 18.00 | 12.00 | 2.00 | 2.00 | 0.78 | 0.86 | 0.142857143 | 0.111111111 |
| *S. tomentosa* | 19 | 14 | 14.00 | 16.00 | 12.00 | 2.00 | 1.00 | 0.88 | 0.86 | 0.142857143 | 0.0625 |
| *S. tomentosa* | 19 | 15 | 16.00 | 16.00 | 12.00 | 2.00 | 1.00 | 1.00 | 0.75 | 0.125 | 0.0625 |
| *S. tomentosa* | 19 | 16 | 18.00 | 18.00 | 14.00 | 2.00 | 1.00 | 1.00 | 0.78 | 0.111111111 | 0.055555556 |
| *S. tomentosa* | 19 | 17 | 16.00 | 16.00 | 14.00 | 1.00 | 1.00 | 1.00 | 0.88 | 0.0625 | 0.0625 |
| *S. tomentosa* | 19 | 18 | 14.00 | 18.00 | 12.00 | 2.00 | 1.00 | 0.78 | 0.86 | 0.142857143 | 0.055555556 |
| *S. tomentosa* | 19 | 19 | 16.00 | 18.00 | 14.00 | 1.00 | 2.00 | 0.89 | 0.88 | 0.0625 | 0.111111111 |
| *S. tomentosa* | 19 | 20 | 16.00 | 18.00 | 14.00 | 2.00 | 2.00 | 0.89 | 0.88 | 0.125 | 0.111111111 |
| *S. tomentosa* | 19 | 21 | 16.00 | 16.00 | 12.00 | 2.00 | 2.00 | 1.00 | 0.75 | 0.125 | 0.125 |
| *S. tomentosa* | 19 | 22 | 18.00 | 16.00 | 16.00 | 1.00 | 2.00 | 1.13 | 0.89 | 0.055555556 | 0.125 |
| *S. tomentosa* | 19 | 23 | 18.00 | 16.00 | 12.00 | 2.00 | 1.00 | 1.13 | 0.67 | 0.111111111 | 0.0625 |
| *S. tomentosa* | 19 | 24 | 16.00 | 14.00 | 10.00 | 2.00 | 1.00 | 1.14 | 0.63 | 0.125 | 0.071428571 |
| *S. tomentosa* | 19 | 25 | 16.00 | 14.00 | 12.00 | 2.00 | 1.00 | 1.14 | 0.75 | 0.125 | 0.071428571 |
| *S. tomentosa* | 19 | 26 | 20.00 | 16.00 | 18.00 | 2.00 | 2.00 | 1.25 | 0.90 | 0.1 | 0.125 |
| *S. tomentosa* | 19 | 27 | 16.00 | 18.00 | 14.00 | 2.00 | 2.00 | 0.89 | 0.88 | 0.125 | 0.111111111 |
| *S. tomentosa* | 19 | 28 | 18.00 | 18.00 | 16.00 | 1.00 | 1.00 | 1.00 | 0.89 | 0.055555556 | 0.055555556 |
| *S. tomentosa* | 19 | 29 | 16.00 | 18.00 | 14.00 | 2.00 | 1.00 | 0.89 | 0.88 | 0.125 | 0.055555556 |
| *S. tomentosa* | 19 | 30 | 16.00 | 16.00 | 12.00 | 1.00 | 1.00 | 1.00 | 0.75 | 0.0625 | 0.0625 |
| *S. trichocarpa* | 20 | 1 | 18.00 | 16.00 | 16.00 | 0.60 | 0.60 | 1.13 | 0.89 | 0.03 | 0.04 |
| *S. trichocarpa* | 20 | 2 | 16.00 | 14.00 | 14.00 | 1.00 | 1.00 | 1.14 | 0.88 | 0.06 | 0.07 |
| *S. trichocarpa* | 20 | 3 | 16.00 | 16.00 | 16.00 | 1.00 | 1.00 | 1.00 | 1.00 | 0.06 | 0.06 |
| *S. trichocarpa* | 20 | 4 | 16.00 | 16.00 | 14.00 | 1.00 | 1.00 | 1.00 | 0.88 | 0.06 | 0.06 |
| *S. trichocarpa* | 20 | 5 | 18.00 | 16.00 | 16.00 | 1.00 | 1.00 | 1.13 | 0.89 | 0.06 | 0.06 |
| *S. trichocarpa* | 20 | 6 | 16.00 | 16.00 | 16.00 | 1.60 | 1.60 | 1.00 | 1.00 | 0.10 | 0.10 |
| *S. trichocarpa* | 20 | 7 | 16.00 | 16.00 | 16.00 | 1.00 | 1.00 | 1.00 | 1.00 | 0.06 | 0.06 |
| *S. trichocarpa* | 20 | 8 | 16.00 | 14.00 | 16.00 | 1.60 | 1.60 | 1.14 | 1.00 | 0.10 | 0.11 |
| *S. trichocarpa* | 20 | 9 | 16.00 | 14.00 | 16.00 | 1.60 | 1.60 | 1.14 | 1.00 | 0.10 | 0.11 |
| *S. trichocarpa* | 20 | 10 | 16.00 | 16.00 | 14.00 | 1.00 | 1.00 | 1.00 | 0.88 | 0.06 | 0.06 |
| *S. trichocarpa* | 20 | 11 | 16.00 | 16.00 | 14.00 | 1.00 | 1.00 | 1.00 | 0.88 | 0.06 | 0.06 |
| *S. trichocarpa* | 20 | 12 | 16.00 | 16.00 | 16.00 | 1.60 | 1.60 | 1.00 | 1.00 | 0.10 | 0.10 |
| *S. trichocarpa* | 20 | 13 | 14.00 | 16.00 | 14.00 | 1.00 | 1.00 | 0.88 | 1.00 | 0.07 | 0.06 |
| *S. trichocarpa* | 20 | 14 | 16.00 | 16.00 | 14.00 | 1.00 | 1.00 | 1.00 | 0.88 | 0.06 | 0.06 |
| *S. trichocarpa* | 20 | 15 | 14.00 | 14.00 | 12.00 | 1.00 | 1.00 | 1.00 | 0.86 | 0.07 | 0.07 |
| *S. trichocarpa* | 20 | 16 | 16.00 | 16.00 | 14.00 | 1.00 | 1.00 | 1.00 | 0.88 | 0.06 | 0.06 |
| *S. trichocarpa* | 20 | 17 | 16.00 | 16.00 | 16.00 | 1.60 | 1.60 | 1.00 | 1.00 | 0.10 | 0.10 |
| *S. trichocarpa* | 20 | 18 | 16.00 | 16.00 | 14.00 | 1.00 | 1.00 | 1.00 | 0.88 | 0.06 | 0.06 |
| *S. trichocarpa* | 20 | 19 | 16.00 | 14.00 | 14.00 | 1.00 | 1.00 | 1.14 | 0.88 | 0.06 | 0.07 |
| *S. trichocarpa* | 20 | 20 | 14.00 | 14.00 | 12.00 | 1.00 | 1.00 | 1.00 | 0.86 | 0.07 | 0.07 |
| *S. trichocarpa* | 20 | 21 | 16.00 | 14.00 | 14.00 | 0.60 | 0.60 | 1.14 | 0.88 | 0.04 | 0.04 |

| | | | | | | | | | | | |
|---|---|---|---|---|---|---|---|---|---|---|---|
| *S. trichocarpa* | 20 | 22 | 14.00 | 14.00 | 12.00 | 1.00 | 1.00 | 1.00 | 0.86 | 0.07 | 0.07 |
| *S. trichocarpa* | 20 | 23 | 14.00 | 14.00 | 14.00 | 0.60 | 0.60 | 1.00 | 1.00 | 0.04 | 0.04 |
| *S. trichocarpa* | 20 | 24 | 16.00 | 16.00 | 14.00 | 1.00 | 1.00 | 1.00 | 0.88 | 0.06 | 0.06 |
| *S. trichocarpa* | 20 | 25 | 16.00 | 16.00 | 16.00 | 1.00 | 1.00 | 1.00 | 1.00 | 0.06 | 0.06 |
| *S. trichocarpa* | 20 | 26 | 16.00 | 16.00 | 16.00 | 1.00 | 1.00 | 1.00 | 1.00 | 0.06 | 0.06 |
| *S. trichocarpa* | 20 | 27 | 16.00 | 16.00 | 14.00 | 1.60 | 1.60 | 1.00 | 0.88 | 0.10 | 0.10 |
| *S. trichocarpa* | 20 | 28 | 16.00 | 16.00 | 14.00 | 1.00 | 1.00 | 1.00 | 0.88 | 0.06 | 0.06 |
| *S. trichocarpa* | 20 | 29 | 16.00 | 16.00 | 14.00 | 1.00 | 1.00 | 1.00 | 0.88 | 0.06 | 0.06 |
| *S. trichocarpa* | 20 | 30 | 16.00 | 16.00 | 16.00 | 1.00 | 1.00 | 1.00 | 1.00 | 0.06 | 0.06 |
| *S. uratensis* | 21 | 1 | 16.00 | 14.00 | 14.00 | 0.60 | 0.60 | 1.14 | 0.88 | 0.04 | 0.04 |
| *S. uratensis* | 21 | 2 | 18.00 | 16.00 | 18.00 | 1.00 | 1.00 | 1.13 | 1.00 | 0.06 | 0.06 |
| *S. uratensis* | 21 | 3 | 18.00 | 16.00 | 18.00 | 1.00 | 1.00 | 1.13 | 1.00 | 0.06 | 0.06 |
| *S. uratensis* | 21 | 4 | 18.00 | 16.00 | 16.00 | 1.60 | 1.60 | 1.13 | 0.89 | 0.09 | 0.10 |
| *S. uratensis* | 21 | 5 | 18.00 | 16.00 | 18.00 | 1.00 | 1.00 | 1.13 | 1.00 | 0.06 | 0.06 |
| *S. uratensis* | 21 | 6 | 18.00 | 16.00 | 18.00 | 1.60 | 1.60 | 1.13 | 1.00 | 0.09 | 0.10 |
| *S. uratensis* | 21 | 7 | 18.00 | 16.00 | 18.00 | 1.60 | 1.60 | 1.13 | 1.00 | 0.09 | 0.10 |
| *S. uratensis* | 21 | 8 | 16.00 | 16.00 | 14.00 | 1.60 | 1.60 | 1.00 | 0.88 | 0.10 | 0.10 |
| *S. uratensis* | 21 | 9 | 16.00 | 16.00 | 16.00 | 1.00 | 1.00 | 1.00 | 1.00 | 0.06 | 0.06 |
| *S. uratensis* | 21 | 10 | 20.00 | 14.00 | 20.00 | 1.00 | 1.00 | 1.43 | 1.00 | 0.05 | 0.07 |
| *S. uratensis* | 21 | 11 | 18.00 | 14.00 | 18.00 | 1.00 | 1.00 | 1.29 | 1.00 | 0.06 | 0.07 |
| *S. uratensis* | 21 | 12 | 18.00 | 16.00 | 18.00 | 1.60 | 1.60 | 1.13 | 1.00 | 0.09 | 0.10 |
| *S. uratensis* | 21 | 13 | 18.00 | 16.00 | 18.00 | 1.00 | 1.00 | 1.13 | 1.00 | 0.06 | 0.06 |
| *S. uratensis* | 21 | 14 | 16.00 | 14.00 | 16.00 | 1.00 | 1.00 | 1.14 | 1.00 | 0.06 | 0.07 |
| *S. uratensis* | 21 | 15 | 18.00 | 16.00 | 18.00 | 1.00 | 1.00 | 1.13 | 1.00 | 0.06 | 0.06 |
| *S. uratensis* | 21 | 16 | 16.00 | 14.00 | 16.00 | 1.60 | 1.60 | 1.14 | 1.00 | 0.10 | 0.11 |
| *S. uratensis* | 21 | 17 | 16.00 | 16.00 | 16.00 | 1.60 | 1.60 | 1.00 | 1.00 | 0.10 | 0.10 |
| *S. uratensis* | 21 | 18 | 18.00 | 16.00 | 18.00 | 1.60 | 1.60 | 1.13 | 1.00 | 0.09 | 0.10 |
| *S. uratensis* | 21 | 19 | 18.00 | 16.00 | 16.00 | 1.00 | 1.00 | 1.13 | 0.89 | 0.06 | 0.06 |
| *S. uratensis* | 21 | 20 | 18.00 | 16.00 | 18.00 | 1.60 | 1.60 | 1.13 | 1.00 | 0.09 | 0.10 |
| *S. uratensis* | 21 | 21 | 18.00 | 14.00 | 16.00 | 1.00 | 1.00 | 1.29 | 0.89 | 0.06 | 0.07 |
| *S. uratensis* | 21 | 22 | 18.00 | 16.00 | 18.00 | 1.60 | 1.60 | 1.13 | 1.00 | 0.09 | 0.10 |
| *S. uratensis* | 21 | 23 | 18.00 | 16.00 | 16.00 | 0.60 | 0.60 | 1.13 | 0.89 | 0.03 | 0.04 |
| *S. uratensis* | 21 | 24 | 18.00 | 16.00 | 18.00 | 1.00 | 1.00 | 1.13 | 1.00 | 0.06 | 0.06 |
| *S. uratensis* | 21 | 25 | 16.00 | 14.00 | 16.00 | 0.60 | 0.60 | 1.14 | 1.00 | 0.04 | 0.04 |
| *S. uratensis* | 21 | 26 | 18.00 | 16.00 | 18.00 | 1.00 | 1.00 | 1.13 | 1.00 | 0.06 | 0.06 |
| *S. uratensis* | 21 | 27 | 16.00 | 14.00 | 16.00 | 1.60 | 1.60 | 1.14 | 1.00 | 0.10 | 0.11 |
| *S. uratensis* | 21 | 28 | 18.00 | 16.00 | 16.00 | 1.60 | 1.60 | 1.13 | 0.89 | 0.09 | 0.10 |
| *S. uratensis* | 21 | 29 | 18.00 | 16.00 | 18.00 | 1.60 | 1.60 | 1.13 | 1.00 | 0.09 | 0.10 |
| *S. uratensis* | 21 | 30 | 18.00 | 14.00 | 16.00 | 1.00 | 1.00 | 1.29 | 0.89 | 0.06 | 0.07 |
| *S. veitchii* | 22 | 1 | 16.00 | 14.00 | 14.00 | 1.00 | 1.00 | 1.14 | 0.88 | 0.06 | 0.07 |
| *S. veitchii* | 22 | 2 | 16.00 | 16.00 | 14.00 | 1.00 | 1.00 | 1.00 | 0.88 | 0.06 | 0.06 |
| *S. veitchii* | 22 | 3 | 16.00 | 16.00 | 16.00 | 0.60 | 0.60 | 1.00 | 1.00 | 0.04 | 0.04 |
| *S. veitchii* | 22 | 4 | 16.00 | 14.00 | 14.00 | 1.60 | 1.60 | 1.14 | 0.88 | 0.10 | 0.11 |
| *S. veitchii* | 22 | 5 | 16.00 | 16.00 | 14.00 | 1.00 | 1.00 | 1.00 | 0.88 | 0.06 | 0.06 |
| *S. veitchii* | 22 | 6 | 16.00 | 16.00 | 14.00 | 0.60 | 0.60 | 1.00 | 0.88 | 0.04 | 0.04 |
| *S. veitchii* | 22 | 7 | 20.00 | 20.00 | 20.00 | 1.60 | 1.60 | 1.00 | 1.00 | 0.08 | 0.08 |
| *S. veitchii* | 22 | 8 | 18.00 | 16.00 | 14.00 | 0.60 | 0.60 | 1.13 | 0.78 | 0.03 | 0.04 |
| *S. veitchii* | 22 | 9 | 18.00 | 16.00 | 14.00 | 1.00 | 1.00 | 1.13 | 0.78 | 0.06 | 0.06 |
| *S. veitchii* | 22 | 10 | 18.00 | 16.00 | 18.00 | 0.60 | 0.60 | 1.13 | 1.00 | 0.03 | 0.04 |
| *S. veitchii* | 22 | 11 | 14.00 | 16.00 | 12.00 | 1.60 | 1.60 | 0.88 | 0.86 | 0.11 | 0.10 |
| *S. veitchii* | 22 | 12 | 16.00 | 14.00 | 12.00 | 1.00 | 1.00 | 1.14 | 0.75 | 0.06 | 0.07 |
| *S. veitchii* | 22 | 13 | 18.00 | 16.00 | 16.00 | 1.00 | 1.00 | 1.13 | 0.89 | 0.06 | 0.06 |
| *S. veitchii* | 22 | 14 | 18.00 | 18.00 | 18.00 | 1.60 | 1.60 | 1.00 | 1.00 | 0.09 | 0.09 |
| *S. veitchii* | 22 | 15 | 16.00 | 18.00 | 12.00 | 1.00 | 1.00 | 0.89 | 0.75 | 0.06 | 0.06 |

| | | | | | | | | | | | |
|---|---|---|---|---|---|---|---|---|---|---|---|
| *S. veitchii* | 22 | 16 | 16.00 | 16.00 | 14.00 | 1.00 | 1.00 | 1.00 | 0.88 | 0.06 | 0.06 |
| *S. veitchii* | 22 | 17 | 16.00 | 16.00 | 16.00 | 0.60 | 0.60 | 1.00 | 1.00 | 0.04 | 0.04 |
| *S. veitchii* | 22 | 18 | 16.00 | 16.00 | 14.00 | 0.60 | 0.60 | 1.00 | 0.88 | 0.04 | 0.04 |
| *S. veitchii* | 22 | 19 | 18.00 | 18.00 | 18.00 | 1.60 | 1.60 | 1.00 | 1.00 | 0.09 | 0.09 |
| *S. veitchii* | 22 | 20 | 16.00 | 14.00 | 14.00 | 1.00 | 1.00 | 1.14 | 0.88 | 0.06 | 0.07 |
| *S. veitchii* | 22 | 21 | 14.00 | 16.00 | 14.00 | 0.60 | 0.60 | 0.88 | 1.00 | 0.04 | 0.04 |
| *S. veitchii* | 22 | 22 | 16.00 | 14.00 | 12.00 | 1.00 | 1.00 | 1.14 | 0.75 | 0.06 | 0.07 |
| *S. veitchii* | 22 | 23 | 18.00 | 16.00 | 14.00 | 1.00 | 1.00 | 1.13 | 0.78 | 0.06 | 0.06 |
| *S. veitchii* | 22 | 24 | 18.00 | 16.00 | 18.00 | 1.60 | 1.60 | 1.13 | 1.00 | 0.09 | 0.10 |
| *S. veitchii* | 22 | 25 | 16.00 | 16.00 | 14.00 | 1.00 | 1.00 | 1.00 | 0.88 | 0.06 | 0.06 |
| *S. veitchii* | 22 | 26 | 16.00 | 16.00 | 16.00 | 1.00 | 1.00 | 1.00 | 1.00 | 0.06 | 0.06 |
| *S. veitchii* | 22 | 27 | 16.00 | 14.00 | 16.00 | 0.60 | 0.60 | 1.14 | 1.00 | 0.04 | 0.04 |
| *S. veitchii* | 22 | 28 | 16.00 | 16.00 | 12.00 | 1.60 | 1.60 | 1.00 | 0.75 | 0.10 | 0.10 |
| *S. veitchii* | 22 | 29 | 16.00 | 16.00 | 14.00 | 1.00 | 1.00 | 1.00 | 0.88 | 0.06 | 0.06 |
| *S. veitchii* | 22 | 30 | 16.00 | 14.00 | 12.00 | 0.60 | 0.60 | 1.14 | 0.75 | 0.04 | 0.04 |
| *S. wilsonii* | 23 | 1 | 18.00 | 16.00 | 16.00 | 0.60 | 0.60 | 1.13 | 0.89 | 0.03 | 0.04 |
| *S. wilsonii* | 23 | 2 | 16.00 | 16.00 | 14.00 | 1.00 | 1.00 | 1.00 | 0.88 | 0.06 | 0.06 |
| *S. wilsonii* | 23 | 3 | 16.00 | 14.00 | 14.00 | 0.60 | 0.60 | 1.14 | 0.88 | 0.04 | 0.04 |
| *S. wilsonii* | 23 | 4 | 16.00 | 14.00 | 16.00 | 1.00 | 1.00 | 1.14 | 1.00 | 0.06 | 0.07 |
| *S. wilsonii* | 23 | 5 | 16.00 | 16.00 | 12.00 | 0.60 | 0.60 | 1.00 | 0.75 | 0.04 | 0.04 |
| *S. wilsonii* | 23 | 6 | 16.00 | 16.00 | 14.00 | 0.60 | 0.60 | 1.00 | 0.88 | 0.04 | 0.04 |
| *S. wilsonii* | 23 | 7 | 16.00 | 16.00 | 12.00 | 0.60 | 0.60 | 1.00 | 0.75 | 0.04 | 0.04 |
| *S. wilsonii* | 23 | 8 | 16.00 | 14.00 | 14.00 | 0.60 | 0.60 | 1.14 | 0.88 | 0.04 | 0.04 |
| *S. wilsonii* | 23 | 9 | 14.00 | 14.00 | 14.00 | 1.60 | 1.60 | 1.00 | 1.00 | 0.11 | 0.11 |
| *S. wilsonii* | 23 | 10 | 16.00 | 14.00 | 16.00 | 1.00 | 1.00 | 1.14 | 1.00 | 0.06 | 0.07 |
| *S. wilsonii* | 23 | 11 | 16.00 | 14.00 | 12.00 | 1.00 | 1.00 | 1.14 | 0.75 | 0.06 | 0.07 |
| *S. wilsonii* | 23 | 12 | 16.00 | 14.00 | 14.00 | 1.60 | 1.60 | 1.14 | 0.88 | 0.10 | 0.11 |
| *S. wilsonii* | 23 | 13 | 16.00 | 16.00 | 12.00 | 1.00 | 1.00 | 1.00 | 0.75 | 0.06 | 0.06 |
| *S. wilsonii* | 23 | 14 | 14.00 | 14.00 | 14.00 | 0.60 | 0.60 | 1.00 | 1.00 | 0.04 | 0.04 |
| *S. wilsonii* | 23 | 15 | 16.00 | 14.00 | 16.00 | 0.60 | 0.60 | 1.14 | 1.00 | 0.04 | 0.04 |
| *S. wilsonii* | 23 | 16 | 16.00 | 14.00 | 14.00 | 0.60 | 0.60 | 1.14 | 0.88 | 0.04 | 0.04 |
| *S. wilsonii* | 23 | 17 | 16.00 | 16.00 | 14.00 | 1.00 | 1.00 | 1.00 | 0.88 | 0.06 | 0.06 |
| *S. wilsonii* | 23 | 18 | 16.00 | 16.00 | 16.00 | 0.60 | 0.60 | 1.00 | 1.00 | 0.04 | 0.04 |
| *S. wilsonii* | 23 | 19 | 16.00 | 16.00 | 14.00 | 0.60 | 0.60 | 1.00 | 0.88 | 0.04 | 0.04 |
| *S. wilsonii* | 23 | 20 | 14.00 | 16.00 | 14.00 | 1.00 | 1.00 | 0.88 | 1.00 | 0.07 | 0.06 |
| *S. wilsonii* | 23 | 21 | 14.00 | 14.00 | 12.00 | 0.60 | 0.60 | 1.00 | 0.86 | 0.04 | 0.04 |
| *S. wilsonii* | 23 | 22 | 16.00 | 16.00 | 14.00 | 0.60 | 0.60 | 1.00 | 0.88 | 0.04 | 0.04 |
| *S. wilsonii* | 23 | 23 | 16.00 | 16.00 | 14.00 | 1.00 | 0.60 | 1.00 | 0.88 | 0.06 | 0.04 |
| *S. wilsonii* | 23 | 24 | 14.00 | 14.00 | 14.00 | 1.60 | 1.60 | 1.00 | 1.00 | 0.11 | 0.11 |
| *S. wilsonii* | 23 | 25 | 16.00 | 16.00 | 14.00 | 1.00 | 1.00 | 1.00 | 0.88 | 0.06 | 0.06 |
| *S. wilsonii* | 23 | 26 | 16.00 | 14.00 | 16.00 | 1.00 | 1.00 | 1.14 | 1.00 | 0.06 | 0.07 |
| *S. wilsonii* | 23 | 27 | 18.00 | 16.00 | 16.00 | 1.60 | 1.60 | 1.13 | 0.89 | 0.09 | 0.10 |
| *S. wilsonii* | 23 | 28 | 16.00 | 14.00 | 14.00 | 1.00 | 1.00 | 1.14 | 0.88 | 0.06 | 0.07 |
| *S. wilsonii* | 23 | 29 | 14.00 | 12.00 | 12.00 | 1.00 | 1.00 | 1.17 | 0.86 | 0.07 | 0.08 |
| *S. wilsonii* | 23 | 30 | 16.00 | 16.00 | 16.00 | 1.00 | 1.00 | 1.00 | 1.00 | 0.06 | 0.06 |
| *S. xbillardii* | 24 | 1 | 16.00 | 14.00 | 14.00 | 1.00 | 1.00 | 1.14 | 0.88 | 0.06 | 0.07 |
| *S. xbillardii* | 24 | 2 | 18.00 | 14.00 | 16.00 | 1.60 | 1.60 | 1.29 | 0.89 | 0.09 | 0.11 |
| *S. xbillardii* | 24 | 3 | 18.00 | 14.00 | 14.00 | 1.60 | 1.60 | 1.29 | 0.78 | 0.09 | 0.11 |
| *S. xbillardii* | 24 | 4 | 18.00 | 16.00 | 18.00 | 0.60 | 0.60 | 1.13 | 1.00 | 0.03 | 0.04 |
| *S. xbillardii* | 24 | 5 | 16.00 | 14.00 | 16.00 | 0.60 | 0.60 | 1.14 | 1.00 | 0.04 | 0.04 |
| *S. xbillardii* | 24 | 6 | 18.00 | 18.00 | 18.00 | 0.60 | 0.60 | 1.00 | 1.00 | 0.03 | 0.03 |
| *S. xbillardii* | 24 | 7 | 20.00 | 18.00 | 20.00 | 0.60 | 0.60 | 1.11 | 1.00 | 0.03 | 0.03 |
| *S. xbillardii* | 24 | 8 | 18.00 | 18.00 | 18.00 | 1.00 | 1.00 | 1.00 | 1.00 | 0.06 | 0.06 |
| *S. xbillardii* | 24 | 9 | 20.00 | 18.00 | 20.00 | 0.60 | 0.60 | 1.11 | 1.00 | 0.03 | 0.03 |

| | | | | | | | | | | | |
|---|---|---|---|---|---|---|---|---|---|---|---|
| *S. xbillardii* | 24 | 10 | 18.00 | 16.00 | 18.00 | 1.00 | 1.00 | 1.13 | 1.00 | 0.06 | 0.06 |
| *S. xbillardii* | 24 | 11 | 20.00 | 20.00 | 20.00 | 0.60 | 0.60 | 1.00 | 1.00 | 0.03 | 0.03 |
| *S. xbillardii* | 24 | 12 | 18.00 | 16.00 | 16.00 | 0.60 | 0.60 | 1.13 | 0.89 | 0.03 | 0.04 |
| *S. xbillardii* | 24 | 13 | 20.00 | 18.00 | 20.00 | 1.00 | 1.00 | 1.11 | 1.00 | 0.05 | 0.06 |
| *S. xbillardii* | 24 | 14 | 18.00 | 18.00 | 16.00 | 0.60 | 0.60 | 1.00 | 0.89 | 0.03 | 0.03 |
| *S. xbillardii* | 24 | 15 | 18.00 | 16.00 | 18.00 | 0.60 | 0.60 | 1.13 | 1.00 | 0.03 | 0.04 |
| *S. xbillardii* | 24 | 16 | 18.00 | 18.00 | 16.00 | 1.00 | 1.00 | 1.00 | 0.89 | 0.06 | 0.06 |
| *S. xbillardii* | 24 | 17 | 20.00 | 16.00 | 20.00 | 0.60 | 0.60 | 1.25 | 1.00 | 0.03 | 0.04 |
| *S. xbillardii* | 24 | 18 | 20.00 | 18.00 | 18.00 | 1.00 | 1.00 | 1.11 | 0.90 | 0.05 | 0.06 |
| *S. xbillardii* | 24 | 19 | 16.00 | 16.00 | 16.00 | 1.00 | 1.00 | 1.00 | 1.00 | 0.06 | 0.06 |
| *S. xbillardii* | 24 | 20 | 20.00 | 16.00 | 18.00 | 0.60 | 0.60 | 1.25 | 0.90 | 0.03 | 0.04 |
| *S. xbillardii* | 24 | 21 | 14.00 | 14.00 | 14.00 | 1.00 | 1.00 | 1.00 | 1.00 | 0.07 | 0.07 |
| *S. xbillardii* | 24 | 22 | 18.00 | 16.00 | 16.00 | 1.00 | 1.00 | 1.13 | 0.89 | 0.06 | 0.06 |
| *S. xbillardii* | 24 | 23 | 18.00 | 16.00 | 14.00 | 1.00 | 1.00 | 1.13 | 0.78 | 0.06 | 0.06 |
| *S. xbillardii* | 24 | 24 | 18.00 | 14.00 | 18.00 | 0.60 | 0.60 | 1.29 | 1.00 | 0.03 | 0.04 |
| *S. xbillardii* | 24 | 25 | 18.00 | 14.00 | 16.00 | 1.00 | 1.00 | 1.29 | 0.89 | 0.06 | 0.07 |
| *S. xbillardii* | 24 | 26 | 18.00 | 16.00 | 18.00 | 0.60 | 0.60 | 1.13 | 1.00 | 0.03 | 0.04 |
| *S. xbillardii* | 24 | 27 | 18.00 | 16.00 | 16.00 | 0.60 | 0.60 | 1.13 | 0.89 | 0.03 | 0.04 |
| *S. xbillardii* | 24 | 28 | 18.00 | 16.00 | 18.00 | 1.00 | 1.00 | 1.13 | 1.00 | 0.06 | 0.06 |
| *S. xbillardii* | 24 | 29 | 18.00 | 16.00 | 16.00 | 1.00 | 1.00 | 1.13 | 0.89 | 0.06 | 0.06 |
| *S. xbillardii* | 24 | 30 | 18.00 | 18.00 | 18.00 | 0.60 | 0.60 | 1.00 | 1.00 | 0.03 | 0.03 |
| *S. xcinerea* | 25 | 1 | 18.00 | 14.00 | 18.00 | 1.00 | 1.00 | 1.29 | 1.00 | 0.06 | 0.07 |
| *S. xcinerea* | 25 | 2 | 14.00 | 14.00 | 14.00 | 1.60 | 1.60 | 1.00 | 1.00 | 0.11 | 0.11 |
| *S. xcinerea* | 25 | 3 | 14.00 | 14.00 | 12.00 | 1.00 | 1.00 | 1.00 | 0.86 | 0.07 | 0.07 |
| *S. xcinerea* | 25 | 4 | 16.00 | 14.00 | 14.00 | 1.00 | 1.00 | 1.14 | 0.88 | 0.06 | 0.07 |
| *S. xcinerea* | 25 | 5 | 18.00 | 16.00 | 16.00 | 1.60 | 1.60 | 1.13 | 0.89 | 0.09 | 0.10 |
| *S. xcinerea* | 25 | 6 | 18.00 | 16.00 | 18.00 | 1.60 | 1.60 | 1.13 | 1.00 | 0.09 | 0.10 |
| *S. xcinerea* | 25 | 7 | 14.00 | 12.00 | 14.00 | 0.60 | 0.60 | 1.17 | 1.00 | 0.04 | 0.05 |
| *S. xcinerea* | 25 | 8 | 16.00 | 16.00 | 16.00 | 1.60 | 1.60 | 1.00 | 1.00 | 0.10 | 0.10 |
| *S. xcinerea* | 25 | 9 | 16.00 | 16.00 | 14.00 | 1.00 | 1.00 | 1.00 | 0.88 | 0.06 | 0.06 |
| *S. xcinerea* | 25 | 10 | 16.00 | 16.00 | 14.00 | 1.60 | 1.60 | 1.00 | 0.88 | 0.10 | 0.10 |
| *S. xcinerea* | 25 | 11 | 14.00 | 14.00 | 12.00 | 1.60 | 1.60 | 1.00 | 0.86 | 0.11 | 0.11 |
| *S. xcinerea* | 25 | 12 | 16.00 | 14.00 | 16.00 | 2.00 | 2.00 | 1.14 | 1.00 | 0.13 | 0.14 |
| *S. xcinerea* | 25 | 13 | 14.00 | 14.00 | 12.00 | 1.60 | 1.60 | 1.00 | 0.86 | 0.11 | 0.11 |
| *S. xcinerea* | 25 | 14 | 16.00 | 14.00 | 16.00 | 1.60 | 1.60 | 1.14 | 1.00 | 0.10 | 0.11 |
| *S. xcinerea* | 25 | 15 | 14.00 | 14.00 | 12.00 | 1.60 | 1.60 | 1.00 | 0.86 | 0.11 | 0.11 |
| *S. xcinerea* | 25 | 16 | 16.00 | 14.00 | 14.00 | 1.00 | 1.00 | 1.14 | 0.88 | 0.06 | 0.07 |
| *S. xcinerea* | 25 | 17 | 16.00 | 14.00 | 14.00 | 1.00 | 1.00 | 1.14 | 0.88 | 0.06 | 0.07 |
| *S. xcinerea* | 25 | 18 | 16.00 | 14.00 | 16.00 | 0.60 | 0.60 | 1.14 | 1.00 | 0.04 | 0.04 |
| *S. xcinerea* | 25 | 19 | 14.00 | 12.00 | 14.00 | 1.60 | 1.60 | 1.17 | 1.00 | 0.11 | 0.13 |
| *S. xcinerea* | 25 | 20 | 16.00 | 14.00 | 16.00 | 1.00 | 1.00 | 1.14 | 1.00 | 0.06 | 0.07 |
| *S. xcinerea* | 25 | 21 | 14.00 | 14.00 | 12.00 | 1.00 | 1.00 | 1.00 | 0.86 | 0.07 | 0.07 |
| *S. xcinerea* | 25 | 22 | 16.00 | 14.00 | 14.00 | 1.00 | 1.00 | 1.14 | 0.88 | 0.06 | 0.07 |
| *S. xcinerea* | 25 | 23 | 16.00 | 14.00 | 16.00 | 0.60 | 0.60 | 1.14 | 1.00 | 0.04 | 0.04 |
| *S. xcinerea* | 25 | 24 | 16.00 | 14.00 | 16.00 | 1.60 | 1.60 | 1.14 | 1.00 | 0.10 | 0.11 |
| *S. xcinerea* | 25 | 25 | 16.00 | 14.00 | 14.00 | 1.60 | 1.60 | 1.14 | 0.88 | 0.10 | 0.11 |
| *S. xcinerea* | 25 | 26 | 16.00 | 14.00 | 14.00 | 0.60 | 0.60 | 1.14 | 0.88 | 0.04 | 0.04 |
| *S. xcinerea* | 25 | 27 | 16.00 | 14.00 | 16.00 | 1.60 | 1.60 | 1.14 | 1.00 | 0.10 | 0.11 |
| *S. xcinerea* | 25 | 28 | 16.00 | 14.00 | 14.00 | 1.00 | 1.00 | 1.14 | 0.88 | 0.06 | 0.07 |
| *S. xcinerea* | 25 | 29 | 16.00 | 16.00 | 16.00 | 2.00 | 2.00 | 1.00 | 1.00 | 0.13 | 0.13 |
| *S. xcinerea* | 25 | 30 | 16.00 | 14.00 | 16.00 | 1.00 | 1.00 | 1.14 | 1.00 | 0.06 | 0.07 |
