# Supplementary figures and images for: Pollen morphology and variability of native and alien, including invasive, species of the genus Spiraea L. (Rosaceae) in Poland
Source: PLoS One. 2022 Aug 29;17(8):e0273743. doi: 10.1371/journal.pone.0273743 (PMC9423682; doi:10.1371/journal.pone.0273743)

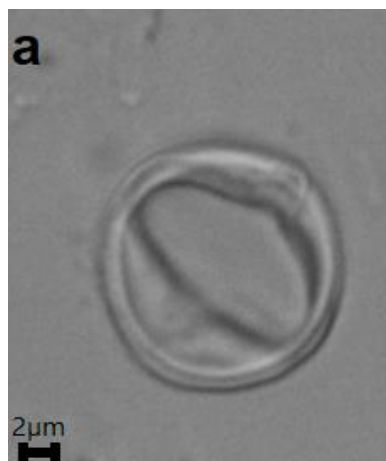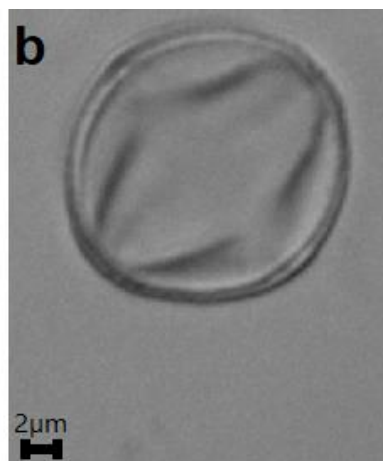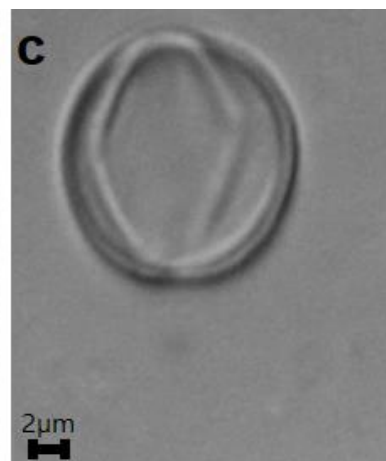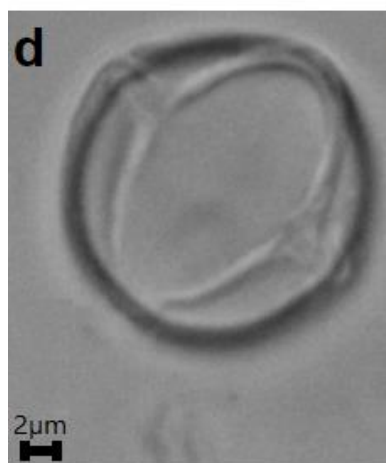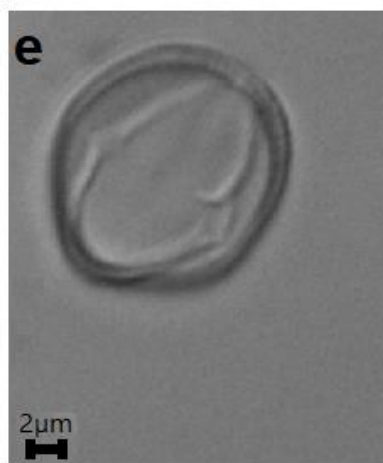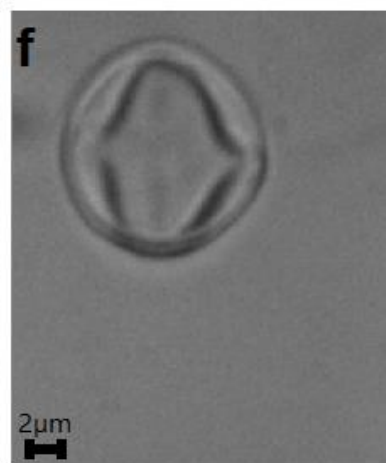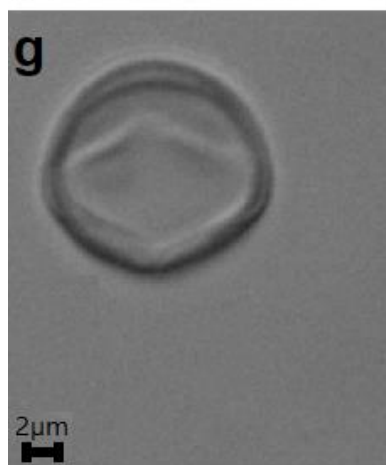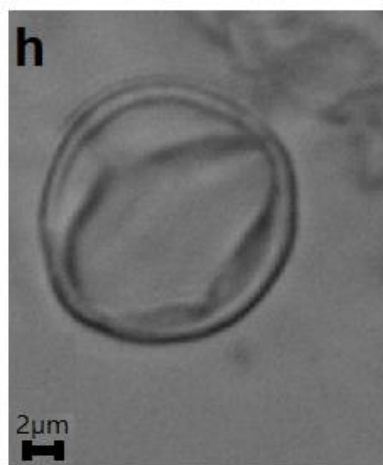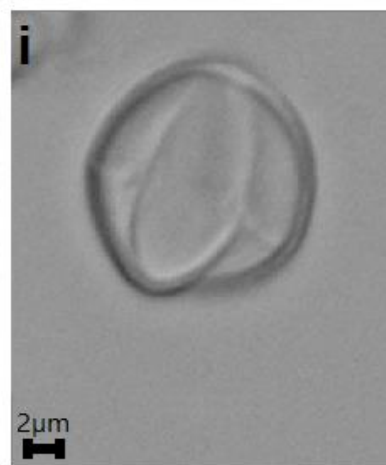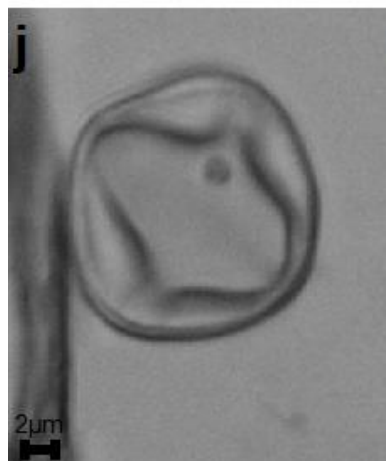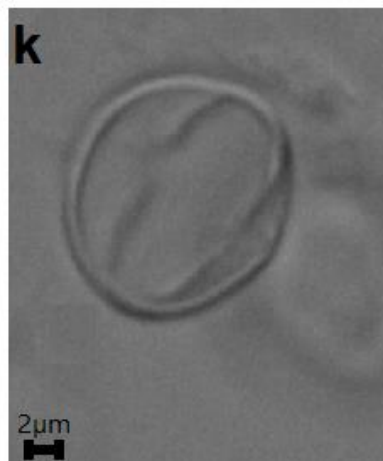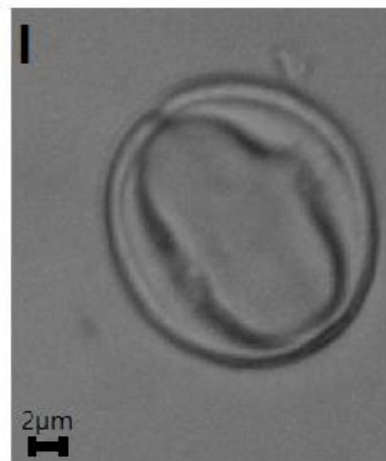

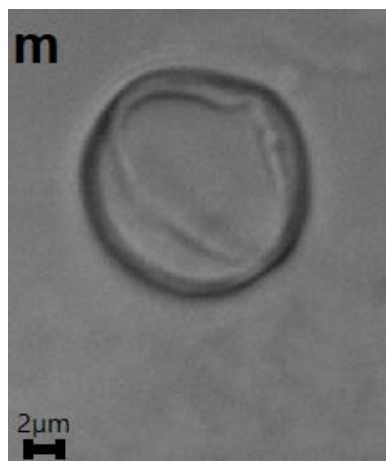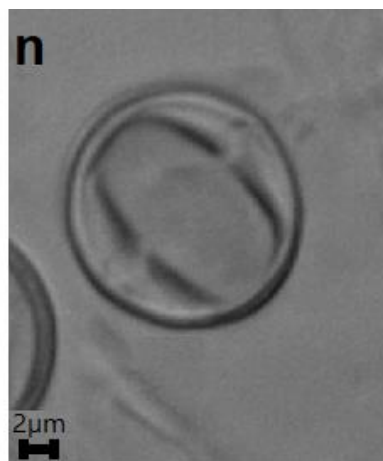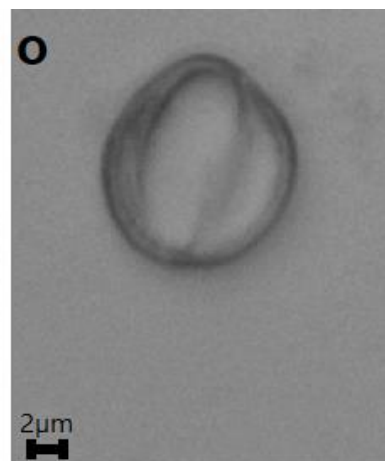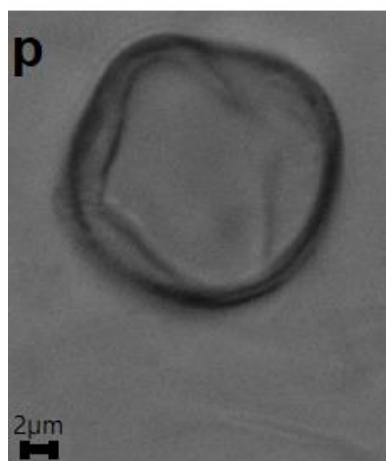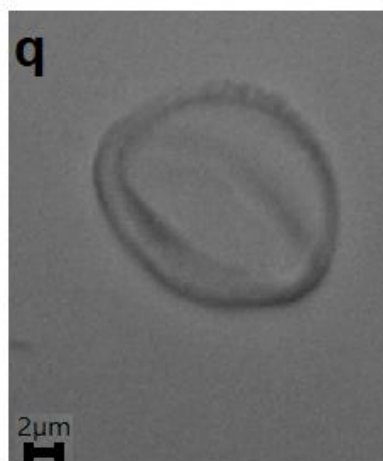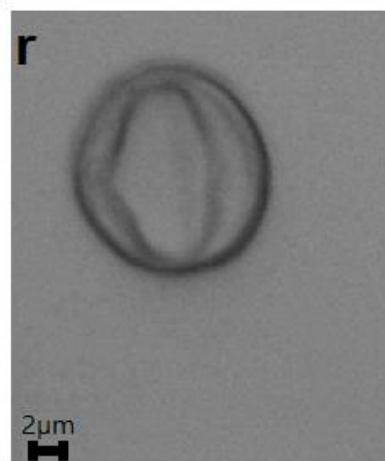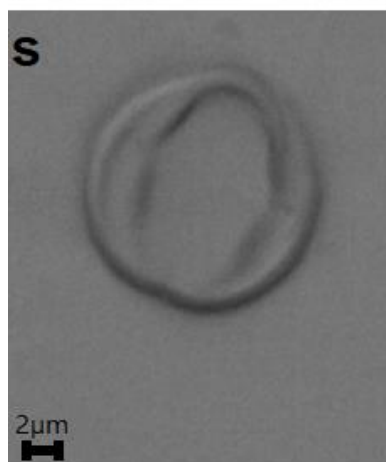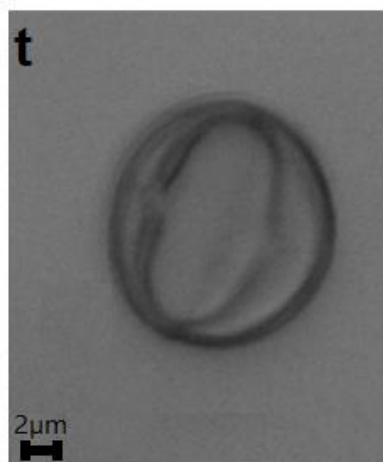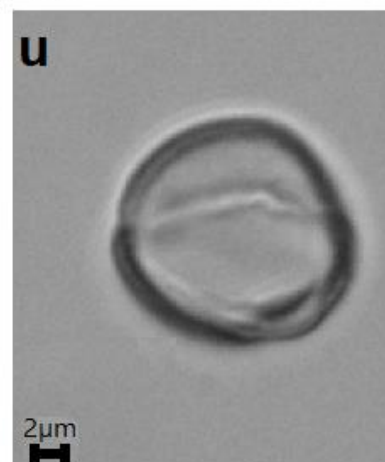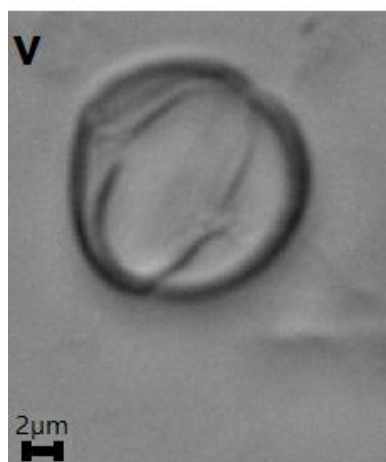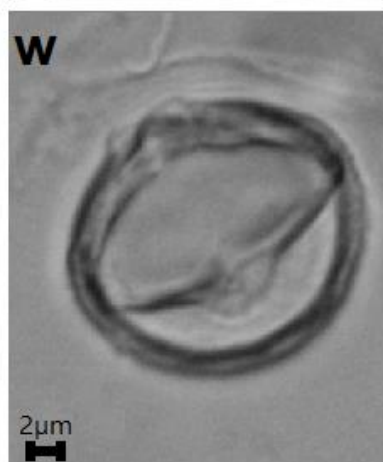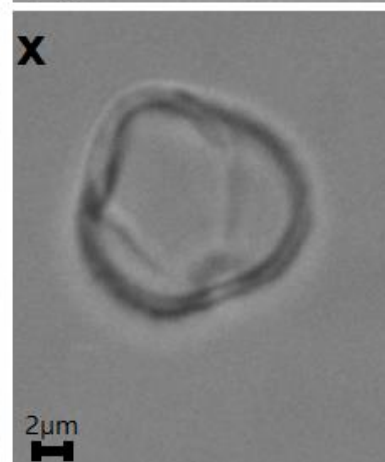

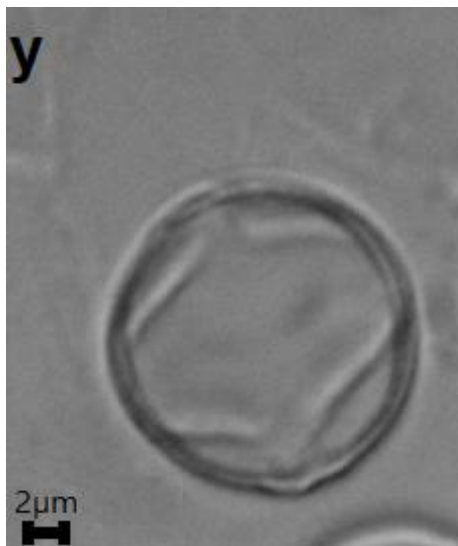

Supplement: S1 Fig — A, S. alba; B, S. betulifolia; C, S. cana; D, S. chamaedryfolia; E, S. chinensis; F, S. dasyantha; G, S. douglasii; H, S. elegans; I, S. henryi; J, S. hypericifolia; K, S. alba var. latifolia; L, S. media; M, S. media; N, S. nipponica; O, S. pubescens; P, S. salicifolia; Q, S. splendens; R, S. thunbergii; S, S. trichocarpa; T, S. uratensis; U, S. veitchii; V, S. wilsonii; W, S. ×billardii; X, S. ×cinerea; Y, S. tomentosa, A-Y. (PDF) [file pone.0273743.s002.pdf]
